# Gendered dimensions of sustainable land management: Evidences from farm size and training effects on willingness to pay in Ghana’s Volta Region

**DOI:** 10.1371/journal.pone.0351424

**Published:** 2026-06-11

**Authors:** James Dickson Fiagborlo, Mexoese Nyatuame, Stella Ahone Ntoko

**Affiliations:** 1 Department of Multidisciplinary Studies, Faculty of Applied Social Sciences, Ho Technical University, Ho, Ghana; 2 Department of Agric Engineering, Faculty of Engineering, Ho Technical University, Ho, Ghana; 3 Centre for Research in Efficient Agricultural Technologies (CREAT), Ho Technical University, Ho, Ghana; University of Illinois at Urbana-Champaign, UNITED STATES OF AMERICA

## Abstract

Land degradation is a critical threat to agricultural productivity in Ghana, reducing soil fertility and deepening rural poverty. Efforts to promote Sustainable Land Management (SLM) have increased, but adoption remains uneven, with persistent gender disparities in access to land, training, and decision-making. This study draws on survey data from 1,036 farming households across five municipalities in Ghana’s Volta Region to examine how SLM training and farm size affect farmers’ willingness to pay (WTP) for SLM practices, with a focus on gender differences. A Tobit regression model is used to estimate both main and interaction effects. The results support the study hypotheses. SLM training (β = 0.164, *p < 0.01*) significantly increases WTP, with stronger effects among male farmers, confirming H1. Farm size (β = 0.058, *p < 0.01*) is positively associated with WTP, as larger landholdings increase farmers’ investment capacity, supporting H2. Gender differences are evident: male farmers show higher baseline WTP, while female farmers show large increases in WTP as access to land and SLM training improves, partially supporting H3 and suggesting that disparities are driven mainly by structural constraints. The three-way interaction between SLM training, gender, and farm size is positive and significant (β = 0.375, *p < 0.05*), confirming H4 and indicating that the effect of SLM training on WTP depends jointly on gender and resource endowment. Overall, the findings suggest that gender is central to understanding investment decisions in SLM and highlight the need for gender-responsive policies that address inequalities in access to land, training, and productive resources.

## Introduction

Ghana’s agricultural landscape faces severe threats from deforestation, overgrazing, and uncontrolled burning, leading to widespread land degradation and declining productivity. Approximately 69% of the agricultural land is already eroded, costing the economy around US$ 4.2 billion annually, or about 5% of agricultural GDP [1,2]. Land degradation is deepening poverty by reducing soil fertility, lowering yields, and limiting farmers’ use of inputs such as fertiliser, irrigation, and mechanised tools [3]. In response, there is a renewed focus on Sustainable Land Management (SLM) as a key approach to restoring soil health and strengthening agricultural resilience [4]. According to TerrAfrica, SLM refers to land-use systems that enhance ecological support functions through proper management practices. This definition was expanded by [5] to include improving the productivity of crops, livestock, watersheds, and forests for sustainable agricultural land use.

Despite strong policy interest, Sustainable Land Management (SLM) practices remain limited in Ghana. Poor financial conditions, weak extension support, and limited access to technology constrain adoption, especially among smallholder farmers in the Volta Region [6,7]. Beyond these barriers, social and behavioural factors also play a role, as gender differences in access to training, land, and decision-making power shape SLM practices. Studies [8–10] show that farmers’ willingness to pay (WTP) for environmental and SLM practices depends on socioeconomic factors such as gender, age, education, income, and farm size. Evidence from other developing regions suggests that women face greater barriers to land ownership, credit, and training [11,12], yet the gender-specific dynamics of SLM practices and WTP remain understudied in Ghana.

In theory, gender differences in land and resource management can be explained by Human Capital Theory [13] and the Gender and Agricultural Decision-Making Framework [11]. These frameworks reveal that unequal access to knowledge, skills, and assets leads to unequal economic returns. This pattern is evident in practice, as women often face limited land tenure and restricted access to Sustainable Land Management (SLM) training and inputs, which constrain their investment in SLM [14,15]. This implies that women’s willingness to pay is less driven by resource endowment and is instead shaped by social norms, perceived risk, and intra-household decision-making. Empirical evidence supports this view, showing that agricultural decisions depend not only on resource availability but also on perceived efficacy and social learning [16,17].

Empirical studies from Africa [8,10] and Asia [12,15] confirm the importance of gender in sustainable agricultural practices. In Ethiopia, [8] found that male-headed households were more likely to invest in Sustainable Land Management (SLM) due to greater land ownership and better access to extension services. In Tanzania, [10] shows that women’s willingness to pay for watershed services was lower, reflecting gender-based financial barriers, but [15] reports that participation in environmental conservation programmes increased when training was designed to empower women. Similarly, [12] finds that gender-sensitive land governance frameworks in South Korea have strengthened women’s tenure rights and participation in land-use decisions. These findings underscore the need to examine how SLM training, access to land, and gender roles interact to shape SLM-related decisions.

The interaction of Sustainable Land Management (SLM) training with farm size and gender is not well understood in Ghana. SLM training aims to improve farmers’ technical and management knowledge [18]. However, its effects vary by gender due to unequal access to land and social capital. Male farmers often have larger farm sizes and greater financial independence. This allows them to apply knowledge gained from SLM training more easily. In contrast, female farmers may face constraints, even when they receive similar training. Understanding how SLM training and farm size affect willingness to pay (WTP) across gender groups is therefore essential for designing equitable agricultural interventions.

In light of these gaps, this study examines how gender shapes the relationship between Sustainable Land Management (SLM) training, farm size, and farmers’ willingness to pay (WTP) for SLM practices in Ghana’s Volta Region. It has three main objectives. First, it examines the effect of SLM training on WTP for SLM practices among male and female farmers. Second, it assesses the effect of farm size on WTP for SLM practices across gender groups. Third, it analyses gender differences in WTP for SLM practices, including the joint moderating effects of SLM training and farm size. This analysis is guided by four hypotheses derived from prior empirical studies.

This study’s main contribution is to demonstrate that gender is a central determinant of farmers’ willingness to pay (WTP). It shapes how Sustainable Land Management (SLM) training and farm size affect investment behaviour. Using an interaction-based approach, the study reveals that the effects of SLM training and farm size on WTP are not uniform but are moderated by gender and resource endowment. The findings indicate that gender differences in WTP stem mainly from structural inequalities, including unequal access to land, training, and productive resources, rather than differences in preferences. Male farmers show higher baseline WTP, while female farmers exhibit large increases in WTP when access to land and training improves. By examining the interaction between SLM training, gender, and farm size, the study provides new empirical evidence on the heterogeneous effects of SLM interventions and highlights the importance of gender-responsive and resource-sensitive policies for promoting inclusive and sustainable land management.

The paper is structured as follows: Section 2 presents a review of the literature on Sustainable Land Management and land policy in Ghana; Section 3 outlines the methodological framework; Section 4 presents and discusses the empirical results; and Section 5 concludes with policy implications, limitations, and recommendations for future research.

## Literature review

This section reviews land management policies in Ghana and synthesises empirical evidence on Sustainable Land Management (SLM) practices, SLM training, farm size, gender, and farmers’ willingness to pay. It draws on both African and global contexts to provide a thematically comprehensive foundation for the development of the hypotheses of this study.

### Land management techniques and policies in Ghana

Ghana’s National Land Policy, enacted in 1999, aims to create an efficient legal and administrative system for managing land rights [19,20]. It recognises traditional authorities and promotes sustainable land use amid population growth and rising land pressure. The policy addresses issues such as unregulated land markets, multiple sales, encroachment, and unplanned development. Its main goals are to ensure tenure security, improve productivity, and support sustainable land use [1,19]. Implementation includes national and district land-use plans as well as inter-ministerial groups for dispute resolution. It promotes inclusive decision-making with key stakeholders [21]. The policy prohibits forest clearing for mining, based on the idea that proper land-use planning reduces conflicts and improves ecological outcomes.

Other frameworks, such as the Forest and Wildlife Policy, Fire Policy, and Sustainable Land Management (SLM) Policy, support integrated natural resource management in agriculture [5]. However, implementation remains weak due to limited institutional capacity, poor enforcement, and weak coordination [22]. Farmers still use unsustainable practices, such as slash-and-burn cultivation, often driven by economic necessity without awareness of the long-term land degradation [23]. Programmes such as the Food and Agriculture Sector Development Policy (FASDEP), the Northern Rural Growth Programme, and the Ghana Strategic Investment Fund (GSIF) have supported SLM efforts, but limited community participation and gender-insensitive approaches reduce their impact [24]. Recent studies suggest multi-stakeholder, gender-inclusive land governance to integrate farmers, extension officers, and local institutions to promote long-term sustainability [6].

#### Sustainable land management, adoption, and training.

Empirical research across the African continent confirms that Sustainable Land Management (SLM) practices, such as conservation agriculture and agroforestry, improve yields, soil health, and climate resilience [4,25]. However, adoption remain low among smallholder farmers due to limited finance, weak extension services, and inadequate policy support [7]. Larger farms adopt more readily due to economies of scale, better access to credit, and lower transaction costs [8], while trained smallholder farmers also demonstrate higher practices [25]. Training is a key driver of SLM adoption and willingness to pay (WTP), as it enhances knowledge and awareness of long-term benefits [10,26], but access to training is often unequal. In many rural African contexts, men are more likely to receive training due to social norms fewer mobility constraints [11], and studies from Ethiopia [8] and Tanzania [10] show that lower WTP among women reflects structural barriers rather than lack of interest.

Evidence from Nepal [15] and South Korea [12] shows that gender-sensitive interventions improve women’s participation and land rights, yet male-headed households in Ethiopia and Kenya remain more likely to adopt capital-intensive Sustainable Land Management (SLM) practices due to better access to credit and mechanisation [14,25]. Behavioural factors also matter, as risk perceptions, trust, and social learning shape environmental decisions [16,17], with women often relying on community networks than individual resources. These findings highlight the need to address resource and behavioural constraints. Therefore, ***Hypothesis (H1)*** *states that farmers who receive SLM training will show higher willingness to pay for SLM practices, with effects differing by gender.*

#### Farm size, resource endowment, and willingness to pay.

Farm size is a key factor affecting willingness to pay (WTP). Larger farmers can spread the fixed costs over larger land, creating scale efficiencies [25]. Evidence from Nigeria [27], Egypt [28], and China [29] shows that farm size and income increase WTP for irrigation and soil management. However, very large farms may face diminishing returns to investment in Sustainable Land Management (SLM) practices [8]. SLM training interacts with farm size in shaping outcomes, as larger farmers are better able to apply knowledge from training, while smaller farmers may struggle due to financial constraint [25]. Gender also moderates this effect, as female farmers may not fully benefit from training if they lack access to inputs such as land, credit, and tools [11,15]. These findings suggest that farm size increases the capacity to invest in SLM, but strength of this effect varies by gender due to unequal access to resources. Consequently, ***Hypothesis (H2)*** *states that larger farm sizes are associated with higher WTP for SLM practices, with gender differences in magnitude.*

#### Gender, resource access, and investment behaviour.

Gender disparities in access to land, credit, training, and decision-making constrain Sustainable Land Management (SLM) practices. Female farmers often face institutional and socio-cultural barriers that reduce their ability to apply knowledge, even when they receive training [11,12]. Evidence shows that male farmers benefit more from agricultural interventions due to better access to productive assets and institutional support [14,25]. However, women’s participation in SLM increases when they gain equal access to resources, especially land and training. Studies such as [15] show that targeted support can improve their engagement in conservation practices. This suggests that gender differences in willingness to pay (WTP) are not inherent but are driven by structural inequalities. When these barriers are removed, female farmers may invest as much as or more than, male farmers in SLM practices. Thus, ***Hypothesis (H3)*** *states that female farmers with equal access to training and resources will show higher WTP for SLM practices, but may not steadily exceed male farmers in overall WTP.*

#### Interaction of SLM training, gender, and farm size.

Existing studies often examine training, farm size, and gender separately, but there is limited evidence on how these factors interact to influence willingness to pay for Sustainable Land Management practices. Recent research suggests that agricultural decisions are shaped by joint socioeconomic and institutional factors rather than isolated variables. The interaction between training and farm size suggests that knowledge applied more effectively when farmers have sufficient land [25], although the interaction between training and gender reflects unequal access to resources and institutional support [11,12]. However, these pairwise interactions do not fully capture the complexity of the farmers’ decision-making.

An intersectional perspective suggests that the interaction of training, gender, and farm size leads to different outcomes across farmer groups. Evidence shows that male farmers with larger landholdings derive the most from training, while female farmers’ willingness to pay (WTP) increases when they gain access to land and other productive resources [15]. Therefore, ***Hypothesis (H4)*** *states that the effect of Sustainable Land Management (SLM) training on WTP for SLM practices is jointly shaped by gender and farm size, producing heterogeneous effects across farmer groups.*

## Materials and methods

### Research design

This study uses a quantitative cross-sectional survey design to examine gender differences in Sustainable Land Management (SLM) training, farm size, and willingness to pay (WTP) for SLM practices among farming households in Ghana’s Volta Region. Cross-sectional designs are suitable for analysing relationships between socioeconomic characteristics and behavioural outcomes at a single point in time [30]. The study area, the Volta Region of Ghana, encompasses diverse ecological zones. These zones vary in rainfall, soil fertility, and access to extension services. Such differences are important for understanding variation in SLM adoption and valuation across farming households.

#### Sampling procedure.

A multistage sampling technique was used to ensure spatial representation [31]. In the first stage, five municipalities were purposively selected to reflect major ecological zones. The selection was based on differences in agro-climatic conditions and agricultural practices relevant to the adoption of Sustainable Land Management (SLM). In the second stage, five communities were randomly selected from each municipality, resulting in a total of 25 communities. In the final stage, 50 farming households were randomly selected from each community using local household lists. This produced an initial sample of 1,250 households. After data cleaning and removal of incomplete responses, the final sample comprised 1,036 households. This size is adequate for reliable econometric analysis [32,33]. While purposive selection improves ecological coverage, it may limit statistical generalisability.

#### Data collection.

This study used structured face-to-face interviews with adult members (aged 18 years and above) of farming households. Structure interviews are common in applied microeconomic and agricultural research. They improve the comparability and reliability of responses [34]. Interviews were conducted in English or Ewe by trained enumerators using a standardised questionnaire. The instrument was pre-tested to improve clarity, reliability, and relevance.

#### Ethical considerations.

Ethical standards for social science research were strictly followed [35]. Respondents were informed about the study’s purpose, the voluntary nature of participation, and their right to withdraw at any time. Due to literacy constraints, informed verbal consent was obtained and recorded prior to participation. All data were anonymised to ensure confidentiality.

#### Measurement of variables.

Table 1 presents the variables used in the analysis. The dependent variable is willingness to pay (WTP) for Sustainable Land Management (SLM) practices, measured as a non-negative continuous variable in Ghana Cedis (GHS). WTP was elicited using a contingent valuation (CV) method, a widely used approach for valuing non-market environmental goods [36,37].

**Table 1:** Measurement of variables.

Variable	Measurement	Min	Max
**WTP**	Continuous: non-negative censored variable (GHS)	0	500
**SLM training**	Binary: 1 = farmer received SLM training; 0 = otherwise	0	1
**Farm size**	Continuous: farm size in acres	1	45
**Gender**	Binary: 1 = male farmer; 0 = otherwise	0	1
**Coastal**	Binary: 1 = coastal resident; 0 = otherwise	0	1
**Income**	Continuous: monthly income (GHS)	30	1000
**Household dependant**	Continuous: number of household dependants	1	18
**Farmer experience**	Continuous: years of farming experience	1	60
**No education**	Binary: 1 = no formal education; 0 = otherwise	0	1
**Primary education**	Binary: 1 = primary education; 0 = otherwise	0	1
**Middle sch. education**	Binary: 1 = middle sch. education; 0 = otherwise	0	1
**Secondary education**	Binary: 1 = secondary education; 0 = otherwise	0	1
**Vocational education**	Binary: 1 = vocational training; 0 = otherwise	0	1
**Tertiary education**	Binary: 1 = tertiary education; 0 = otherwise	0	1

**NB: Max = Maximum; Min = Minimum.**

Respondents were given a hypothetical scenario describing the environmental benefits of SLM practices and asked to state the maximum amount they were willing to pay. The data are censored at zero, where zero values indicate non-participation. Potential biases, such as hypothetical and strategic bias, were reduced through careful design and pre-testing.

Key explanatory variables include Sustainable Land Management (SLM) training, farm size and gender. SLM training is a binary indicator (1 = participation; 0 = otherwise), farm size is measured in acres, and gender is coded as a binary variable (1 = male; 0 = female). The model controls for socioeconomic characteristics that may affect investment behaviour, including income, household dependants, farming experience, location, and education. Income is measured as monthly earnings in Ghana Cedis (GHS), household dependants represent the number of dependants, and farming experience is measured in years. Location is a binary variable (1 = coastal; 0 = inland), while education is represented by categorical dummy variables with one category as the reference group. Including these variables improves model specification and reduces omitted variable bias [34].

#### Econometric model.

Given the censored nature of Willingness to pay (WTP), a Tobit regression model is employed [38]. The Tobit model is appropriate for dependent variables that are continuous but limited at a threshold, allowing for the joint estimation of participation and intensity decisions [39].

The model is specified as follows:


$$\begin{array}{c} WTP_{i}*=\beta X_{i}\text{+}\varepsilon_{i}\; \end{array}$$ (1)

$$\begin{array}{c} \begin{array}{c} WTP_{i}=WTP_{i}*\;{\text{if WTP}}_{\text{i}}\text{*>0}\\ WTP_{i}{\text{= 0 \textasciitilde{}\textasciitilde{}\textasciitilde{}\textasciitilde{}\textasciitilde{}\textasciitilde{}\textasciitilde{}\textasciitilde{}\textasciitilde{}\textasciitilde{}\textasciitilde{}\textasciitilde{}if WTP}}_{\text{i}}\text{*}\leq \text{0} \end{array} \end{array}$$ (2)

where $WTP_{i}*$ represents the latent willingness to pay (WTP),$X_{i}$ is a vector of explanatory variables,β denotes the coefficients to be estimated, and $\varepsilon_{i}$ is the error term assumed to be independently and normally distributed with mean zero and constant variance.

The Tobit model assumes that the same process determines both the decision to pay and the amount paid. Alternative models, such as double-hurdle or sample selection models, relax this assumption, but the Tobit model is used as the baseline due to its simplicity and widespread use in willingness to pay studies [39,40]. To test robustness and address possible selection bias, additional estimations were conducted, including subsample analysis and a Heckman selection model. The results from these models are presented afterwards.

#### Interaction effects.

To capture variation in responses, the model includes interaction terms. These include two-way interactions between Sustainable Land Management (SLM) training and farm size, SLM training and gender, and gender and farm size. A three-way interaction among SLM training, gender, and farm size is also included. These terms allow the analysis of moderating effects and conditional relationships among key variables [41].

#### Estimation and post-estimation.

The model is estimated using Maximum Likelihood Estimation (MLE) [30]. Robust standard errors are applied to correct for heteroskedasticity [34]. To aid interpretation, average marginal effects (AMEs) are computed, as they provide intuitive estimates of the magnitude of relationships in non-linear models [42]. All estimations are conducted using Stata version 15.

#### Model diagnostics.

Diagnostic tests were conducted to assess multicollinearity and heteroskedasticity. Multicollinearity among explanatory variables was evaluated using the Variance Inflation Factors (VIF) and condition number. Table 2 shows that the highest VIF is 3.03 for middle school education, which is well below the common thresholds of 5 or 10 [39], indicating no serious multicollinearity. The condition number is 12.5, and the mean VIF is 1.51, both below the accepted threshold of 15, further confirming that multicollinearity is not a concern [43–46].

**Table 2:** Estimated VIF for the variables used in the analysis.

Variable	VIF	SqrtVIF	Tolerance	R-squared
**SLM training (slmt)**	1.08	1.04	0.929	0.071
**Farm size (fsize)**	1.23	1.11	0.815	0.185
**Gender (male)**	1.14	1.07	0.879	0.121
**Coastal**	1.17	1.08	0.855	0.145
**Income**	1.18	1.09	0.848	0.152
**Farmer experience**	1.08	1.04	0.927	0.073
**Household dependant**	1.06	1.03	0.948	0.052
**Primary education**	2.32	1.52	0.431	0.569
**Middle school education**	3.03	1.74	0.330	0.670
**Secondary education**	1.92	1.39	0.520	0.480
**Vocational education**	1.44	1.20	0.696	0.304
**Tertiary education**	1.45	1.20	0.691	0.309
**Mean VIF**		1.51		
**Breusch-Pagan/Cook-Weisberg test for heteroskedasticity**				
**Chi** ^**2**^		28.36		
**Prob> Chi** ^**2**^		0.000		

Heteroskedasticity was tested using the Breusch-Pagan or Cook-Weisberg test (Table 2), which showed non-constant error variance. To address this, robust standard errors were used in all estimations to ensure reliable statistical inference. Overall the diagnostic tests support the validity of the econometric model and strengthen confidence in the empirical results. The next section presents the summary statistics and empirical findings.

## Results and discussions

Table 3 presents descriptive statistics for the variables used in the analysis. The average willingness to pay (WTP) for SLM practices is GHS 76.59 (US$19.15)^1^. However, the median WTP is GHS 0, showing that more than half of the farmers are not willing to pay. This indicates that the mean is driven by smaller group of farmers with high willing to pay.

**Table 3:** Summary statistics.

**Variables**	**Observation**	**Mean**	**SD**
**Willingness to pay (WTP)**	1036	76.59	118.24
**SLM training (slmt)**	1036	0.35	0.48
**Farm size (fsize)**	1036	4.00	3.57
**Gender (male)**	1036	0.60	0.49
**coastal**	1036	0.54	0.50
**Income**	1036	346.62	207.62
**Household dependant**	1036	4.96	2.40
**Farmer experience**	1036	11.47	10.05
**No education**	1036	0.10	0.30
**Primary education**	1036	0.19	0.39
**Middle school education**	1036	0.52	0.50
**Secondary education**	1036	0.10	0.30
**Vocational education**	1036	0.05	0.21
**Tertiary education**	1036	0.04	0.19

NB: SD means standard deviation.

^1^Exchange rate is US$1 = GHS4—the average daily exchange at the time of data collection (October 2016 to November 2016). This was retrieved from the Bank of Ghana at https://www. bog. gov.gh/treasury- and- the- markets/ historical-interbank-fx-rates/.

To estimate aggregate willingness to pay (WTP), the mean WTP was multiplied by the number of agricultural households. According to the 2010 Population and Housing Census, there are about 201,800 agricultural households in the Volta Region and about 2.4 million in Ghana. This gives an aggregate WTP of GHS 15.46 million (US$ 3.87 million) for the region and GHS182.41 million (US$ 45.60 million) nationally. These values reflect the economic importance farmers place on the environmental benefits of Sustainable Land Management.

The average willingness to pay (GHS 76.59) is about 19% of farmers’ monthly income. This suggests that farmers who are willing to pay perceive Sustainable Land Management as economically valuable. However, these estimates should be interpreted with caution. They are based on stated preferences. Actual payments may differ due to budget constraints and behavioural or contextual factors. Table 3 further reveal that 35% of farmers received Sustainable Land Management training, while 54% reside in coastal areas. The average monthly income is GHS346.62 (US$86.66), while male farmers constitutes 60% of the sample. On average, farmers have five dependants and 12 years of farming experience. Educational attainment is relatively high, with only 10% of farmers having no formal education, while the average farm size is four acres.

**Fig 1:**
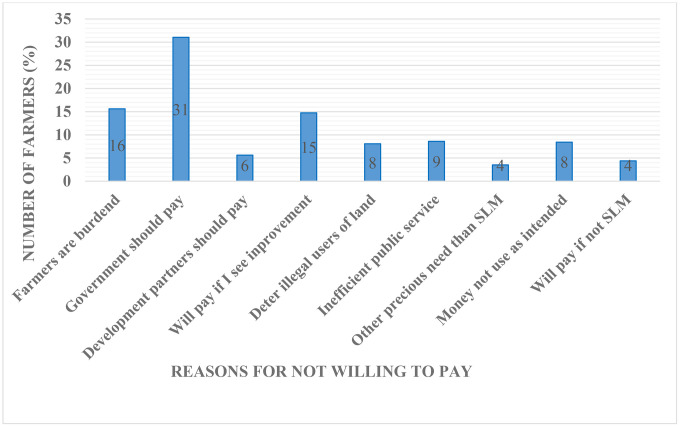
Reasons for not being willing to pay for SLM practices.

Fig 1 reveals that reluctance to pay is driven primarily by expectations of government responsibility, high farm costs, and low institutional trust. Nearly 31% of farmers believe the government should provide funding, while 16% cite financial constraint. In addition, 15% report willingness to pay (WTP) only if they see tangible improvements, and 9% express distrust in public institutions. These findings highlight the role of trust and perceived effectiveness in shaping WTP and are consistent with evidence that institutional credibility influences willingness to contribute to environmental services [47,48].

### Empirical results

Table 4 presents the average marginal effects (AMEs) from eight models. Model 1 reports the baseline Tobit specification, while Models 2 and 3 apply a two-part model as a robustness check. Model 2 estimates the probability of paying using a logit model, and Model 3 estimates the level of willingness to pay (WTP) among positive responses using OLS. Models 4 and 5 present separate Tobit estimates for male and female farmers, while Models 6 and 7 present separate Tobit estimates for farmers with low and high farming experience. In addition, a Heckman selection model is estimated (S2 Table) to correct for possible selection bias from the decision to report a positive WTP amount.

**Table 4:** Average marginal effects of predictor variables on WTP for SLM practices.

Variables	Model 1	Model 2	Model 3	Model 4	Model 5	Model 6	Model 7
**SLM training (slmt)**	0.164^***^	0.116^***^	0.537^***^	0.107^***^	0.282^***^	0.145^***^	0.170^***^
	(0.029)	(0.033)	(0.118)	(0.038)	(0.044)	(0.042)	(0.041)
**Log of farm size (lfsize)**	0.058^***^	0.074^***^	−0.011	0.054^**^	0.067^**^	0.111^***^	0.021
	(0.020)	(0.022)	(0.089)	(0.024)	(0.033)	(0.027)	(0.027)
**Gender (male)**	0.030	0.007	0.220^*^	–	–	0.015	0.036
	(0.027)	(0.032)	(0.114)	–	–	(0.037)	(0.040)
**slmt x lfsize**	0.414^**^	0.548^***^	2.010^***^	0.310	0.685^***^	0.378^**^	0. 468^**^
	(0.142)	(0.029)	(0.112)	(0.207)	(0.176)	(0.197)	(0.203)
**slmt x male**	0.315^*^	0.512^***^	2.062^***^	–	–	0.300	0.291
	(0.174)	(0.035)	(0.133)	–	–	(0.280)	(0.220)
**lfsize x male**	–0.167	0.459^***^	1.749^***^	–	–	–0.117	–0.222
	(0.110)	(0.021)	(0.089)	–	–	(0.156)	(0.156)
**slmt x male x lfsize**	0.375^**^	0.522^***^	2.089^***^	–	–	0.290	0. 438^*^
	(0.196)	(0.038)	(0.157)	–	–	(0.299)	(0.258)
**Coastal**	–0.169^***^	–0.214^***^	–0.038	–0.222^***^	–0.070	–0.208^***^	–0.123^***^
	(0.028)	(0.029)	(0.128)	(0.035)	(0.044)	(0.038)	(0.040)
**Log of income**	0.046^**^	0.039^*^	0.121	0.065^**^	0.004	0.035	0.065^**^
	(0.019)	(0.023)	(0.077)	(0.026)	(0.028)	(0.025)	(0.030)
**Log of experience**	–0.001	–0.009	0.036	0.007	–0.016	–	–
	(0.016)	(0.018)	(0.067)	(0.020)	(0.025)	–	–
**Household dependant**	–0.066^**^	–0.080^***^	0.000	–0.042	–0.085^**^	–0.082^**^	–0.033
	(0.026)	(0.028)	(0.115)	(0.034)	(0.038)	(0.036)	(0.036)
**Primary education**	0.043	0.000	0.377^*^	0.086	0.016	–0.005	0.068
	(0.049)	(0.056)	(0.229)	(0.076)	(0.065)	(0.071)	(0.065)
**Middle sch. education**	0.049	0.034	0.234	0.107	0.009	–0.017	0.090
	(0.043)	(0.049)	(0.201)	(0.067)	(0.059)	(0.063)	(0.055)
**Secondary education**	–0.008	–0.108	0.765^***^	0.129	–0.354^***^	–0.113	0.078
	(0.064)	(0.068)	(0.292)	(0.084)	(0.105)	(0.089)	(0.085)
**Vocational education**	–0.193^**^	–0.244^***^	0.098	–0.220^*^	–0.103	–0.143	–0.188*
	(0.083)	(0.090)	(0.322)	(0.127)	(0.113)	(0.123)	(0.107)
**Tertiary education**	–0.050	–0.061	0.060	0.020	–0.172	–0.122	–0.013
	(0.076)	(0.094)	(0.315)	(0.092)	(0.189)	(0.105)	(0.100)
**Observations**	1,036	1,036	472	622	414	504	532
**Log/pseudolikelihood**	–1337.92	–649.052	–	–815.154	–507.002	–651.142	–677.88
**LR /Wald chi** ^**2**^	–	114.22	–	–	–	–	–
**F(x, n)**	8.51		3.81	8.59	4.86	5.62	4.60
**Pseudo R** ^**2**^ **/R** ^**2**^	0.050	0.091	0.10	0.06	0.06	0.06	0.05
**Prob> F**	0.000	0.000	0.000	0.000	0.000	0.000	0.000

Robust standard errors in parentheses *** *p < 0.01*, ** *p < 0.05*, * *p < 0.1.*

NB: Model 1 = baseline model (Tobit); Models 2&3 = Two-part model (Logit &OLS) for robustness check; Models 4 &5 = gendered subsample model (Tobit); Models 6&7 = Farmer experience subsample model (Tobit).

The model summary statistics show that all estimated models are significant. The F-statistics range from 3.81 to 8.52, with p-values of 0.000, confirming overall model significance [49,50]. The pseudo-R2/R2 values range from 0.05 to 0.10. This indicates that the models explain 5% to 10% of the variations in willingness to pay. Although these values are modest, they are typical for limited dependent variable models such as Tobit and logit. Model 1 uses 1,036 observations, while the subsample models vary by group sizes (Table 4).

#### Robustness, heterogeneity, and endogeneity considerations.

Robustness and validity checks were conducted to strengthen confidence in the baseline findings. Table 4 shows that the direction and significance of key predictor, especially Sustainable Land Management (SLM) training and farm size, remain consistent across model specifications, confirming the robustness of the baseline Tobit estimates. Subsample analyses (Models 4–7) reveal important heterogeneity, as the effects of SLM training and farm size vary by gender and farming experience, with stronger marginal effects of SLM training among less-experienced and female farmers, highlighting the need for targeted capacity-building interventions [16,25]. The Heckman selection model reports a significant Inverse Mills Ratio (IMR = –1.411, *p < 0.05*; S2 Table), indicating selection bias in the baseline Tobit model and justifying the use of a correction. However, the sign and magnitudes of the main variables remain stable across models, reinforcing the robustness and internal consistency of the results. The next section presents the baseline Tobit model (Model 1) in greater detail.

#### Main, interaction, and total marginal effects.

This section addresses each hypothesis. It presents Tobit estimates of the main, interaction, and total marginal effects of Sustainable Land Management (SLM) training, farm size, and gender on farmers’ willingness to pay for SLM practices. The results are in line with previous studies. These studies highlight the relevance of training, resource endowment, and socioeconomic characteristics in shaping investment decisions in sustainable agriculture [11,25].

#### Effect of SLM training and gender differences (H1).

In Table 4, Model 1, the coefficient on Sustainable Land Management (SLM) training is positive and statistically significant (β = 0.164, *p < 0.01*). This shows that farmers who receive SLM training are more willing to invest in SLM practices. The result aligns with evidence that training enhances knowledge, reduces uncertainty, and increases awareness of long-term benefits of land management [10,26]. The interaction between SLM training and gender is also positive and marginally significant (β = 0.0.315, *p < 0.1*). This suggests that male farmers benefit more from SLM training than female farmers, likely because men have better access to resources such as land, credit, and extension services, which help them translate training into investment more effectively [11,12,25]. Furthermore, total marginal effects (S1 Table) show that SLM training increases willingness to pay (WTP) across all farmer groups. However, the effect is strongest among male farmers with larger landholdings. This reflects persistent gender disparities in access to productive resources [11,15]. These findings provide strong support for **H1**, that SLM training increases WTP, with effects differing by gender.

#### Effect of farm size and its interaction with SLM training (H2).

Farm size has a positive and significant association with willingness to pay (WTP) (β = 0.058, *p < 0.01*; Table 4, Model 1). This suggests that farmers with larger landholdings are better able to invest in Sustainable Land Management (SLM), consistent with economies-of-scale arguments [25,27,28]. The interaction between SLM training and farm size is also positive and significant (β = 0.414, *p < 0.05;* Table 4, Model 1), indicating that training becomes more effective as farm size increases and that resource endowment improves farmers’ ability to apply training [25]. The interaction between farm size and gender is negative and not statistically significant (β = –0.167, *p < 0.1*; Table 4, Model 1). However, the marginal effects (S1 Table) show differences across gender groups. Male farmers have higher overall WTP (0.680), while female farmers exhibit substantial gains (0.472) as access to land improves, reflecting gender-based disparities in access to productive resources [11,15]. Overall, these results support **H2**, confirming that farm size increases WTP and that its effects vary by gender.

#### Gender, resource access, and differential investment behaviour (H3).

The results show that gender differences are evident but not uniform. Male farmers have higher baseline willingness to pay (WTP), while female farmers show significant increases in WTP when training and access to land improve (see S1 Table). This partially supports **H3**, suggesting that gender gaps in WTP are driven by structural inequalities in access to productive resources rather than differences in preferences [11,12,15]. Even so, male farmers still exhibit higher overall WTP (S1 Table), indicating that improved access to resources reduces, but does not eliminate, the gender gap [14,25].

#### Moderating effects of SLM training, gender, and farm size (H4).

In Table 4, the three-way interaction term is positive and significant in Model 1 (β = 0.375, *p < 0.05*), confirming **H4**. This suggests that the effect of Sustainable Land Management (SLM) training on willingness to pay (WTP) is jointly shaped by gender and farm size and varies across farmer groups. Male farmers with larger landholdings gain the most from SLM training, while female farmers show strong increases in WTP as access to land improves (S1 Table). This finding aligns with research on intersectionality in agricultural decision-making and shows how multiple socioeconomic factors interact to shape behaviour [11,15,25].

**Fig 2:**
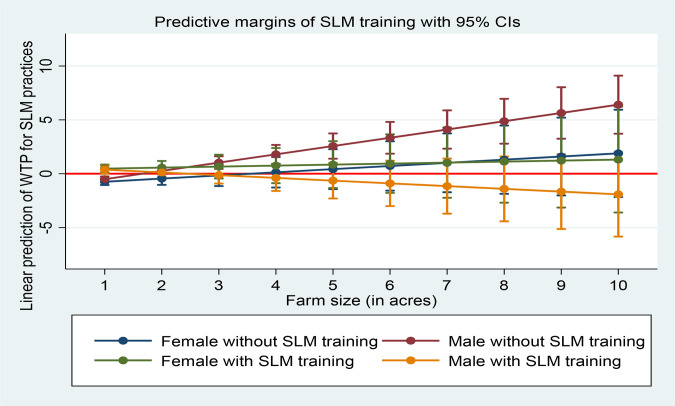
Predictive margins of SLM training by gender and farm size.

Fig 2 supports the estimates in S1 Table. It reveals that willingness to pay (WTP) increases with farm size among trained female farmers, while trained male farmers exhibit a decline in WTP as farm size increases. This suggests diminishing returns to training for male farmers who already have more resources and stronger benefits for resource-constrained groups, especially women and smallholders [15]. Overall, Fig 2 confirms that the effect of SLM training is not uniform but depends on both gender and farm size. Training helps offset resource constraints among female farmers, but it may substitute for resource advantages among male farmers with larger landholdings.

#### Effects of covariates.

Several covariates provide additional insights into willingness to pay (WTP). Income has a positive and significant association with WTP (β = 0.046, *p < 0.05;* Table 4, Model 1). This indicates that the higher-income farmers are more willing to invest in Sustainable Land Management practices. This finding is consistent with the “worth effect” in the Human Capital Theory [13] and is supported by evidence from environmental economics [18].

In contrast, other covariates exhibit negative or mixed effects on willingness to pay (WTP). Coastal lactation has a negative and significant association with WTP (β = –0.169, *p < 0.01;* Table 4, Model 1), indicating that coastal farmers in coastal areas are less willing to pay for Sustainable Land Management (SLM) practices, likely due to alternative livelihoods such as fishing. Household dependency ratios also shows a negative and significant effect on WTP (β = –0.073, *p < 0.05*), suggesting that larger households face resource constraints that limit SLM investment, consistent with prior studies [10,27]. The effect of education is mixed. Lower education levels show no significant relationship, but vocational education has a negative and significant association with WTP (β = –0.193, *p < 0.05*). This suggests that vocationally trained farmers may rely less on agriculture and have weaker incentives to invest in SLM. These findings support [14], which argues that general education does not promote pro-environmental behaviour unless it includes sustainability content.

## Conclusions

This study examines how gender shapes the relationship between Sustainable Land Management (SLM) training, farm size, and farmers’ willingness to pay (WTP) for SLM practices in Ghana’s Volta Region. The results show that both SLM training and farm size significantly relate to WTP. However, these relationships differ by gender. Male farmers have higher baseline WTP, especially when they have larger landholdings and access to training. In contrast, female farmers show substantial increases in WTP when access to land and training improves. This suggests that gender gaps in SLM investment are driven mainly by structural constraints rather than differences in preferences. These findings align with Human Capital Theory (HCT), which highlights the productivity gains from training, and Behavioural Economics, which underscores the role of social norms, financial constraints, and perceived risks on decision-making.

The findings highlight that gender is central to farmers’ investment decisions in Sustainable Land Management (SLM). Policy interventions should prioritise gender-responsive approaches that improve women’s access to land, training, and resources. The values of the estimated WTP also provide useful inputs for participatory land valuation, as they reflect the perceived economic value of environmental services from SLM practices.

This study has several limitations. The use of cross-sectional data limits causal inference, and the focus on five municipalities in the Volta Region may limit generalisability. The analysis does not fully capture intra-household dynamic decision-making, and the reliance on stated preferences may introduce hypothetical bias. Despite these limitations, the results are robust across Tobit, Two-part, and Heckman models, suggesting that the findings are not model-specific. Future research should employ longitudinal or experimental designs and further explore the gendered decision-making in agricultural investment more closely, for example, using instrumental variable Tobit models. Such approaches would strengthen the credibility and policy relevance of causal claims.

## Supporting information

S1 Table Total marginal effects of key predictors on WTP for SLM practices. (DOCX)

S2 Table Heckman selection model as a robustness check for baseline Tobit model. (DOCX)

S1 File Data. (XLS)
