# Supplementary material for: Gendered dimensions of sustainable land management: Evidences from farm size and training effects on willingness to pay in Ghana’s Volta Region
Source: PLoS One. 2026 Jun 11;21(6):e0351424. doi: 10.1371/journal.pone.0351424 (PMC13257986; doi:10.1371/journal.pone.0351424)
Supplement: S1 Table — (DOCX) [file pone.0351424.s001.docx]

**Appendix**

| **S1 Table. Total marginal effects of key predictors on WTP for SLM practices** | | | | | |
| --- | --- | --- | --- | --- | --- |
| ***A: Total Marginal effects of SLM training on WTP for SLM practices by farm size and gender*** | | | | | |
| ***Farmer Type*** | **B1** | **B4** | **B5** | **B7** | **Total marginal effects** |
| **male=0 (lfsize=1)** | 0.164 | 0.414 | 0 | 0 | 0.578 |
| **male=1 (lfsize =1)** | 0.164 | 0.414 | 0.315 | 0.375 | 1.268 |
| **male=0 (lfsize =0)** | 0.164 | 0 | 0 | 0 | 0.164 |
| **male=1 (lfsize =0)** | 0.164 | 0 | 0.315 | 0 | 0.479 |
| ***B: Total Marginal effects of farm size on WTP for SLM practices by SLM training and gender*** | | | | | |
| ***Farmer Type*** | **B2** | **B4** | **B6** | **B7** | **Total marginal effects** |
| **male=0 (slmt=1)** | 0.058 | 0.414 | 0 | 0 | 0.472 |
| **male=1 (slmt=1)** | 0.058 | 0.414 | –0.167 | 0.375 | 0.680 |
| **male=0 (slmt=0)** | 0.058 | 0 | 0 | 0 | 0.058 |
| **male=1 (slmt=0)** | 0.058 | 0 | –0.167 | 0 | –0.109 |
| **B: Total Marginal effects of gender on WTP for SLM practices by SLM training and farm size** | | | | | |
| ***Farmer Type*** | **B3** | **B5** | **B6** | **B7** | **Total marginal effects** |
| **slmt=0 (lfsize =1)** | 0.03 | 0 | –0.167 | 0 | –0.137 |
| **slmt=1 (lfms =1)** | 0.03 | 0.315 | –0.167 | 0.375 | 0.553 |
| **slmt=0 (lfsize =0)** | 0.03 | 0 | 0 | 0 | 0.03 |
| **slmt=1 (lfsize =0)** | 0.03 | 0.315 | 0 | 0 | 0.345 |

*NB: B1=main effect of SLM training; B2=main effect of farm size; B3=main effect of gender; B4= slmt x lfsize; B5= slmt x male; B6= lfsize x male; B7= slmt x male x lfsize*
