# Supplementary material for: Gendered dimensions of sustainable land management: Evidences from farm size and training effects on willingness to pay in Ghana’s Volta Region
Source: PLoS One. 2026 Jun 11;21(6):e0351424. doi: 10.1371/journal.pone.0351424 (PMC13257986; doi:10.1371/journal.pone.0351424)
Supplement: S2 Table — (DOCX) [file pone.0351424.s002.docx]

**Appendix B**

| **S2 Table. Heckman selection model as a robustness check for baseline Tobit model** | | | |
| --- | --- | --- | --- |
| **Variables** | **Tobit model** | **Heckman eqn. 1** | **Heckman eqn. 2** |
| **SLM training (slmt)** | 0.164^***^ |  | 0.113^***^ |
|  | (0.029) |  | (0.0325) |
| **Log of farm size (lfsize)** | 0.058^***^ | 0.0858^***^ | –0.176 |
|  | (0.020) | (0.0213) | (0.111) |
| **Gender (male)** | 0.030 | –0.0321 | 0.0917^*^ |
|  | (0.027) | (0.0314) | (0.0485) |
| **slmt x lfsize** | 0.414^**^ | - | 0.548^***^ |
|  | (0.142) | - | (0.029) |
| **slmt x male** | 0.315^*^ | - | 0.512^***^ |
|  | (0.174) | - | (0.035) |
| **lfsize x male** | –0.167 | 0.329^***^ | 0.459^***^ |
|  | (0.110) | (0.020) | (0.021) |
| **slmt x male x lfsize** | 0.375^**^ | - | 0.522^***^ |
|  | (0.196) | - | (0.039) |
| **Coastal** | –0.169^***^ | –0.142^***^ | 0.184 |
|  | (0.028) | (0.0292) | (0.177) |
| **Log of income** | 0.046^**^ | 0.0372^*^ | –0.0652 |
|  | (0.019) | (0.0225) | (0.0508) |
| **Log of experience** | –0.001 | 0.0123 | - |
|  | (0.016) | (0.0180) | - |
| **Household dependant** | –0.066^**^ | 0.00865 | –0.108^***^ |
|  | (0.026) | (0.0273) | (0.0295) |
| **Primary education** | 0.043 | 0.0347 | –0.0884 |
|  | (0.049) | (0.0569) | (0.0688) |
| **Middle sch. education** | 0.049 | 0.0409 | –0.0694 |
|  | (0.043) | (0.0511) | (0.0685) |
| **Secondary education** | –0.008 | 0.151^**^ | –0.498^***^ |
|  | (0.064) | (0.0646) | (0.185) |
| **Vocational education** | –0.193^**^ | 0.110 | –0.520^***^ |
|  | (0.083) | (0.0783) | (0.154) |
| **Tertiary education** | –0.050 | 0.190^**^ | –0.536^**^ |
|  | (0.076) | (0.0895) | (0.227) |
| **Inverse mills ratio (IMR)** |  |  | –1.411^**^ |
| Observations | 1,036 | 1,036 | 1,036 |

**Standard errors in parentheses ^***^ p<0.01, ^**^ p<0.05, ^*^ p<0.1**
