# Supplementary material for: Gendered dimensions of sustainable land management: Evidences from farm size and training effects on willingness to pay in Ghana’s Volta Region
Source: PLoS One. 2026 Jun 11;21(6):e0351424. doi: 10.1371/journal.pone.0351424 (PMC13257986; doi:10.1371/journal.pone.0351424)
Supplement: Attachment — Submitted filename: PONE-D-25-40765_reviewer comments edg.pdf [file pone.0351424.s004.pdf]

PLOS ONE

Gendered dimensions of sustainable land management: evidence of farm size and training effects on willingness to pay in Ghana's Volta Region

--Manuscript Draft--

**Manuscript Number:**	PONE-D-25-40765
**Article Type:**	Research Article
**Full Title:**	Gendered dimensions of sustainable land management: evidence of farm size and training effects on willingness to pay in Ghana's Volta Region
**Short Title:**	Gendered dimensions, sustainable land management training, farm size and willingness to pay
**Corresponding Author:**	James Dickson Fiagborlo<br>Ho Technical University<br>Ho, GHANA
**Keywords:**	Gender differences. Farm size. SLM training. Tobit model. Volta Region. Willingness to pay
**Abstract:**	This study investigates gender differences in sustainable land management (SLM) training and farm size with respect to willingness to pay (WTP) for agricultural SLM practices in Ghana's Volta Region. Employing a Tobit regression model on primary data collected from five municipalities, the findings reveal that both SLM training and farm size have a statistically significant positive effect on WTP. Notably, these effects are further influenced by gender. While SLM training appears to reduce WTP among male farmers as farm size increases, female farmers—irrespective of training—demonstrated a higher WTP than their male counterparts with training. Additionally, the study finds that residing in coastal communities, having more dependants, lower income, and vocational education are negatively associated with WTP for SLM practices. These findings underscore the importance of tailoring SLM interventions to the gender-specific needs and conditions of farmers in the Volta Region.
**Order of Authors:**	James Dickson Fiagborlo
	Mexoese Nyatuame
	Stella Ahone Ntoko
**Additional Information:**	
**Question**	**Response**
**Financial Disclosure**<br><br>Enter a financial disclosure statement that describes the sources of funding for the work included in this submission. Review the submission guidelines for detailed requirements. View published research articles from *PLOS ONE* for specific examples.<br><br>This statement is required for submission and **will appear in the published article** if the submission is accepted. Please make sure it is accurate.	The author(s) received no specific funding for this work.

Powered by Editorial Manager® and ProduXion Manager® from Aries Systems Corporation

**Funded studies**<br>Enter a statement with the following details:<br>• Initials of the authors who received each award<br>• Grant numbers awarded to each author<br>• The full name of each funder<br>• URL of each funder website<br>• Did the sponsors or funders play any role in the study design, data collection and analysis, decision to publish, or preparation of the manuscript?<br><br>Did you receive funding for this work?	
**Competing Interests**<br><br>Use the instructions below to enter a competing interest statement for this submission. On behalf of all authors, disclose any competing interests that could be perceived to bias this work—acknowledging all financial support and any other relevant financial or non-financial competing interests.<br><br>This statement is required for submission and **will appear in the published article** if the submission is accepted. Please make sure it is accurate and that any funding sources listed in your Funding Information later in the submission form are also declared in your Financial Disclosure statement.<br><br>View published research articles from *PLOS ONE* for specific examples.	The authors have declared that no competing interests exist.

**NO authors have competing interests**<br><br>Enter: *The authors have declared that no competing interests exist.*<br><br>**Authors with competing interests**<br><br>Enter competing interest details beginning with this statement:<br><br>*I have read the journal's policy and the authors of this manuscript have the following competing interests: [insert competing interests here]*<br><br>* typeset	
**Ethics Statement**<br><br>Enter an ethics statement for this submission. This statement is required if the study involved:<br><br>• Human participants<br>• Human specimens or tissue<br>• Vertebrate animals or cephalopods<br>• Vertebrate embryos or tissues<br>• Field research<br><br>Write "N/A" if the submission does not require an ethics statement.<br><br>General guidance is provided below. Consult the submission guidelines for detailed instructions. **Make sure that all information entered here is included in the Methods section of the manuscript.**	N/A

**Format for specific study types**<br><br>**Human Subject Research (involving human participants and/or tissue)**<br>• Give the name of the institutional review board or ethics committee that approved the study<br>• Include the approval number and/or a statement indicating approval of this research<br>• Indicate the form of consent obtained (written/oral) or the reason that consent was not obtained (e.g. the data were analyzed anonymously)<br><br>**Animal Research (involving vertebrate animals, embryos or tissues)**<br>• Provide the name of the Institutional Animal Care and Use Committee (IACUC) or other relevant ethics board that reviewed the study protocol, and indicate whether they approved this research or granted a formal waiver of ethical approval<br>• Include an approval number if one was obtained<br>• If the study involved *non-human primates*, add *additional details* about animal welfare and steps taken to ameliorate suffering<br>• If anesthesia, euthanasia, or any kind of animal sacrifice is part of the study, include briefly which substances and/or methods were applied<br><br>**Field Research**<br><br>Include the following details if this study involves the collection of plant, animal, or other materials from a natural setting:<br>• Field permit number<br>• Name of the institution or relevant body that granted permission	
**Data Availability**<br><br>Authors are required to make all data underlying the findings described fully available, without restriction, and from the time of publication. PLOS allows rare exceptions to address legal and ethical concerns. See the PLOS Data Policy and FAQ for detailed information.	No - some restrictions will apply

A Data Availability Statement describing where the data can be found is required at submission. Your answers to this question constitute the Data Availability Statement and **will be published in the article**, if accepted.<br><br>**Important:** Stating 'data available on request from the author' is not sufficient. If your data are only available upon request, select 'No' for the first question and explain your exceptional situation in the text box.<br><br>Do the authors confirm that all data underlying the findings described in their manuscript are fully available without restriction?	
**Describe where the data may be found in full sentences. If you are copying our sample text, replace any instances of XXX with the appropriate details.**<br><br>• If the data are **held or will be held in a public repository**, include URLs, accession numbers or DOIs. If this information will only be available after acceptance, indicate this by ticking the box below. For example: *All XXX files are available from the XXX database (accession number(s) XXX, XXX.*).<br>• If the data are all contained **within the manuscript and/or Supporting Information files**, enter the following: *All relevant data are within the manuscript and its Supporting Information files.*<br>• If neither of these applies but you are able to provide **details of access elsewhere**, with or without limitations, please do so. For example:<br><br>*Data cannot be shared publicly because of [XXX]. Data are available from the XXX Institutional Data Access / Ethics Committee (contact via XXX) for researchers who meet the criteria for access to confidential data.*<br><br>*The data underlying the results presented in the study are available from (include the name of the third party*	The data for this paper came from a bigger study funded by USAID's Agric Sector Policy Support Project (APSP) (Grant APSP-STS-012). The project was concluded, and the data, which was fully anonymised with no identifiable human subjects, has been archived according to APSP regulations.

and contact information or URL).<br>• This text is appropriate if the data are owned by a third party and authors do not have permission to share the data.<br><br>* typeset	
Additional data availability information:	

Manuscript
Click here to access/download;Manuscript;Improved version1.docx

Gendered dimensions of sustainable land management: evidence of farm size and training effects on willingness to pay in Ghana's Volta Region

Authors List

James Dickson Fiagborlo[1*], Mexoese Nyatuame[2] and Stella Ahone Ntoko[3]

[1] Department of Multidisciplinary Studies, Faculty of Applied Social Sciences, Ho Technical University, Ho, Ghana (jfiagborlo@htu.edu.gh (J.D. Fiagborlo)); ORCID: 0000-0002-3915-2939

[2] Department of Agric Engineering, *Faculty of Engineering*, Ho Technical University, Ho, Ghana (mnyatuame@htu.edu.gh (M. Nyatuame)); ORCID: 0000-0001-7652-3308

[3] Centre for Research in Efficient Agricultural Technologies (CREAT), Ho Technical University, Ho, Ghana (nstella15@gmail.com (S.A. Ntoko))

[*] Corresponding Author: James Dickson Fiagborlo, Department of Multidisciplinary Studies, Faculty of Applied Social Sciences, Ho Technical University, P.O. Box 217, Ho, Ghana. Tel**:** +233(0)200249032/+233(0)244047683 (jfiagborlo@htu.edu.gh)

Author Contribution

All authors contributed to the study conception and design. Material preparation, data collection and analysis were done by **James Dickson Fiagborlo**, **Mexoese Nyatuame** and **Stella Ahone Ntoko**. The initial draft of the manuscript was done by **James Dickson Fiagborlo** and all authors read the first versions of the manuscript. All authors approved the final manuscript.

Abstract

This study investigates gender differences in sustainable land management (SLM) training and farm size with respect to willingness to pay (WTP) for agricultural SLM practices in Ghana's Volta Region. Employing a Tobit regression model on primary data collected from five municipalities, the findings reveal that both SLM training and farm size have a statistically significant positive effect on WTP. Notably, these effects are further influenced by gender. While SLM training appears to reduce WTP among male farmers as farm size increases, female farmers—irrespective of training—demonstrated a higher WTP than their male counterparts with training. Additionally, the study finds that residing in coastal communities, having more dependants, lower income, and vocational education are negatively associated with WTP for SLM practices. These findings underscore the importance of tailoring SLM interventions to the gender-specific needs and conditions of farmers in the Volta Region.

Keywords: Gender differences. Farm size. SLM training. Tobit model. Volta Region. Willingness to pay

Introduction

Ghana's agricultural landscape is under severe threat, primarily due to deforestation, overgrazing, and uncontrolled burning. These anthropogenic activities contribute to the destruction of vegetation cover, triggering widespread soil erosion and salinisation. According to the Government of Ghana [1], approximately 69% of the country's agricultural land is already severely eroded. The implications are stark: land degradation exacerbates poverty [2], while soil deterioration costs the Ghanaian economy an estimated $4.2 billion in agricultural

output annually—equivalent to 5% of the agricultural sector's GDP [3]. This cycle of degradation and poverty undermines farmers' access to critical inputs such as fertiliser, irrigation, and mechanised tools.

Sustainable Land Management (SLM) and Agricultural Sustainable Land Management (ASLM) have emerged as pivotal approaches to mitigate these challenges. TerrAfrica defines SLM as "adopting land-use systems that enhance ecological support functions through appropriate management techniques". [4] extend this definition to encompass ASLM's role in improving the functional capacity of land for crop, livestock, watershed, and forest productivity. This paper uses the terms SLM and ASLM interchangeably.

SLM practices are increasingly recognised as vital for enhancing food security, reducing rural poverty, and promoting climate-resilient agricultural development [5]. Nevertheless, the uptake of these practices remains low, particularly among smallholder farmers in developing regions [6,7]. In Ghana's Volta Region, land degradation and climate variability continue to threaten agricultural sustainability [8]. Although awareness of SLM benefits exists, adoption is hindered by high implementation costs and limited access to requisite technologies [7]. Compounding this is the prolonged economic downturn and the unpredictable availability of donor funding, which further restrict the capacity of farmers to invest in sustainable practices [8]. A more nuanced understanding of the determinants of SLM adoption—mainly farmers' WTP for these practices—is critical to guiding the development of effective, inclusive strategies [9].

Prior studies [9-13] have identified socioeconomic factors, such as gender, age, education, and household composition, as influential in shaping WTP for environmental services. [9], for instance, highlight that gender, age group, farm size, and SLM training significantly affect farmers' WTP. However, the relationship between farm size and WTP remains ambiguous, and the influence of SLM training on WTP is underexplored. Moreover,

gendered dynamics in SLM adoption are often overlooked, despite evidence that women face disproportionate barriers in accessing land, credit, and agricultural extension services [9].

Unpacking the complex interplay between gender, SLM training, and farm size on WTP can inform targeted strategies to enhance the adoption of SLM practices. Therefore, this study explores gender dimensions in SLM training and farm size and how they influence WTP for SLM practices in the Volta Region. Precisely, the study aims to: (1) determine the effect of SLM training on WTP for SLM practices among male and female farmers; (2) examine the impact of farm size on WTP for SLM practices by gender; and (3) assess whether gender dimensions exist in WTP for SLM practices. Three hypotheses guide the empirical analysis: (1) farmers who have received SLM training will exhibit higher WTP than those without training; (2) larger farm size will be associated with higher WTP for SLM practices; (3) female farmers will demonstrate a higher WTP for SLM practices compared to their male counterparts.

This study is novel in many respects. First, it evaluates the combined and disaggregated effects of SLM training and farm size on farmers' WTP for SLM practices. Second, it highlights gender-specific differences in WTP, contributing to the literature on gender and sustainable agriculture. Third, it addresses a critical knowledge gap by focusing on the Volta Region—an area with limited empirical evidence on this subject. The significance of this study also lies in its potential to inform gender-sensitive and evidence-based policy interventions aimed at promoting SLM adoption. The results are equally relevant to current efforts to enhance climate-resilient agriculture and empower women in agricultural decision-making in Ghana.

The remainder of this paper is structured as follows: Section 2 presents a review of the literature, including SLM adoption and land policy frameworks in Ghana. Section 3 outlines the research methodology, including data sources and the econometric model. Section 4 reports and discusses the empirical results. Finally, Section 5 concludes with key policy recommendations and identifies limitations and areas for future research.

Literature review

This section provides an overview of land management strategies and policies in Ghana, followed by a discussion of empirical studies relevant to SLM, particularly in relation to gender, training, farm size, and WTP.

Land management techniques and policies in Ghana

Ghana's National Land Policy, introduced in 1999, was designed to establish efficient legal and administrative systems that protect land rights, recognise the authority of traditional institutions, and promote sustainable and productive land use amidst rising population and land-use pressures [14]. The policy seeks to address critical challenges within the land sector, such as widespread land market indiscipline, encroachments, multiple sales of land, and unplanned development [15]. Its objectives include safeguarding land tenure, enhancing productivity, and fostering sustainable land use [14].

To achieve these goals, the policy recommends several strategies: the formulation of district, regional, and national land-use plans; the development of comprehensive land-use atlases; dispute resolution through an inter-ministerial technical working group; and the inclusion of all recognised stakeholders in decision-making processes [14]. Notably, the policy also prohibits the clearance of primary forests or tree plantations for mining purposes. Effective implementation of these provisions is critical to ensuring equitable land access, reducing conflict, and achieving sustainable development outcomes [16].

Since the 1990s, Ghana has adopted various land management strategies aimed at enhancing agricultural land productivity and environmental sustainability [17]. These include key policy frameworks such as the Forest and Wildlife Policy, the Fire Policy, and the

Sustainable Land Management (SLM) Policy, all of which support the responsible use of natural resources within agriculture [1]. However, implementation challenges remain. For example, regulatory limitations have hindered the mainstreaming of SLM into broader agricultural strategies and action plans [1]. [18] identify several practical barriers to SLM adoption, including unreliable access to credit, weak market linkages, and high transaction costs for farm inputs and outputs. As a result, many farmers continue to rely on traditional, unsustainable practices such as slash-and-burn cultivation [19]. Moreover, a lack of standardised information on SLM requirements across Ghana's agro-ecological zones, combined with limited understanding of the economic trade-offs associated with agricultural land degradation and SLM adoption, undermines evidence-based policymaking [18].

Ghana's national development strategy recognises land degradation as a major impediment to economic growth. Despite this, the National Action Plan for combating desertification has yet to be fully integrated into development planning frameworks [20]. Conventional land-use practices continue to degrade the soil, contributing to an annual loss of approximately 2% of Ghana's Gross Domestic Product (GDP) [20]. In response, a number of programmes have been implemented, including the Food and Agriculture Sector Development Policy (FASDEP), the Northern Rural Growth Programme, and the ASLM Strategy, which led to the establishment of the Ghana Strategic Investment Fund (GSIF). The GSIF supports various SLM initiatives, such as agricultural asset financing and rural irrigation infrastructure. Despite these efforts, the effectiveness of existing SLM policies is often undermined by weak enforcement mechanisms, limited technical capacity, inadequate integration of ecosystem-based management approaches, and minimal engagement with local communities [20]. Besides, limited research has explored joint, multi-stakeholder approaches that involve farmers, extension agents, scientists, and policymakers [8], suggesting a need for more inclusive and participatory SLM governance.

Empirical literature

Empirical studies confirm that SLM practices are vital for maintaining soil fertility, conserving water, and supporting biodiversity [5]. Techniques such as conservation agriculture, agroforestry, and integrated soil fertility management have demonstrated positive impacts on crop yields, soil stability, and climate resilience [9]. Nonetheless, adoption rates remain low, particularly among smallholder farmers in developing countries, due to limited access to finance, inadequate extension services, and insufficient policy support [6,7]. While larger farms often possess the resources required to adopt SLM practices [6], smallholder farmers can also benefit—especially when supported by targeted training and institutional frameworks [9]. Training in SLM methods has been found to significantly improve knowledge and the uptake of conservation techniques, such as reduced tillage, crop rotation, and organic soil amendments [6]. Importantly, SLM adoption is not gender-neutral. Women, particularly in patriarchal rural settings, often face systemic barriers to accessing resources and training [6,7]. However, when provided with adequate support, women have shown a strong capacity to adopt and benefit from sustainable practices.

Although the literature on farmers' WTP for SLM is still emerging, WTP is increasingly recognised as a critical cause of adoption, reflecting farmers' perceived utility and valuation of long-term land improvements [6,21]. Research suggests that when farmers expect tangible returns—such as increased yields or reduced input costs—they are more likely to invest in SLM measures [10]. Several studies have investigated the socioeconomic causes of WTP. For example, [22] assessed graduate farmers' WTP for irrigated plots in Egypt's desert reclamation programme. [13] found that education, farm size, and household income significantly influenced WTP for irrigation water in Tanzania's Lower Moshi and Pangani Basin, while [23] reported no significant gender or education effects on WTP in Northwest

China. In Nigeria, [24] established that age, income, education, and household size positively influenced WTP for irrigation at the Oshin project site. More recently, [21] highlighted that demographic characteristics, asset ownership, institutional services, and bid price variables were the main predictors of household WTP for sustainable land use.

Although these studies provide useful insights, several gaps persist in the literature. First, there is limited understanding of the direct impact of SLM training on farmers' WTP for SLM practices. Second, the relationship between farm size and WTP remains ambiguous and underexplored. Third, there is insufficient research examining the intersection of gender, SLM training, and farm size in shaping WTP in the context of Ghana's Volta Region. This study addresses these research gaps by exploring how gender moderates the effects of SLM training and farm size on farmers' WTP for SLM practices. Understanding these dynamics is vital for informing gender-sensitive policy interventions and designing effective extension services and development strategies that enhance sustainable land use among farmers in the Volta Region.

Materials and methods

To investigate the gendered dimensions of SLM and evidence of farm size and training effects on WTP in Ghana's Volta Region, this study employed a primary data collection approach. A multistage sampling technique was utilised, involving the selection of 25 communities across five municipalities within the region. In each community, 50 farm households were randomly selected using simple random sampling, resulting in a total sample of 1,250 households. Following [25], this sample size was deemed adequate, both for meeting the study's objectives and due to the relative homogeneity of the target population. While the heads of farming households were the primary respondents, any adult household member aged 18 years or older was eligible for interview in the absence of the head. Data were collected via face-to-face interviews using a structured questionnaire administered in English and Ewe.

Given the study's focus, the questionnaire captured detailed sociodemographic information, including the respondent's age, gender, and educational attainment. Other variables included farming experience, monthly income, household size (number of dependants), farm size, place of residence (coastal or inland), and prior training in ASLM practices. WTP was elicited using a contingent valuation question framed as follows: "ASLM practices serve multiple functions: they prevent soil erosion, ensure water retention, support groundwater recharge, and preserve rural landscapes. They also help mitigate flooding. In view of these benefits, what is the maximum amount in Ghanaian Cedis (GHS) that you are willing to pay to sustain the environmental benefits provided by ASLM practices?" The fieldwork was conducted between October and November 2016. Data, which was fully anonymised with no identifiable human subjects, were analysed using Stata version 15.0, and the findings are presented using descriptive statistics and Tobit regression results.

Measurement of variables

This section defines and outlines the measurement of key variables included in the empirical analysis. The dependent variable, WTP, represents the maximum amount a respondent is willing to pay for improved SLM services. It is a non-negative, censored variable: zero indicates unwillingness to pay, while positive values indicate the maximum WTP. Income is a continuous variable measured in Ghanaian Cedis (GHS), reflecting monthly household earnings. In line with economic theory, income is expected to have a positive effect on WTP, as SLM is assumed to be a normal good. Household dependants represent the number of individuals reliant on the household's income. This is a non-negative integer variable. A negative link is expected; as higher dependency ratios may reduce disposable income available for environmental investments. Farming experience is measured as the number of years of farming activity. It is expected to have a negative effect on WTP, based on the assumption that

more experienced farmers may be less receptive to adopting new practices. Farm size is a continuous, positive variable (in acres or hectares) hypothesised to be positively associated with WTP, given that larger landholdings may offer greater scope for SLM investment returns.

Gender, SLM training, and coastal communities were dummied. While gender takes 1 if the respondent is male and 0 if the respondent is female, SLM training takes 1 if the respondent has some SLM training and 0 otherwise. Likewise, coastal communities take 1 if the farmer resides in a coastal area and 0 otherwise. While gender and coastal communities are expected to have negative effects on WTP, SLM training is predicted to have positive effects on WTP. Following [26], five dummy variables were created based on the six-way category of the level of education: category six (1) vs. category one (0); category five (1) vs. category one (0); category four (1) vs. category one (0); category three (1) vs. category one (0); and category two (1) vs. category one (0). This dummies allowed for the capturing of the educational differences in WTP for SLM practices in the [27] model.

Econometric model

The empirical analysis is grounded in the Random Utility Maximisation (RUM) theory proposed by [28], which posits that individuals make utility-maximising choices based on available alternatives, preferences, and constraints [29,30]. Under this framework, individuals are assumed to act rationally, selecting options that yield the highest expected utility. According to recent papers [31-34], the utility experienced by a person (n) for choice (j) can be represented as a linear function:

$$V_{jn} = \beta_{0j} + \beta_{1j} x_{1n} + \beta_{2j} x_{2n} + \beta_{3j} x_{3n} + \ldots + \beta_{nj} x_{qn} \tag{1}$$

Where β_{0j} is the alternative specific constant for choice j, $\beta_{1j}, \beta_{2j}, \ldots, \beta_{nj}$ indicates the n coefficients associated with the predictors, $X_{1n}, X_{2n}, \ldots, X_{qn}$ indicates the explanatory factors unique to individual n, and q is the total number of predictive variables in the model.

The dependent variable (WTP) is censored, as it includes zero values for non-payers and positive values for payers. Given this characteristic, [27] censored regression model (the Tobit model) is appropriate. Following [35,36], the Tobit model is specified as:

$$\begin{aligned} &WTP^*_i = X_i\beta + \varepsilon_i \\ &\mathrm{WTP}_i = \mathrm{WTP}^*_i, \ \text{if } X_i\beta + \varepsilon_i > 0 \\ &WTP_i = 0, \ \text{if } X_i\beta + \varepsilon_i \leq 0 \end{aligned} \quad (2)$$

where WTP^*_i is the latent (unobserved) variable representing the maximum amount a farmer is willing to pay, X_i denotes the set of explanatory variables, β are as before in (1), and ε_i denotes the stochastic term, which is considered to be independent and normally distributed with a zero mean and constant variance. From (2), when the WTP_i is negative (i.e., less than zero), it goes unnoticed. However, when WTP_i is beyond the censoring limit (i.e., greeter than zero), it is noticed. The expected WTP_i for a random observation is:

$$E(WTP_i) = \Phi\left(X_i\beta/\sigma\right)(X_i\beta + \sigma\lambda_i); \quad \lambda_i = \phi\left(X_i\beta/\sigma\right)/\Phi\left(X_i\beta/\sigma\right) \quad (3)$$

In equation (3), Φ denotes the normal distribution function, λ the normal density function, σ the standard deviation, $\phi(\bullet)$ the standard normal probability density function, and $\Phi(\bullet)$ the normal cumulative density function. [37] discovered that the expected value of WTP for observations above zero, denoted by $E(WTP^*)$, is the expected value of the truncated normal error component $X\beta$ plus its expected value. This is represented as follows:

$$E(WTP_i) = \Phi\left(X_i\beta/\sigma\right)E(WTP^*) \quad (4)$$

Unlike a linear model, the marginal effect (ME) of predictors in Tobit estimation is nonlinear and unequal. [38] define the ME in Tobit regression as follows:

$$\frac{\partial E(WTP_i)}{\partial X_i} = \Phi\left(\frac{X_i\beta}{\sigma}\right)\left(\frac{\partial E(WTP^*)}{\partial X_i}\right) + E(WTP^*)\left(\frac{\partial \Phi\left(\frac{X_i\beta}{\sigma}\right)}{\partial X_i}\right) \quad (5)$$

From (2), the functional form of the empirical model is specified as:

$$WTP_i = \beta_0 + \beta_1 slmt_i + \beta_2 fams_i + \beta_3 gm_i + \beta_4(\text{slmt}_i \text{ x fams}_i) + \beta_5(\text{slmt}_i \text{ x gm}_i) + \beta_6(\text{fams}_i \text{ x gm}_i) + \beta_7(\text{slmt}_i \text{ x gm}_i \text{ x fams}_i) + \beta_8 coc_i + \beta_9 linc_i + \beta_{10} hd_i + \beta_{11} fe_i + \beta_{12} ed_i + \varepsilon_i \quad (6)$$

The WTP_i represents the probability of a farmer WTP the maximum option i. Equation (6) consists of independent variables such as sustainable land management training (slmt), farm size (fams), gender (gm), coastal areas (coc), income (inc), household dependant (hd), farmer experience (fe), and education (ed). Equation (6) similarly includes interaction terms (slmt x fams, slmt x gm, gm x fams, and slmt x gm x fams) to estimate the intricate nexuses between sociodemographic factors (sustainable land management training, farm size, and gender) and WTP for SLM. The model was estimated using the Maximum Likelihood Estimation (MLE) technique. Following [39], average marginal effects of the interaction terms were computed to examine the combined influence of the factors on WTP for SLM practices.

Results and discussions

Table 1 summarises the descriptive statistics of the variables considered. The farmers' WTP for SLM practices was GHS 76.59 (US$ 19.15)[1]. We estimated the total WTP by multiplying the mean WTP by the total number of agricultural households, given that the mean WTP encompasses all agricultural households in the Volta region and Ghana. The 2010 population and housing census showed that 888,271 people in the Volta region worked in the agricultural sector. Based on an average household size of 4.4, we estimated the number of agricultural households to be 201,879.77. With a national agricultural population of 10,479,485, Ghana's agricultural households was estimated to be 2,381,701. Multiplying this data by the mean WTP

resulted into GHS 15,461,971.58 (US$ 3,865,492.90) for the region and GHS 182,414,479.59 (US$ 45,603,619.90) for Ghana, respectively.

Table 1. Summary statistics

Variables	Observation	Mean	SD	Min	Max
WTP	1036	76.59	118.24	0	500
SLM training	1036	0.35	0.48	0	1
Farm size	1036	4.00	3.57	1	45
male	1036	0.60	0.49	0	1
coastal	1036	0.54	0.50	0	1
Income	1036	346.62	207.62	30	1000
Household dependant	1036	4.96	2.40	1	18
Farming experience	1036	11.47	10.05	1	60
No education	1036	0.10	0.30	0	1
Primary education	1036	0.19	0.39	0	1
Middle school education	1036	0.52	0.50	0	1
Secondary education	1036	0.10	0.30	0	1
Vocational education	1036	0.05	0.21	0	1
Tertiary education	1036	0.04	0.19	0	1

NB: SD means standard deviation

[1]*Exchange rate is US$1= GHS4—the average daily exchange at the time of data collection (October 2016 to November 2016). This was retrieved from the Bank of Ghana at https:// www. bog. gov.gh/ treasury- and- the- markets/ historical-interbank- fx- rates/*

The average WTP [GHS 76.59 (US$ 19.15)] represents about 19% of the average monthly income that farmers are willing to pay for ASLM. Based on this figure, farmers may be willing to pay 19% of their average monthly farm income as a premium for agricultural insurance. The statistics show that 35% of the farmers had SLM training, while the majority

(54%) lived in coastal areas. While the average income was GHS 346.62 (US$ 86.66), Table 1 reveals that male farmers were 60% compared with female farmers. The average number of dependants was 5 people, while the farmers experience ranged between 1 to 60 years. Only 10% of the farmers lacked education, with 90% having education up to tertiary education. Table 1 reveals the average farm size to be 4 acres.

Farmers' reluctance to pay for SLM practices can be attributed to various reasons. Fig 1 shows that about 177 (31%) farmers said the government should provide funds for SLM practices. Eighty-nine (16%) farmers felt that their high farm expenditures were already overwhelming them. Additionally, about 84 (15%) of the farmers argued that they would only pay if they saw improvements in ASLM practices. Meanwhile, about 49 (9%) of the farmers believed that government employees were ineffective and that farmers' money might end up in their pockets. Other 25 farmers (4%), would only pay without SLM activities. Farmers' varied reasons suggest that policymakers need to understand their genuine fears about public officials and the need to support ASLM activities before encouraging them to pay for ASLM services.

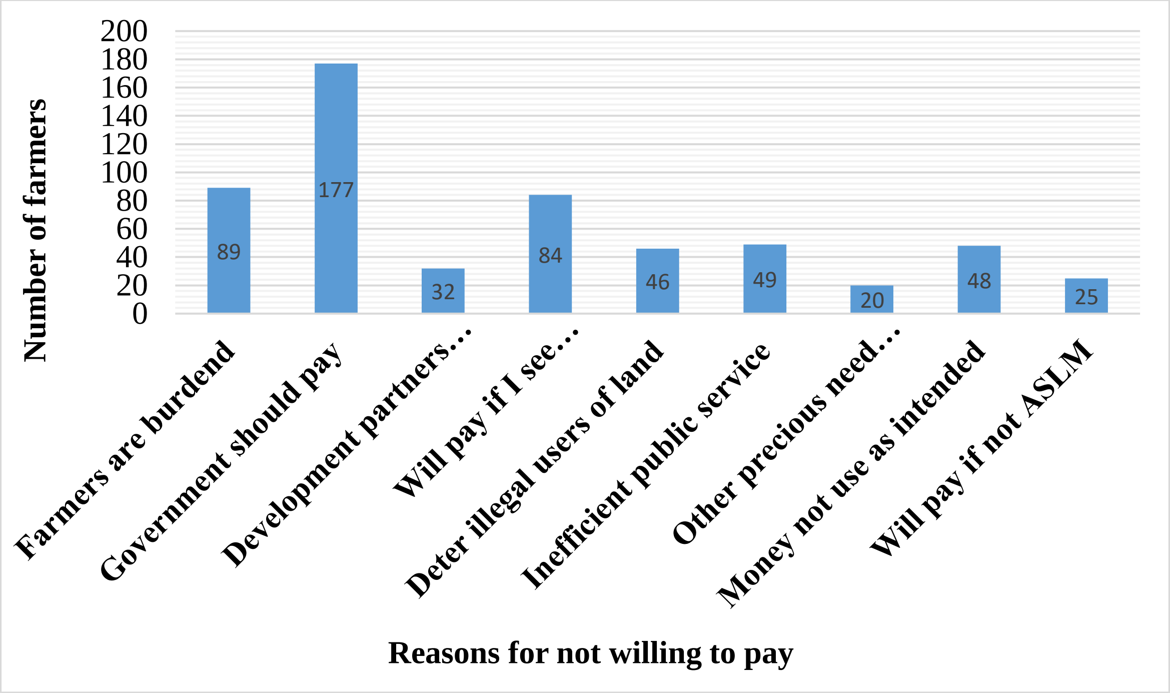

Fig 1: Reasons for not willing to pay for ASLM service

State officials must earn farmers' trust in the management of public funds. Given the literature, low trust and confidence in public institutions may prevent "ordinary" people from taking on greater responsibilities to enhance public services [40]. A little over 15% of those surveyed said they would only pay if ASLM services were better. According to the Centre for Environment and Development (CED), unreliable service that is late or falls short of expectations hurts people's confidence and willingness to pay. A review of the literature reveals that public institutions or affiliated organisations that collect and handle public funds significantly influence people's willingness to pay [41]. People are less likely to object to paying more for public services if they have confidence in the intended use of their financial contributions. On the other hand, if these concerns remain unaddressed, individuals or organisations responsible for financial administration will perform effectively, but advocating for higher public service fees will prove challenging.

Empirical results

This study explores gender differences in SLM training and farm size on WTP for SLM practices in the Volta region. Table 2 covers the average marginal effects and summary statistics of the model. The Likelihood Ratio Chi-Square (LR chi^2) test uses the log likelihood statistics (-993.93281) from Table 2 to determine the statistical stability of the model [42,43]. The Tobit model is statistically significant, as the LR-chi^2 value of 108.85 in Table 2 relates to a small p-value of 0.000. As the model's pseudo-R^2 shows, 6.3% of the change in farmers' WTP for SLM was due to the predictive variables. However, this doesn't mean anything statistically because the pseudo-R^2 formula can give results > 1 or < 0 for discrete likelihoods like Tobit. [44] maintained that where $0 \leq$ pseudo-$R^2 \leq 1$, it is better to interpret the model chi^2 and the p-value than pseudo R^2. Table 2 shows the average marginal effects and their standard errors in

parentheses. The average marginal effects show changes in probability linked with a one-unit change in the predictors.

Table 2: Average marginal effects

Variables	Tobit model
Sustainable land management training (SLM training)	0.1110 (0.0275)***
Log of farm size	0.0668 (0.0194)***
Male	0.0061 (0.0282)
SLM training x log of farm size	0.2481 (0.0582)***
SLM training x Male	0.1772 (0.0707)**
Male x log of farm size	0.0485 (0.0490)
SLM training x Male x log of farm size	0.2629 (0.0783)***
Coastal	-0.1960 (0.0264)***
Log of income	0.0374 (0.0194)*
Log of farmer experience	-0.0061 (0.0160)
Household dependant	-0.0732 (0.0241)***
Primary education	0.00568 (0.0508)
Middle school education	0.0326 (0.0453)
Secondary education	-0.0866 (0.0598)
Vocational education	-0.2150 (0.0772)***
Tertiary education	-0.0491 (0.0792)
Observations	1,036
Log likelihood	-993.93281
LR chi^2	108.85
Pseudo R^2	0.0626
Prob> F	0.0000

Note: Standard errors in parentheses *** p<0.01, ** p<0.05, * p<0.1

Table 2 shows that SLM training statistically explains WTP for SLM practices at a 1% level of significance. Specifically, the marginal effect of SLM training on WTP is 0.111, implying that farmers with SLM training are WTP an extra 0.111 currency units for SLM practices. The knowledge gained through training may increase farmers' confidence and trust in policymakers. As such, farm households that got SLM training were more inclined to pay for SLM initiatives than those without. This contradicts [45], who found that farmers with access to agricultural service data were less likely to pay for them.

Additionally, Table 2 shows that the size of farmers' farms has a significant impact on their WTP for SLM activities. In particular, the marginal effect of farm size on WTP is 0.067, indicating that an extra acre of farm size increases farmers' WTP by 0.067 currency units for SLM practices. This finding is plausible because larger farms can benefit from economies of scale in implementing SLM practices. For instance, with an additional acre of land, farmers can spread the fixed costs of adopting sustainable practices over a larger area, making it more cost-effective. Moreover, farmers with large land may be more likely to think about the long-term sustainability of their farm and the potential benefits of adopting sustainable land management practices. For example, with a longer-term perspective, farmers may be more willing to invest in practices that may not provide immediate benefits but will pay off in the long run, which can increase their WTP for SLM practices. While this supports the finding by [24], who found a positive link between WTP for irrigation facilities, it contradicts the result by [7], who found a negative link between WTP for SLM practices and the size of farmland.

Furthermore, Table 2 shows that the coastal zone has a negative influence on WTP for SLM practices. Specifically, the marginal effect of being in a coastal zone on WTP is -0.196, signifying that farmers in coastal communities are less willing to pay 0.196 currency units for SLM practices than northern community farmers. Farmers in the coastal communities were expected to have a lower preference for ASLM issues than those in the northern communities,

because many of them may have fishing as an alternative source of income and will be unwilling to make monetary contributions to improve and enhance the land's caring capacity. Evidence suggests that the coastal community farmers had benefitted from the MiDA project on SLM practices and could have just been adopting them. Therefore, it is sound to conclude that this is what has negatively influenced their men's decision to pay for ASLM practices.

Table 2 shows that the log of household income is positive and statistically significant at 10%. Apparently, the marginal effect of the log of income on WTP is 0.0374, meaning that for every percentage increase in income, farmers will be WTP an additional 0.0374 currency units for SLM practices. Income determines people's ability to pay. Therefore, farmers with higher income may be more willing to take on the risks linked with adopting new practices, which can surge their WTP for SLM practices. Besides, with a higher income, farmers may be more willing to invest in expensive SLM practices. This finding reflects those by [13,46-51].

The result in Table 2, however, shows an insignificant negative correlation between the log of farmer experience and WTP for ASLM practices. Indeed, the marginal effect of farmer experience on WTP is -0.0061, suggesting that farmers with extra years of farming experience have a lower WTP of an additional 0.0061 currency units for SLM practices. This result is reasonable because experienced farmers may have indigenous knowledge of land management and may not be willing to pay for improved ASLM services. This means that any policy intervention that seeks to encourage SLM should focus on the inexperienced farmers rather than experienced ones. While this finding supports the negative effect of farmer experience on WTP [52,53], it contradicts the results by [7,54], who found that the number of years spent farming had a significant positive influence on WTP.

Similarly, the coefficient for the dependant households shows a negative statistically significant value at 1%. Specifically, Table 2 shows that the marginal effect of household dependants on WTP is -0.0732, implying that farmers with an additional dependant have a

lower WTP of 0.0732 currency units for SLM practices. This result is tenable, as larger dependent households come with the added expense of meeting their needs. Thus, paying for ASLM services may not be a rational choice. While this result contradicts the findings by [46,50,55], it mirrors the findings by [24,56,57], who found adverse effects of family size on WTP for SLM practices. This result also supports theory of basic human needs by [58,59,60].

The model also takes into account the household head's highest level of education. The vocational education dummy is statistically significant at 5% and exhibits a negative sign. The result suggests that farm household heads with vocational training are less likely to pay for ASLM services than those with no formal education. In particular, farmers with vocational training have a lower WTP of 0.2150 currency units for SLM practices compared to uneducated farmers. White-collar jobs are more likely to be available to people with a higher level training, and people with those jobs are more likely to work outside of farming. As a result, they may be less concerned about ASLM issues than those with no formal education. The finding contradicts prior research [13,61,62], which found that the educated were more WTP for improved services than the uneducated.

Average marginal effects of the interaction terms

The result in Table 2 shows that an insignificant positive marginal effect of gender on WTP for SLM practices is 0.00605, signifying that male farmers have a higher WTP for SLM practices of 0.0061 currency units than female farmers. However, to appreciate the moderating effect of gender on the link between SLM training and WTP for SLM practices, this study included an interaction between SLM training and gender in the model. Table 2 shows that the marginal effects of the two-way interaction between SLM training and gender on WTP is 0.1772, implying that the effect of SLM training on WTP for SLM practices is 0.1772 currency units higher for male farmers than for female farmers. Several factors, including gender roles and

responsibilities, financial resources, and risk perception and tolerance, may have accounted for this finding. For instance, in many societies, people often perceive men as the main decision-makers and land managers, whereas women may have limited control over land and resources. Thus, men may have higher WTP for SLM practices. Besides, men may perceive the risks related to SLM practices as lower than women, which can surge their WTP for these practices.

Table 2 further shows that the marginal effect of the interaction between SLM training and farm size on WTP is 0.2481, suggesting that the effect of SLM training on willingness to pay increases by 0.2481 currency units for every additional acre of farmland. Illustratively, larger farms may be more likely to invest in SLM practices, as they have more resources and a greater stake in the long-term productivity of their land. Thus, as farm size increases, the likelihood of adopting SLM practices may also increase, leading to a higher willingness to pay. In addition, larger farms may benefit more from SLM practices, such as reduced soil erosion, improved soil fertility, and increased crop yields. Consequently, as farm size increases, the benefits of SLM practices may also increase, leading to a higher willingness to pay.

This study also gauges the two-way interface effects of gender and farm size on WTP for SLM practices. Table 2 reveals that the marginal effect of the interface between gender and farm size on WTP for SLM practices is 0.0485, showing that the effect of farm size on WTP for SLM practices is enhanced by 0.0485 currency units for male farmers than for female farmers. Male farmers may experience greater effects of farm size on WTP for SLM practices compared to their female counterparts for several reasons: First, men may be more likely to own land or have secure tenure rights, which can increase their WTP for SLM practices as farm size increases. Second, larger farms may benefit from economies of scale in implementing SLM practices, making it more cost-effective for male farmers to invest in them. Third, men may have greater confidence and self-efficacy in their ability to implement SLM practices, which can increase their WTP for these practices as farm size increases.

To understand the nuanced effects of SLM training, gender, and farm size on WTP for SLM practices, this study included a three-way interaction term in the model. Table 2 demonstrates that the interaction between SLM training, gender, and farm size has a marginal effect of 0.2629 on WTP for SLM practices. This suggests that SLM training enhances WTP for SLM practices by 0.2629 currency units per additional acre of farmland, but only for male farmers compared to female farmers. However, Fig 2, which further illustrates the gender differences in the effects of SLM training on WTP for SLM practices as farm size increases, reveals a decline in WTP for SLM practices for male farmers with SLM training, while it increases for those without SLM training. The decline in WTP for SLM practices for male farmers with SLM training as the farm size increases may be due to economies of scale. For

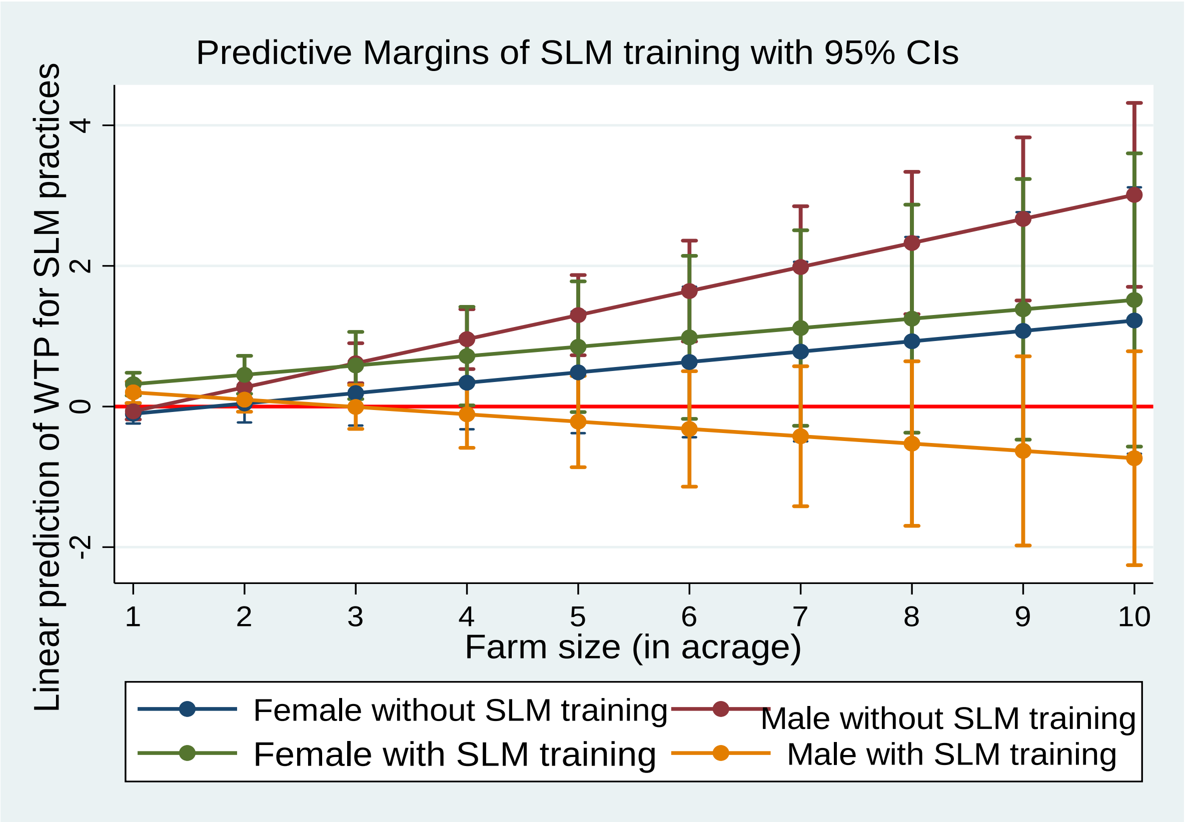

Fig 2: Average marginal effect of the interaction between SLM training, gender, and farm size on willingness to pay for sustainable land management practices

instance, as the size of the farm rises, male farmers with SLM training may feel that they can achieve economies of scale by implementing SLM practices on their own, without needing to invest any extra resources or support. Moreover, male farmers with SLM training may become more confident in their ability to manage their land sustainably as the size of their farm rises. This increased confidence could lead them to be less willing to pay for SLM practices.

Furthermore, as the size of the farm increases, male farmers with SLM training may perceive that the marginal benefits of investing in SLM practices decrease. This could lead them to be less willing to pay for SLM practices. Similarly, as the size of the farm increases, male farmers with SLM training may face increasing opportunity costs associated with investing in SLM practices. For instance, they may need to divert resources away from other farm activities in order to invest in SLM practices. Likewise, male farmers with SLM training may employ risk management strategies such as diversification, which could reduce their willingness to pay for SLM practices as the size of their farm increases. It is also possible that male farmers with SLM training may be influenced by socio-cultural factors such as traditional farming practices, social norms, and community expectations. As the size of the farm increases, these factors may become more influential, leading to less WTP for SLM practices. Another factor that could prevent male farmers with SLM training from adopting SLM practices is that the SLM training program may not have sufficiently addressed the needs and concerns of male farmers with larger farms. As a result, they may not see the value in investing in SLM practices.

For female farmers, regardless of SLM training, Fig 2 reveals that the WTP for SLM practices rises as the size of the farmland increases. For instance, with an acre of land, female farmers without SLM training have negative WTP for SLM practices compared to those with SLM training. However, as the farm size increases to 10 acres, the effects on WTP for SLM practices for female farmers, regardless of SLM training, are positive and noticeably high. The findings further suggest that while male farmers with SLM training are less WTP for SLM

practices as their farm size increases, female farmers with or without SLM training are highly WTP for SLM practices, just like the male farmers without SLM training.

Conclusions

This study examined gender differences in SLM training and farm size on WTP for SLM practices by applying a Tobit regression model to primary data from five municipalities in the Volta region of Ghana. The results revealed that SLM training and farm size significantly positively impacted WTP for SLM practices, with gender and farm size further amplifying these effects. Especially, the results show that while SLM training has a negative impact on WTP for SLM practices for male farmers as farm size rises, female farmers, regardless of SLM training, were more positive in their WTP for SLM practices relative to male farmers without SLM training. This finding implied that male farmers with bigger farms and SLM training were less WTP for SLM practices, while female farmers with smaller farms and SLM training were more WTP. Finally, this study has shown that the coastal community, income, family dependants, and vocational training have negative effects on WTP for SLM practices.

This study suggests tailoring SLM practices to meet the specific needs of male and female farmers, and supporting their adoption through policies. Future research should integrate insights from behavioural economics and psychology to address the often excluded female farmers from decision-making processes. Practical effects include training programs emphasising the economic benefits of SLM practices, providing incentives and support for adoption, and promoting policies that promote SLM adoption, mainly among female farmers. Monitoring and evaluating SLM programs is also crucial for their effectiveness and efficiency.

This study's use of data from only five municipalities in the Volta region presents some limitations. The Volta region, with its unique geography, climate, and cultural practices, has unique socioeconomic and cultural factors linking farmers' WTP for SML practices. This

study's findings may not be generalisable to other regions due to these variables. However, it provides valuable basis for further research, suggesting the use of national or longitudinal data for analysing SML issues, as this study may not have controlled for all confounding factors.

Acknowledgements

The data for this paper came from a bigger study funded by USAID's *Agric Sector Policy Support Project* (APSP) (Grant APSP-STS-012). The project was concluded, and the data, which was fully anonymised with no identifiable human subjects, has been archived according to APSP regulations. We thank our most valued respondents for their insights and time.

References

1. Government of Ghana. Ministry of Local Government and Rural Development [Internet]. Ghana; 2016. Available from: http://www.ghanadistricts.com
2. Diao X, Sarpong DB. Poverty implications of agricultural land degradation in Ghana: an economy-wide, multimarket model assessment. African Development Review. 2011 Sep;23(3):263-75.
3. Jinbaani AN. Sustainable land management in the Upper West region of Ghana: a forgotten solution to land degradation, communication failure or a non-priority option? [final project]. Reykjavik: United Nations University Land Restoration Training Programme; 2016 [cited 2022 Nov 4]. Available from: http://www.unulrt.is/static/fellows/document/jinbaani2016.pdf
4. Matlou R, Bahta YT, Owusu-Sekyere E, Jordaan H. Impact of agricultural drought resilience on the welfare of smallholder livestock farming households in the Northern Cape Province of South Africa. Land. 2021 May 27;10(6):562.

5. McElwee P, Calvin K, Campbell D, Cherubini F, Grassi G, Korotkov V, Le Hoang A, Lwasa S, Nkem J, Nkonya E, Saigusa N. The impact of interventions in the global land and agri-food sectors on Nature's Contributions to People and the UN Sustainable Development Goals. Global Change Biology. 2020 Sep;26(9):4691-721.
6. Legesse W, Haji J, Ketema M, Emana B. Determinants of adoption of sustainable land management practice choices among smallholder farmers in Abay Basin of Oromia, Ethiopia. Journal of Development and Agricultural Economics. 2021 Mar;13(1):1-9.
7. Takele MT, Umer MI. Determinant Factors of Farmer's Willingness to Pay for Sustainable Land Management Practices Evidence with Homosha Benishangul Gumuz Region: Application with Multinomial Logit Model, Mixed Logit Model, and Conditional Logit Model.
8. Ali EB, Agyekum EB, Adadi P. Agriculture for sustainable development: A SWOT-AHP assessment of Ghana's planting for food and jobs initiative. Sustainability. 2021 Jan 11;13(2):628.
9. Alemu GT, Tsunekawa A, Haregeweyn N, Nigussie Z, Tsubo M, Elias A, Ayalew Z, Berihun D, Adgo E, Meshesha DT, Molla D. Smallholder farmers' willingness to pay for sustainable land management practices in the Upper Blue Nile basin, Ethiopia. Environment, Development and Sustainability. 2021 Apr;23(4):5640-65.
10. Amoah A, Jumah A, Korle K. Exploring willingness-to-pay for the restoration and maintenance of reserved forests in the Greater Accra Region of Ghana. Forest Ecosystems. 2022 Jan 1; 9:100041.
11. Amoah A, Moffatt PG. Willingness to pay for reliable piped water services: evidence from urban Ghana. Environmental Economics and Policy Studies. 2021 Oct;23(4):805-29.

12. Rapsomanikis G. The economic lives of smallholder farmers: an analysis based on household data from nine countries. Rome: Food and Agriculture Organization of the United Nations; 2015.
13. Ndetewio PI, Mwakaje AG, Mujwahuzi M, Ngana J. Factors influencing willingness to pay for watershed services in lower Moshi, Pangani Basin, Tanzania.
14. Akaateba MA. The politics of customary land rights transformation in peri-urban Ghana: Powers of exclusion in the era of land commodification. Land Use Policy. 2019 Nov 1; 88:104197.
15. Bonye SZ, Yiridomoh GY, Bebelleh FD. Compliance with land use regulations in peri-urban areas in Ghana: a study of Bamahu and Danko residential areas in Wa municipality, Upper West Region. GeoJournal. 2021 Dec;86(6):2845-59.
16. Ayano MF. Understanding the Local Complexities in Land Law Reforms: The Case of Land Inalienability in Ethiopia, 1991–2018. Law & Social Inquiry. 2024 Nov;49(4):2398-438.
17. Kyei-Mensah C, Kyerematen R, Adu-Acheampong S. Impact of rainfall variability on crop production within the Worobong Ecological Area of Fanteakwa District, Ghana. Advances in Agriculture. 2019;2019(1):7930127.
18. Asamoah M, Owusu-Ansah F. Report on land tenure and cocoa production in Ghana. Cocoa Research Institute of Ghana and World Cocoa Foundation: Accra, Ghana. 2017 Feb.
19. Lambongang M, Ansah IG, Donkoh SA. Participation and yield effect of Ghana's planting for food and jobs programme in Bunkpurugu-Yunyoo district. Ghana Journal of Agricultural Economics and Agribusiness Vol. 2019 Jun;2(1).

20. Kidido JK, Bugri JT. Youth access to agricultural land from peri-urban and rural perspective: a study of Techiman traditional area in Ghana. Journal of Land and Rural Studies. 2020 Jan;8(1):37-56.
21. Abdeta D. Households' willingness to pay for forest conservation in Ethiopia: A review. Journal of Forest Science. 2022;68(11):437.
22. Shepler R, Suter JF, Manning DT, Goemans C. Private Actions and Preferences for Coordinated Groundwater Conservation in Colorado's Republican River Basin. JAWRA Journal of the American Water Resources Association. 2019 Jun;55(3):657-69.
23. Tang Z, Nan Z, Liu J. The willingness to pay for irrigation water: A case study in Northwest China. Global Nest Journal. 2013 Mar 1;15(1):76-84.
24. Fakayode SB, Ogunlade I, Ayinde O, Olabode P. Factors affecting farmers' ability to pay for irrigation facilities in Nigeria: the case of Oshin Irrigation Scheme in Kwara State. Journal of sustainable development in Africa. 2010 Dec 1;12(1):334-50.
25. Furlong M. 'Clear at a distance, jumbled up close': observation, immersion and reflection in the process that is creative research.
26. Borders SB. *Transportation barriers to health care: Assessing the Texas Medicaid program* (Doctoral dissertation, Texas A&M University).
27. Tobin J. Estimation of relationships for limited dependent variables. Econometrica: journal of the Econometric Society. 1958 Jan 1:24-36.
28. McFadden D. Econometric models for probabilistic choice among products. Journal of Business. 1980 Jul 1: S13-29.
29. Pinjari AR, Bhat C. Computationally efficient forecasting procedures for Kuhn-Tucker consumer demand model systems: Application to residential energy consumption analysis. Journal of choice modelling. 2021 Jun 1; 39:100283.

30. Zhao Z, Dua D, Singh S. Generating natural adversarial examples. arXiv preprint arXiv:1710.11342. 2017 Oct 31.
31. Fiagborlo JD, Vondolia GK, Obeng CK. Effects of travel time on mode choice for different locations and genders in Ghana. SN Social Sciences. 2024 Sep 13;4(9):172.
32. Payne SJ, Howes A. Adaptive interaction: A utility maximization approach to understanding human interaction with technology. Springer Nature; 2022 Jun 1.
33. Niromandfam A, Yazdankhah AS, Kazemzadeh R. Modeling demand response based on utility function considering wind profit maximization in the day-ahead market. Journal of Cleaner Production. 2020 Apr 1; 251:119317.
34. Fan A, Grave E, Joulin A. Reducing transformer depth on demand with structured dropout. arXiv preprint arXiv:1909.11556. 2019 Sep 25.
35. Loomis JB. Balancing public trust resources of Mono Lake and Los Angeles' water right: An economic approach. Water Resources Research. 1987 Aug;23(8):1449-56.
36. Cameron AC, Trivedi PK, Cameron AC, Trivedi PK. Microeconometrics using stata. College Station, TX: Stata press; 2010 Jan 1.
37. Amemiya T. Regression analysis when the dependent variable is truncated normal. Econometrica: Journal of the Econometric Society. 1973 Nov 1:997-1016.
38. McDonald JF, Moffitt RA. The uses of Tobit analysis. The review of economics and statistics. 1980 May 1:318-21.
39. Long JS, Freese J. Predicted probabilities for count models. The Stata Journal. 2001 Nov;1(1):51-7.
40. Asante BK. The economy is not healthy: whose fault? Daily Graphic. 2013;(19214):7.
41. Whittington D. Ethical issues with contingent valuation surveys in developing countries: A note on informed consent and other concerns. Environmental and Resource Economics. 2004 Aug;28(4):507-15.

42. Hansen BB, Pedersen ÅØ, Peeters B, Le Moullec M, Albon SD, Herfindal I, Sæther BE, Grøtan V, Aanes R. Spatial heterogeneity in climate change effects decouples the long-term dynamics of wild reindeer populations in the high Arctic. Global Change Biology. 2019 Nov;25(11):3656-68.
43. Malone HE, Coyne I. Complementing the P-value from null-hypothesis significance testing with a Bayes factor from null-hypothesis Bayesian testing. Nurse Researcher. 2020 Dec 16;28(4).
44. Sribney W. Chi-squared test for models estimated with robust standard errors. StataCorp, August. http://www. stata. com/support/faqs/stat/chi2. html. 1997.
45. Ulimwengu JM, Sanyal P. Joint estimation of farmers' stated willingness to pay for agricultural services.
46. Tolera L. *Determinants of Farmer's Willingness to Participate in Soil Conservation Practices the Case of Sabata Hawas Woreda, Oromia Regional State, Ethiopia* (Doctoral dissertation, St. Mary's University).
47. Siew MK, Yacob MR, Radam A, Adamu A, Alias EF. Estimating willingness to pay for wetland conservation: a contingent valuation study of Paya Indah Wetland, Selangor Malaysia. Procedia Environmental Sciences. 2015 Jan 1; 30:268-72.
48. Ansong M, Røskaft E. Local communities' willingness to pay for sustainable forest management in Ghana. Journal of Energy and Natural Resource Management. 2014 Aug 31;1(2).
49. Abdullah S, Mariel P. Choice experiment study on the willingness to pay to improve electricity services. Energy policy. 2010 Aug 1;38(8):4570-81.
50. Abdullah S, Jeanty PW. Willingness to pay for renewable energy: Evidence from a contingent valuation survey in Kenya. Renewable and sustainable energy reviews. 2011 Aug 1;15(6):2974-83.

51. Edkins M. The South African Case for Solar Thermal Power: Assessing the Contribution of CER and TREC Financing. Unpublished MSc, Environmental Change Institute, Oxford University. 2008.
52. Nwofoke C, Onyenekwe SC, Agbo FU. Willingness to pay (WTP) for an improved environmental quality in Ebonyi State, Nigeria. Journal of Environmental Protection. 2017;8(02):131.
53. Nazu SB, Saha SM, Hossain ME, Haque S, Khan MA. Willingness to pay for adopting conservation tillage technologies in wheat cultivation: Policy options for small-scale farmers. Environmental Science and Pollution Research. 2022 Sep;29(42):63458-71.
54. Olum S, Gellynck X, Juvinal J, Ongeng D, De Steur H. Farmers' adoption of agricultural innovations: A systematic review on willingness to pay studies. Outlook on Agriculture. 2020 Sep;49(3):187-203.
55. Gunatilake H, Maddipati N, Patail S. Willingness to pay for good quality, uninterrupted power supply in Madhya Pradesh, India. Asian Development Bank Working Paper Series, No 13. Manila: Asian Development Bank; 2012.
56. Bigerna S, Polinori P. Italian consumers' willingness to pay for renewable energy sources. Renewable and Sustainable Energy Reviews. 2014 Jun 12; 34:110-21.
57. Akcura E. Information effects in valuation of electricity and water service attributes using contingent valuation.
58. Streeten P, Burki SJ. Basic needs: some issues. World Development. 1978 Mar 1;6(3):411-21.
59. Stewart F. Basic needs strategies, human rights, and the right to development. Human Rights Quarterly. 1989 Aug 1;11(3):347-74.
60. Maslow AH. A theory of human motivation. Psychological review. 1943 Jul;50(4):370.

61. Hadker N, Sharma S, David A, Muraleedharan TR. Willingness-to-pay for Borivli National Park: evidence from a contingent valuation. Ecological economics. 1997 May 1;21(2):105-22.
62. Amirnejad H, Khalilian S, Assareh MH, Ahmadian M. Estimating the existence value of north forests of Iran by using a contingent valuation method. Ecological Economics. 2006 Jul 1;58(4):665-75.
