# Supplementary material for: Gendered dimensions of sustainable land management: Evidences from farm size and training effects on willingness to pay in Ghana’s Volta Region
Source: PLoS One. 2026 Jun 11;21(6):e0351424. doi: 10.1371/journal.pone.0351424 (PMC13257986; doi:10.1371/journal.pone.0351424)
Supplement: Attachment — Submitted filename: PONE-D-25-40765_R2 Gender dimention with comments.pdf [file pone.0351424.s013.pdf]

PLOS One

Gendered dimensions of sustainable land management: evidence of farm size and training effects on willingness to pay in Ghana’s Volta Region

--Manuscript Draft--

Manuscript Number:	PONE-D-25-40765R2
Article Type:	Research Article
Full Title:	Gendered dimensions of sustainable land management: evidence of farm size and training effects on willingness to pay in Ghana’s Volta Region
Short Title:	Gendered dimensions of sustainable land management and willingness to pay in Ghana’s Volta Region
Corresponding Author:	James Dickson Fiagborlo<br>Ho Technical University<br>Ho, GHANA
Keywords:	Sustainable land management. Willingness to pay. Gender. Farm size. Training. Ghana.
Abstract:	Land degradation continues to pose a significant threat to agricultural productivity in Ghana, eroding soil fertility and exacerbating rural poverty. Although efforts have been made to promote Sustainable Land Management (SLM), adoption remains limited among smallholder farmers—particularly women—due to persistent disparities in land ownership, training access, and decision-making autonomy. Drawing on survey data from 1,036 farm households across five municipalities in Ghana’s Volta Region, this study investigates the gendered effects of SLM training and farm size on farmers’ willingness to pay (WTP) for SLM practices. A Tobit regression model is employed to estimate both main and interaction effects. The results suggest that both SLM training ($\beta = 0.164$, $p < 0.01$) and farm size ($\beta = 0.058$, $p < 0.01$) significantly increase WTP, highlighting the roles of knowledge acquisition and resource endowment in promoting environmental investment. Significantly, gender moderated these effects: while male farmers initially benefited more from training, female farmers with larger landholdings exhibited higher marginal increases in WTP, suggesting that access to land enhances women’s capacity to invest in SLM. Additionally, WTP is negatively associated with coastal residence, larger household dependency ratios, lower income, and vocational education—factors that reflect broader livelihood and financial constraints. These findings highlight the significance of gender-sensitive SLM interventions that combine equitable access to training with improved access to productive resources. Such targeted strategies aim to increase smallholder participation in sustainable land management and address the socio-economic drivers of land degradation in Ghana.
Order of Authors:	James Dickson Fiagborlo
	Mexoese Nyatuame
	Stella Ahone Ntoko
Response to Reviewers:	In response to reviewer suggestions, the revised manuscript now include DOIs for the majority of the cited papers in the reference list. Where a DOI was not available, full citation details have been provided to maintain traceability.
Additional Information:	
Question	Response
**Financial Disclosure**<br><br>Enter a financial disclosure statement that describes the sources of funding for the work included in this submission. Review the submission guidelines for detailed requirements. View published research articles from *PLOS ONE* for specific	The author(s) received no specific funding for this work.

Powered by Editorial Manager® and ProduXion Manager® from Aries Systems Corporation

examples.<br><br>This statement is required for submission and **will appear in the published article** if the submission is accepted. Please make sure it is accurate.<br><br>**Funded studies**<br>Enter a statement with the following details:<br>• Initials of the authors who received each award<br>• Grant numbers awarded to each author<br>• The full name of each funder<br>• URL of each funder website<br>• Did the sponsors or funders play any role in the study design, data collection and analysis, decision to publish, or preparation of the manuscript?<br><br>Did you receive funding for this work?	
**Competing Interests**<br><br>Use the instructions below to enter a competing interest statement for this submission. On behalf of all authors, disclose any competing interests that could be perceived to bias this work—acknowledging all financial support and any other relevant financial or non-financial competing interests.<br><br>This statement is required for submission and **will appear in the published article** if the submission is accepted. Please make sure it is accurate and that any funding sources listed in your Funding Information later in the submission form are also declared in your Financial Disclosure statement.<br><br>View published research articles from *PLOS ONE* for specific examples.	The authors have declared that no competing interests exist.

**NO authors have competing interests**<br><br>Enter: *The authors have declared that no competing interests exist.*<br><br>**Authors with competing interests**<br><br>Enter competing interest details beginning with this statement:<br><br>*I have read the journal's policy and the authors of this manuscript have the following competing interests: [insert competing interests here]*<br><br>* typeset	
**Ethics Statement**<br><br>Enter an ethics statement for this submission. This statement is required if the study involved:<br><br>• Human participants<br>• Human specimens or tissue<br>• Vertebrate animals or cephalopods<br>• Vertebrate embryos or tissues<br>• Field research<br><br>Write "N/A" if the submission does not require an ethics statement.<br><br>General guidance is provided below. Consult the submission guidelines for detailed instructions. **Make sure that all information entered here is included in the Methods section of the manuscript.**	This research involved structured interviews with adult members of farming households. Prior to participation, all prospective respondents were provided with a detailed explanation of the study's purpose, its voluntary nature, and their right to decline participation or withdraw at any time without any negative consequences. Informed verbal consent was obtained from each participant before the interview commenced.<br>Given the literacy levels in some rural areas and the cultural context of the fieldwork, verbal consent was deemed appropriate and ethically justified. Trained enumerators communicated in either English or Ewe using a standardised script to ensure full understanding. Each instance of verbal consent was recorded in the survey logbook maintained by field team supervisors. Only adult members of the household (aged 18 years and above) were interviewed; minors were excluded from the study. Although formal institutional ethical clearance was not obtained prior to the 2016 fieldwork, all data were fully anonymised, and strict confidentiality procedures were followed. No personally identifying material was collected, and all responses were securely stored and used solely for research purposes. The research procedure adhered to standard ethical practices for social science fieldwork in Ghana at the time of data collection.

Powered by Editorial Manager® and ProduXion Manager® from Aries Systems Corporation

**Format for specific study types**<br><br>**Human Subject Research (involving human participants and/or tissue)**<br>• Give the name of the institutional review board or ethics committee that approved the study<br>• Include the approval number and/or a statement indicating approval of this research<br>• Indicate the form of consent obtained (written/oral) or the reason that consent was not obtained (e.g. the data were analyzed anonymously)<br><br>**Animal Research (involving vertebrate animals, embryos or tissues)**<br>• Provide the name of the Institutional Animal Care and Use Committee (IACUC) or other relevant ethics board that reviewed the study protocol, and indicate whether they approved this research or granted a formal waiver of ethical approval<br>• Include an approval number if one was obtained<br>• If the study involved *non-human primates*, add *additional details* about animal welfare and steps taken to ameliorate suffering<br>• If anesthesia, euthanasia, or any kind of animal sacrifice is part of the study, include briefly which substances and/or methods were applied<br><br>**Field Research**<br><br>Include the following details if this study involves the collection of plant, animal, or other materials from a natural setting:<br>• Field permit number<br>• Name of the institution or relevant body that granted permission	
**Data Availability**<br><br>Authors are required to make all data underlying the findings described fully available, without restriction, and from the time of publication. PLOS allows rare exceptions to address legal and ethical concerns. See the PLOS Data Policy and FAQ for detailed information.	Yes - all data are fully available without restriction

Powered by Editorial Manager® and ProduXion Manager® from Aries Systems Corporation

A Data Availability Statement describing where the data can be found is required at submission. Your answers to this question constitute the Data Availability Statement and **will be published in the article**, if accepted.<br><br>**Important:** Stating 'data available on request from the author' is not sufficient. If your data are only available upon request, select 'No' for the first question and explain your exceptional situation in the text box.<br><br>Do the authors confirm that all data underlying the findings described in their manuscript are fully available without restriction?	
**Describe where the data may be found in full sentences. If you are copying our sample text, replace any instances of XXX with the appropriate details.**<br><br>• If the data are **held or will be held in a public repository**, include URLs, accession numbers or DOIs. If this information will only be available after acceptance, indicate this by ticking the box below. For example: *All XXX files are available from the XXX database (accession number(s) XXX, XXX.)*.<br>• If the data are all contained **within the manuscript and/or Supporting Information files**, enter the following: *All relevant data are within the manuscript and its Supporting Information files.*<br>• If neither of these applies but you are able to provide **details of access elsewhere**, with or without limitations, please do so. For example:<br><br>*Data cannot be shared publicly because of [XXX]. Data are available from the XXX Institutional Data Access / Ethics Committee (contact via XXX) for researchers who meet the criteria for access to confidential data.*<br><br>*The data underlying the results presented in the study are available from (include the name of the third party*	All relevant data are within the manuscript and its Supporting Information files.

and contact information or URL).<br>• This text is appropriate if the data are owned by a third party and authors do not have permission to share the data.<br><br>* typeset	
Additional data availability information:	

Manuscript

Click here to access/download;Manuscript;Manuscript_Clean.docx

Gendered dimensions of sustainable land management: evidence of farm size and training effects on willingness to pay in Ghana's Volta Region

Authors List

James Dickson Fiagborlo[1*], Mexoese Nyatuame[2] and Stella Ahone Ntoko[3]

[1] Department of Multidisciplinary Studies, Faculty of Applied Social Sciences, Ho Technical University, Ho, Ghana (jfiagborlo@htu.edu.gh (J.D. Fiagborlo)); ORCID: 0000-0002-3915-2939

[2] Department of Agric Engineering, Faculty of Engineering, Ho Technical University, Ho, Ghana (mnyatuame@htu.edu.gh (M. Nyatuame)); ORCID: 0000-0001-7652-3308

[3] Centre for Research in Efficient Agricultural Technologies (CREAT), Ho Technical University, Ho, Ghana (nstella15@gmail.com (S.A. Ntoko))

[*] Corresponding Author: jfiagborlo@htu.edu.gh (JDF)

Author Contribution

All authors contributed to the conception and design of the study. Material preparation, data collection, and analysis were conducted by **James Dickson Fiagborlo**, **Mexoese Nyatuame**, and **Stella Ahone Ntoko**. The initial draft of the manuscript was prepared by **James Dickson Fiagborlo**, and all authors read and approved the first and final versions of the manuscript.

Abstract

Land degradation continues to pose a significant threat to agricultural productivity in Ghana, eroding soil fertility and exacerbating rural poverty. Although efforts have been made to promote Sustainable Land Management (SLM), adoption remains limited among smallholder farmers—particularly women—due to persistent disparities in land ownership, training access, and decision-making autonomy. Drawing on survey data from 1,036 farm households across five municipalities in Ghana's Volta Region, this study investigates the gendered effects of SLM training and farm size on farmers' willingness to pay (WTP) for SLM practices. A Tobit regression model is employed to estimate both main and interaction effects. The results suggest that both SLM training ($\beta = 0.164$, $p < 0.01$) and farm size ($\beta = 0.058$, $p < 0.01$) significantly increase WTP, highlighting the roles of knowledge acquisition and resource endowment in promoting environmental investment. Significantly, gender moderated these effects: while male farmers initially benefited more from training, female farmers with larger landholdings exhibited higher marginal increases in WTP, suggesting that access to land enhances women's capacity to invest in SLM. Additionally, WTP is negatively associated with coastal residence, larger household dependency ratios, lower income, and vocational education—factors that reflect broader livelihood and financial constraints. These findings highlight the significance of gender-sensitive SLM interventions that combine equitable access to training with improved access to productive resources. Such targeted strategies aim to increase smallholder participation in sustainable land management and address the socio-economic drivers of land degradation in Ghana.

Keywords: Sustainable land management. Willingness to pay. Gender. Farm size. Training. Ghana.

Introduction

The agricultural landscape in Ghana is facing severe threats from deforestation, overgrazing, and uncontrolled burning, resulting in large-scale land degradation and loss of productivity. An estimated 69% of the agricultural land in Ghana is already eroded, costing the economy an estimated US$4.2 billion annually—equivalent to 5% of agricultural GDP [1, 2]. Land degradation is deepening poverty by reducing soil fertility, lowering yields, and limiting the use of basic inputs such as fertiliser, irrigation, and mechanised tools by farmers [3]. In response to these challenges, there has been a renewed emphasis on Sustainable Land Management (SLM) and Agricultural Sustainable Land Management (ASLM) as key vehicles for restoring soil health and enhancing agricultural resilience [4]. According to TerrAfrica, SLM is "adopting land-use systems that enhance ecological support functions through appropriate management techniques." [5] broaden this definition to cover the role of ASLM in improving the yields of crops, livestock, watersheds, and forests. In this study, SLM and ASLM are used interchangeably.

Notwithstanding policy interest, the adoption of Sustainable Land Management (SLM) practices remains limited in Ghana. Poor financial conditions, inadequate extension support, and erratic access to the requisite technologies have impeded uptake, particularly among smallholder farmers in the Volta Region [6, 7]. Beyond these fundamental barriers, social and behavioural dimensions—especially gender differences in access to training, land, and decision-making power—play a crucial role in shaping adoption behaviour. For instance, studies [8–10] have shown that farmers' willingness to pay (WTP) for environmental and sustainable practices is influenced by socioeconomic factors such as gender, age, education, income, and farm size. While considerable evidence from other developing regions shows that women face disproportionate barriers to land ownership, credit, and training [11, 12], the gendered subtleties of SLM adoption and WTP remain understudied in the context of Ghana.

In theoretical terms, the differences between men and women in land and resource management can be understood through Human Capital Theory [13] and the Gender and Agricultural Decision-Making Framework [11]. These frameworks demonstrate that unequal access to knowledge, skills, and productive assets leads to unequal economic returns. This is indeed the case: land tenure for women is limited, and their access to training and inputs is restricted, ultimately constraining their adoption of, or investment in, Sustainable Land Management (SLM) [14, 15]. This suggests that women's willingness to pay (WTP) is likely to be less influenced by resource endowment and more by social norms, perceived risk, and intra-household decision-making power. Evidence from behavioural studies supports this view and suggests that agricultural decisions are shaped not only by resource availability but also by perceived efficacy and social learning [16, 17].

Empirical studies from Africa [8, 10] and Asia [12, 15] further confirm the importance of gender in sustainable agricultural practices. In Ethiopia, [8] reported that male-headed households were more likely to invest in Sustainable Land Management (SLM), due to greater land ownership and better access to extension services. In Tanzania, for example, [10] shows that women's willingness to pay for watershed services was significantly lower, reflecting gender-based financial barriers. By contrast, [15] reported that female farmers' willingness to participate in environmental conservation programmes increased when training was explicitly designed to empower female participants. Similarly, [12] states that gender-sensitive land governance frameworks in South Korea have empowered women to gain tenure rights and participate fully in land-use decisions. Such findings collectively point to the need for a study into how training, access to land, and gender roles interact to influence SLM-related decisions.

The interaction of Sustainable Land Management (SLM) training with farm size and gender is poorly understood in Ghana. While the aim of such training is to enhance farmers' technical and management knowledge [18], its impacts vary by gender due to fundamental

inequalities in land access and social capital. For example, male farmers may leverage larger farm sizes and greater financial independence to apply knowledge gained through training, whereas females may be constrained, even when exposed to similar training opportunities. Understanding the gendered effects of training and farm size on willingness to pay (WTP) for SLM practices is therefore key to designing equitable agricultural interventions.

The study addresses gaps in the existing literature by examining the gendered effects of Sustainable Land Management (SLM) training and farm size on farmers' willingness to pay (WTP) for SLM practices in Ghana's Volta Region. Specifically, it aims to: (1) identify the effect of SLM training on WTP for SLM practices among male and female farmers; (2) explore the impact of farm size on WTP for SLM practices across genders; and (3) assess the gendered dimensions of overall WTP for SLM practices.

This study contributes to the growing literature on gender and sustainable agriculture in three key ways. First, it provides empirical evidence on how training and resource endowments (farm size) interact with gender to shape sustainability-oriented behaviour. Second, through the application of a gender-sensitive analytical framework, it enriches the literature on environmental economics in the context of valuing Sustainable Land Management (SLM) practices. Third, it generates policy-relevant insights to support gender-equitable land management and climate adaptation strategies.

The remainder of the paper is structured as follows: Section 2 presents a review of the literature on Sustainable Land Management and land policy in Ghana; Section 3 outlines the methodological framework; Section 4 presents and discusses the empirical results; and Section 5 concludes with policy implications, limitations, and recommendations for future research.

Literature review

This section reviews land management policies in Ghana and synthesises empirical evidence on Sustainable Land Management practices, training, farm size, gender, and farmers' willingness to pay. It draws on both African and global contexts to provide a comprehensive foundation for the development of the hypotheses of this study.

Land management techniques and policies in Ghana

Ghana's National Land Policy, enacted in 1999, seeks to establish an efficient legal and administrative framework for the management of land rights, to recognise traditional authorities, and to ensure the sustainable use of land in the context of a growing population and increasing land-use pressures [19, 20]. The policy addresses challenges such as unregulated land markets, multiple sales, encroachment, and unplanned development. Its major goals are to ensure tenure security, enhance productivity, and promote sustainable land use [1, 19].

Implementation strategies include the preparation of national-and district-level land-use plans, the formation of inter-ministerial technical groups for dispute resolution, and, importantly, inclusive decision-making involving all relevant stakeholders [21]. The policy also prohibits forest clearing for mining purposes, based on the premise that sound land-use planning can reduce conflicts and improve ecological outcomes.

Other complementary frameworks include the Forest and Wildlife Policy, Fire Policy, and the Sustainable Land Management Policy. These were also introduced with a view to enhancing integration of natural resource management in agriculture [5]. However, their implementation faces challenges characterised by weak institutional capacity, limited enforcement, and poor coordination among stakeholders [22]. Farmers continue to engage in

unsustainable practices such as slash-and-burn cultivation, driven by economic necessity and/or a lack of awareness about the long-term effects of land degradation [23].

The implementation of the Food and Agriculture Sector Development Policy (FASDEP), the Northern Rural Growth Programme, and the Ghana Strategic Investment Fund (GSIF) has advanced Sustainable Land Management initiatives. However, inadequate community participation and gender-insensitive approaches have generally constrained the impact achieved thus far by these programmes [24]. A growing body of research calls for multi-stakeholder, gender-inclusive land governance models that integrate the voices of farmers, extension officers, and local institutions in ways that promote long-term sustainability [6].

Empirical literature on Sustainable Land Management and gender

Empirical research across the African continent confirms that Sustainable Land Management practices, including conservation agriculture, agroforestry, and integrated soil fertility management, improve yields, enhance soil health, and strengthen climate resilience [4, 25]. However, adoption rates remain low, particularly among smallholder farmers, due to limited access to finance, inadequate extension services, and weak policy support [7]. Economies of scale, access to credit, and lower transaction costs typically lead larger farms to exhibit higher adoption rates [8]. Nevertheless, smallholder farmers who receive training also demonstrate greater uptake of conservation practices [25].

Training is generally regarded as a key determinant of Sustainable Land Management (SLM) adoption and willingness to pay (WTP), as it improves farmers' understanding of sustainable practices and enhances their perception of long-term benefits associated with these interventions [10, 26]. However, training opportunities often remain gendered. In many rural African contexts, men are more likely to attend agricultural training sessions or receive extension services due to social norms, greater time flexibility, and fewer restrictions on

mobility [11]. For example, studies from Ethiopia [8] and Tanzania [10] report that female farmers exhibit lower WTP for SLM-related services, not due to disinterest, but rather as a result of structural inequalities in access to resources.

Recent studies further underscore the intersectional relationship between gender, access to resources, and environmental decision-making across Sub-Saharan Africa and Asia. For instance, [15] observed that in Nepal, women's willingness to participate in conservation agriculture significantly increased when training was tailored to their empowerment. Similarly, [12] noted that a gender-sensitive land governance framework in South Korea improved women's tenure security and participation in sustainable land decisions. However, evidence from Ethiopia and Kenya indicates that male-headed households remain more likely to adopt capital-intensive Sustainable Land Management practices, largely due to their greater access to credit and mechanisation facilities [14, 25].

Gender differences in willingness to pay and adoption behaviour can also be analysed through the lens of behavioural science. According to [16] and [17], risk perceptions, trust, and social learning are major drivers of farmers' environmental decisions. In particular, women's incentives for participating in SLM initiatives are often shaped by community networks and collective learning prospects, rather than by individual resource endowments. This highlights the need for both material and social responses to foster truly gender-equitable participation—addressing disparities in access to resources as well as differences in behavioural motivations.

Farm size, SLM training, gender, and willingness to pay

Farm size remains a critical factor affecting willingness to pay (WTP). Larger farmers can spread the fixed costs of adoption over greater acreage, thereby realising scale efficiencies [25]. Empirical evidence from Nigeria [27], Egypt [28], and China [29] indicates that farm size and income are significant predictors of higher WTP for irrigation and soil management practices.

However, studies also reveal that beyond a certain threshold, larger farms may experience diminishing marginal returns to investment in Sustainable Land Management practices [8].

Training interacts with farm size in shaping adoption outcomes, as larger farmers are typically better positioned to apply knowledge gained from Sustainable Land Management training, while smaller farmers may struggle to translate knowledge into practice due to financial constraints [25]. Gender further moderates this interaction; female farmers, even when trained, may not fully benefit from training if they do not own land or lack access to complementary inputs such as credit or tools [11, 15].

Summary of literature review and hypotheses development

Although previous studies provide valuable insights into Sustainable Land Management (SLM) adoption and willingness to pay (WTP), several critical gaps persist. First, there is a limited understanding of how SLM training, farm size, and gender interact to shape farmers' WTP for SLM practices. Second, the gendered dimensions of WTP—including differences in decision-making power, access to credit, and exposure to training —remain underexplored in Ghana. Third, literature rarely integrates behavioural perspectives that explain how risk perception and social norms influence WTP differently for male and female farmers. To address these gaps, this study investigates how gender moderates the effects of SLM training and farm size on farmers' WTP for SLM practices in Ghana's Volta Region. Guided by the theoretical and empirical literature, the following hypotheses are proposed:

H1: Farmers who received SML training will demonstrate higher WTP for SLM practices than those who have not.

H2: Larger farm sizes are associated with higher WTP for SLM practices.

H3: Female farmers with equitable access to training and resources will exhibit higher WTP for SLM practices than male farmers.

This gender-focused framework recognises that SLM decisions are shaped not only by economic capacity but also by institutional, behavioural, and social dynamics.

Materials and methods

Study design and sampling procedure

This study investigates gendered dimensions of Sustainable Land Management (SLM) training, farm size, and willingness to pay (WTP) for SLM practices among farming households in Ghana's Volta Region. A multistage sampling technique was employed to ensure balanced representation across ecological and administrative clusters. In the first stage, five municipalities were purposively selected to capture the region's major ecological zones. Within each municipality, five communities were randomly selected, yielding a total of 25 study sites. In the second stage, 50 farm households were randomly selected from each community using a simple random sampling technique, resulting in a total sample of 1,250 households. This stratified design accounts for potential heterogeneity across localities—such as variation in rainfall, soil fertility, and access to agricultural extension services—thereby enhancing representativeness and guaranteeing statistical reliability.

Due to missing information on some variables, the final sample was reduced to 1036 households. Following [30], this sample size was deemed adequate to achieve the study's objectives. Moreover, it exceeds the minimum thresholds recommended by [31] and [32] for a 95% confidence level, thereby ensuring statistical adequacy and analytical robustness. This research involved structured interviews with adult members of farming households. Prior to participation, all prospective respondents were provided with a detailed explanation of the study's purpose, its voluntary nature, and their right to decline participation or withdraw at any

time without any negative consequences. Informed verbal consent was obtained from each participant before the interview commenced.

Given the literacy levels in some rural areas and the cultural context of the fieldwork, verbal consent was deemed appropriate and ethically justified. Trained enumerators communicated in either English or Ewe using a standardised script to ensure full understanding. Each instance of verbal consent was recorded in the survey logbook maintained by field team supervisors. Only adult members of the household (aged 18 years and above) were interviewed; minors were excluded from the study. Although formal institutional ethical clearance was not obtained prior to the 2016 fieldwork, all data were fully anonymised, and strict confidentiality procedures were followed. No personally identifying material was collected, and all responses were securely stored and used solely for research purposes. The research procedure adhered to standard ethical practices for social science fieldwork in Ghana at the time of data collection.

Data, description, and measurement of variables

The questionnaire collected detailed socio-demographic and farm-level information necessary to analyse the factors impacting farmers' willingness to pay for Sustainable Land Management (SLM) practices. Respondents provided data on age, gender, education, income, farming experience, number of household dependants, farm size, and participation in SLM training programmes. A community location variable (coastal vs. inland) was also included to control for geographic and environmental heterogeneity.

The dependent variable, willingness to pay (WTP), was elicited through a contingent valuation (CV) question, framed as follows: *"SLM practices serve multiple functions: they prevent soil erosion, enhance water retention, support groundwater recharge, and mitigate flooding. In view of these benefits, what is the maximum amount in Ghanaian Cedis (GHS) you are willing to pay to sustain the environmental benefits provided by SLM practices?"*

Respondents who reported zero WTP were classified as non-payers, while positive responses were recorded as the maximum amount they were willing to pay.

Table 1 summarises the measurement of all variables used in the analysis. The key explanatory variables include Sustainable Land Management (SLM) training, a binary variable indicating whether the respondent has received any form of SLM-related training, and farm size, measured as the total land area (in acres) cultivated by the farmer. Gender is coded as a binary variable, where 1 represents male respondents and 0 represents female respondents. To isolate the impacts of socio-economic characteristics on willingness to pay (WTP), several control variables are included: household income, years of farming experience, number of household dependants, and education. Household income is measured as a continuous variable

Table 1. Measurement of variables

Variable	Measurement	Min	Max
**WTP**	Continuous: non-negative censored variable (GHS)	0	500
**SLM training**	Binary: 1 = farmer received SLM training; 0 = otherwise	0	1
**Farm size**	Continuous: farm size in acres	1	45
**Male**	Binary: 1= male farmer; 0 = otherwise	0	1
**Coastal**	Binary: 1 = coastal resident; 0 = otherwise	0	1
**Income**	Continuous: monthly income (GHS)	30	1000
**Household dependant**	Continuous: number of household dependants	1	18
**Farmer experience**	Continuous: years of farming experience	1	60
**No education**	Binary: 1 = no formal education; 0 = otherwise	0	1
**Primary education**	Binary: 1 = primary education; 0 = otherwise	0	1
**Middle sch. education**	Binary: 1 = middle sch. education; 0 = otherwise	0	1
**Secondary education**	Binary: 1 = secondary education; 0 = otherwise	0	1
**Vocational education**	Binary: 1 = vocational training; 0 = otherwise	0	1
**Tertiary education**	Binary: 1 = tertiary education; 0 = otherwise	0	1

NB: Max= Maximum; Min= Minimum

capturing monthly earnings. Farming experience, measured in years, reflects accumulated agricultural knowledge, which may influence risk perception and investment behaviour.

Education is represented by categorical (dummy) variables indicating different levels of attainment, ranging from no formal education to tertiary-level qualifications. The minimum and maximum values presented in Table 1 provide a comprehensive overview of variation within the sample and support the replicability of the empirical analysis.

Econometric model

Given that willingness to pay (WTP) is a non-negative continuous variable censored at zero, a Tobit regression model estimated via Maximum Likelihood Estimation was employed [33]. This modelling approach is appropriate when the dependent variable is observed only above or at a certain threshold, in this case, zero, and it accommodates both the probability of being above the threshold and the conditional mean of the uncensored observations. Previous studies have employed similar approaches to estimate WTP and other censored outcomes, noting the Tobit model's ability to account for the limited range of values while preserving estimation efficiency [34,35]. The empirical model is specified as follows:

$$WTP*_i = \beta_0 + \beta_1 SLMT_i + \beta_2 FAMS_i + \beta_3 GM_i + \beta_4(\mathrm{SLMT_i \ x\ FAMS_i}) + \beta_5(\mathrm{SLMT_i \ x\ GM_i}) + \beta_6(\mathrm{FAMS_i \ x\ GM_i}) + \beta_7(\mathrm{SLMT_i \ x\ GM_i \ x\ FAMS_i}) + \beta_8 COC_i + \beta_9 INC_i + \beta_{10} HD_i + \beta_{11} FE_i + \beta_{12} ED_i + \varepsilon_i$$

where WTPi∗ represents the latent (unobserved) willingness to pay. The observed WTP is defined as $\mathrm{WTP_i} = \mathrm{WTP*_i}$, if $WTP*_i > 0$ and $WTP_i = 0$, if $WTP*_i \leq 0$. β s are the coefficients associated with the predictors, and ε_i denotes the stochastic term, which is considered to be independent and normally distributed with a zero mean and constant variance. The set of independent variables includes Sustainable Land Management training (SLMT), farm size (FAMS), gender (GM), coastal location (COC), income (INC), number of household dependants (HD), farming experience (FE), and education level (ED). To examine moderating

effects, the model incorporates interaction terms: SLMT x FAMS, SLMT x GM, GM x FAMS, and SLMT x GM x FAMS. These interactions capture the combined influence of training, farm size, and gender on willingness to pay (WTP) for Sustainable Land Management practices.

As part of the analytical strategy, descriptive statistics were computed to summarise household and farm-level characteristics (Table 3). Following [36], average marginal effects (AMEs) were derived from the Tobit model to provide insight into both the direction and magnitude of relationships among variables. All analyses were conducted using Stata version 15, with robust standard errors applied to address potential heteroskedasticity.

Diagnostic tests

Table 2 presents an assessment of multicollinearity among independent variables using Variance Inflation Factors (VIF) and condition number. The highest VIF—3.03 for the middle school education category—falls well below conventional multicollinearity thresholds of

Table 2: Estimated VIF for the variables used in the analysis

Variable	VIF	SqrtVIF	Toleran	R-squared
**SLM training**	1.08	1.04	0.929	0.071
**Farm size**	1.23	1.11	0.815	0.185
**Gender (Male)**	1.14	1.07	0.879	0.121
**Coastal**	1.17	1.08	0.855	0.145
**Income**	1.18	1.09	0.848	0.152
**Farmer experience**	1.08	1.04	0.927	0.073
**Household dependant**	1.06	1.03	0.948	0.052
**Primary education**	2.32	1.52	0.431	0.569
**Middle school education**	3.03	1.74	0.330	0.670
**Secondary education**	1.92	1.39	0.520	0.480
**Vocational education**	1.44	1.20	0.696	0.304
**Tertiary education**	1.45	1.20	0.691	0.309
**Mean VIF**		1.51		
**Breusch-Pagan/Cook-Weisberg test for heteroskedasticity**				
**Chi^2**		28.36		
**Prob> Chi^2**		0.000		

greater than 5 or 10 [35], indicating no serious collinearity issues. Additionally, the condition number (12.5) and the overall mean VIF (1.51) are below the generally accepted threshold of 15, further suggesting that multicollinearity is not a concern [37–40]. The sample size of 1036 exceeds the minimum threshold that [31] recommended, ensuring statistical adequacy and model robustness. To test for heteroskedasticity, the Breusch-Pagan or Cook-Weisberg test was conducted (Table 2). The test results indicated statistically significant heteroskedasticity, suggesting that the variance of the error terms is not constant across observations. Accordingly, robust standard errors were applied in all the regression analyses to correct for this violation and to ensure valid statistical inference. Overall, the suitability of the Tobit model is supported by these diagnostic checks and confirmed by a combination of robustness checks (Table 5), which provide confidence in the model estimates and justify the appropriateness of using the Tobit specification. The next section presents the summary statistics and empirical results.

Results and discussions

Table 3 presents the descriptive statistics of the variables used in the analysis. The average stated willingness to pay (WTP) for Sustainable Land Management (SLM) practices across all respondents is GHS 76.59 (US$19.15)[1]. While this mean value covers all survey participants, it is important to indicate that the median WTP is GHS 0. This implies that more than 50% of the farmers reported no WTP, indicating that a relatively small subgroup of farmers is driving the mean up through high stated willingness to pay values. To estimate aggregate WTP, the mean WTP was multiplied by the estimated number of agricultural households. According to the 2010 Population and Housing Census, 888,271 individuals in the Volta Region were engaged in agriculture. Using the national average household size of 4.4, the number of regional agricultural households was estimated to be about 201,879.77. Nationally, with 10.48 million individuals engaged in agriculture, the number of agrarian households was estimated

Table 3. Summary statistics

Variables	Observation	Mean	SD
**Willingness to pay (WTP)**	1036	76.59	118.24
**SLM training**	1036	0.35	0.48
**Farm size**	1036	4.00	3.57
**male**	1036	0.60	0.49
**coastal**	1036	0.54	0.50
**Income**	1036	346.62	207.62
**Household dependant**	1036	4.96	2.40
**Farmer experience**	1036	11.47	10.05
**No education**	1036	0.10	0.30
**Primary education**	1036	0.19	0.39
**Middle school education**	1036	0.52	0.50
**Secondary education**	1036	0.10	0.30
**Vocational education**	1036	0.05	0.21
**Tertiary education**	1036	0.04	0.19

NB: SD means standard deviation

[1]*Exchange rate is US$1 = GHS4—the average daily exchange at the time of data collection (October 2016 to November 2016). This was retrieved from the Bank of Ghana at https:// www. bog. gov.gh/ treasury- and- the- markets/ historical-interbank- fx- rates/*

to be around 2.4 million. Applying the mean WTP to these populations yields an estimated total WTP of GHS15.46 million (US$3.87 million) for the Volta Region and GHS182.41 million (US$45.60 million) for Ghana. These figures offer an indication of the total economic value that farming households place on the environmental benefits of SLM practices.

The average willingness to pay of GHS 76.59 represents approximately 19% of the average monthly farm income, suggesting that farmers who are willing to pay perceive the benefits of Sustainable Land Management as economically meaningful. Conversely, these estimates should be interpreted with caution, as they echo stated preferences under hypothetical conditions. Actual payments in a realistic context may not be the same due to budget constraints, behavioural influences or contextual difficulties.

Descriptive statistics reveal that 35% of farmers had received Sustainable Land Management training, while the majority (54%) lived in coastal areas. The average monthly income was GHS346.62 (US$86.66). Male farmers constituted 60% of the sample (Table 3). On average, farmers had five dependants and 12 years of farming experience. Educational attainment was quite high; only 10% of the farmers had no formal education, while 90% had received some level of education up to the tertiary level. The average farm size was four acres. Farmers' reluctance to pay for Sustainable Land Management (SLM) practices can be attributed to several factors. Approximately 177 farmers (31%) indicated that they believed funding for SLM initiatives should be the responsibility of the government (Fig 1). Another 89 farmers (16%) reported that high farm-related expenditures were already overwhelming them. Moreover, 84 farmers (15%) stated that they would be willing to pay only if they observed

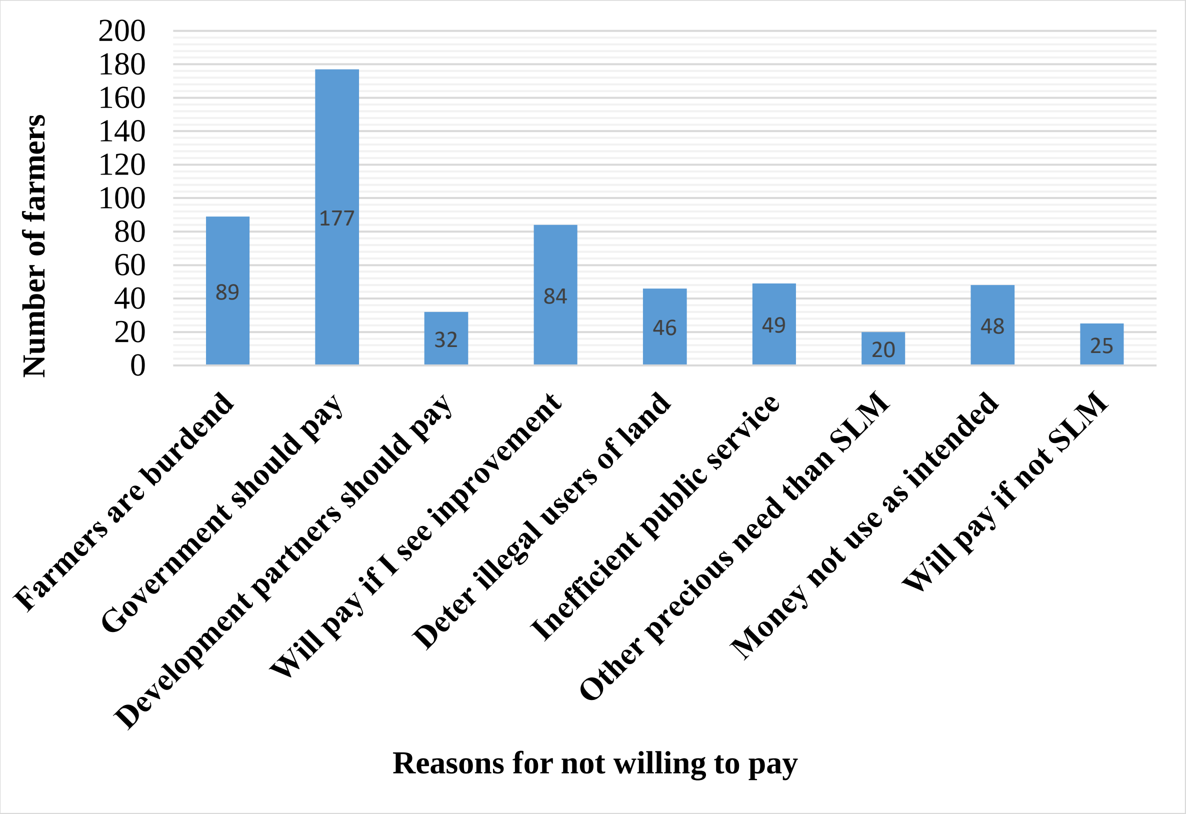

Fig 1: Reasons for not being willing to pay for SLM service

tangible improvements in SLM implementation. A further 49 farmers (9%) conveyed distrust in public institutions, stating that government employees were ineffective and that contributions made by farmers might be pocketed. Additionally, 25 farmers (4%) reported that they would only be willing to pay if SLM activities were not implemented at all, perhaps showing an inherent misunderstanding of SLM goals. These findings suggest the need for policymakers to understand farmers' genuine fears, mainly regarding trust in public officials and the perceived effectiveness of SLM interventions.

Public trust is a critical determinant of willingness to pay, as suggested by empirical data [41]. Over 15% of farmers said that they would consider paying only if SLM services were better. According to the Centre for Environment and Development (CED), unreliable services reduce public confidence and, as a result, willingness to contribute financially. As observed in past studies, confidence in the institutions responsible for managing public funds significantly influences willingness to pay for public goods and services [42]. When individuals believe their donations will be used effectively, resistance to paying declines. On the other hand, unresolved concerns about governance and accountability may suppress WTP and also hinder future efforts to introduce cost-sharing models in environmental management [41].

Empirical results

Table 4 presents the average marginal effects (AMEs) and summary statistics of key predictors on willingness to pay (WTP) for Sustainable Land Management (SLM) practices. A total of eight models were estimated in all. Model 1 reports the baseline Tobit specification. Models 2 and 3 adopt a two-part model technique for robustness: Model 2 represents a logit regression estimating the probability of being a payer (i.e., WTP > 0), while Model 3 presents the ordinary least squares (OLS) regression estimating the level of WTP among those who reported positive values. Models 4 through 7 investigated heterogeneous effects by estimating separate Tobit

models for different sub-samples. In particular, Models 4 and 5 examine gender differences by estimating Tobit regression separately for male and female farmers, while Models 6 and 7 do the same for farmers with low and high levels of farming experience. Additionally, a Heckman selection model was estimated (Appendix Table 6) as an additional robustness check to correct for potential selection bias arising from a non-random decision to state a WTP amount. Robust standard errors are reported in parentheses for all model estimates.

All the models are statistically significant at conventional levels, with the F-statistics ranging from 3.81 to 8.52 and associated p-values of 0.000 [43, 44]. This provides strong evidence against the joint null hypothesis that all coefficients are equal to zero, confirming the overall statistical significance and model stability within the context of the data. The pseudo-R^2 values, which range between 0.05 and 0.10, indicate that the models explain approximately 5% to 10% of the variations in the dependent variable. While these values appear low, they are not uncommon in models based on limited dependent variables, such as Tobit or logit models, where pseudo-R^2 does not carry the same interpretation as R^2 in linear regression. As noted in the literature, pseudo-R^2 values can be sensitive to model specification and may fall outside the 0–1 range in certain likelihood-based models [45]. Nevertheless, they provide a useful, albeit approximate, measure of model fit.

Table 4: Average marginal effects of predictor variables on WTP for SLM practices

Variables	Model 1	Model 2	Model 3	Model 4	Model 5	Model 6	Model 7
**Training (SLMT)**	0.164***	0.116***	0.537***	0.107***	0.282***	0.145***	0.170***
	(0.029)	(0.033)	(0.118)	(0.038)	(0.044)	(0.042)	(0.041)
**Log of farm size(lfsize)**	0.058***	0.074***	-0.011	0.054**	0.067**	0.111***	0.021
	(0.020)	(0.022)	(0.089)	(0.024)	(0.033)	(0.027)	(0.027)
**Gender (Male)**	0.030	0.007	0.220*	-	-	0.015	0.036
	(0.027)	(0.032)	(0.114)	-	-	(0.037)	(0.040)
**SLMT x lfsize**	0.414**	0.548***	2.010***	0.310	0.685***	0.378**	0. 468**
	(0.142)	(0.029)	(0.112)	(0.207)	(0.176)	(0.197)	(0.203)

**SLMT x Male**	0.315*	0.512***	2.062***	-	-	0.300	0.291
	(0.174)	(0.035)	(0.133)	-	-	(0.280)	(0.220)
**lfsize x Male**	–0.167	0.459***	1.749***	-	-	–0.117	–0.222
	(0.110)	(0.021)	(0.089)	-	-	(0.156)	(0.156)
**SLMT x Male x lfsize**	0.375**	0.522***	2.089***	-	-	0.290	0. 438*
	(0.196)	(0.038)	(0.157)	-	-	(0.299)	(0.258)
**Coastal**	–0.169***	–0.214***	–0.038	–0.222***	–0.070	–0.208***	–0.123***
	(0.028)	(0.029)	(0.128)	(0.035)	(0.044)	(0.038)	(0.040)
**Log of income**	0.046**	0.039*	0.121	0.065**	0.004	0.035	0.065**
	(0.019)	(0.023)	(0.077)	(0.026)	(0.028)	(0.025)	(0.030)
**Log of experience**	–0.001	–0.009	0.036	0.007	–0.016	-	-
	(0.016)	(0.018)	(0.067)	(0.020)	(0.025)	-	-
**Household dependant**	–0.066**	–0.080***	0.000	–0.042	–0.085**	–0.082**	–0.033
	(0.026)	(0.028)	(0.115)	(0.034)	(0.038)	(0.036)	(0.036)
**Primary education**	0.043	0.000	0.377*	0.086	0.016	–0.005	0.068
	(0.049)	(0.056)	(0.229)	(0.076)	(0.065)	(0.071)	(0.065)
**Middle sch. education**	0.049	0.034	0.234	0.107	0.009	–0.017	0.090
	(0.043)	(0.049)	(0.201)	(0.067)	(0.059)	(0.063)	(0.055)
**Secondary education**	–0.008	–0.108	0.765***	0.129	–0.354***	–0.113	0.078
	(0.064)	(0.068)	(0.292)	(0.084)	(0.105)	(0.089)	(0.085)
**Vocational education**	–0.193**	–0.244***	0.098	–0.220*	–0.103	–0.143	–0.188*
	(0.083)	(0.090)	(0.322)	(0.127)	(0.113)	(0.123)	(0.107)
**Tertiary education**	–0.050	–0.061	0.060	0.020	–0.172	–0.122	–0.013
	(0.076)	(0.094)	(0.315)	(0.092)	(0.189)	(0.105)	(0.100)
**Observations**	1,036	1,036	472	622	414	504	532
**Log/pseudolikelihood**	–1337.92	–649.052	-	–815.154	–507.002	–651.142	–677.88
**LR /Wald chi^2**	-	114.22	-	-	-	-	-
**F(x, n)**	8.51		3.81	8.59	4.86	5.62	4.60
**Pseudo R^2/R^2**	0.050	0.091	0.10	0.06	0.06	0.06	0.05
**Prob> F**	0.000	0.000	0.000	0.000	0.000	0.000	0.000

Robust standard errors in parentheses * *p < 0.01*, ** *p < 0.05*, * *p < 0.1***

NB: Model 1=baseline model (Tobit); Models 2&3= Two-part model (Logit &OLS) for robustness check; Models 4 &5=gendered subsample model (Tobit); Models 6&7=Farmer experience subsample model (Tobit)

Robustness, heterogeneity, and endogeneity considerations

To strengthen confidence in the baseline results, several robustness and validity checks were conducted. As shown in Table 4, the direction and statistical significance of key predictor variables—particularly SLM training and farm size—remain consistent across alternative model specifications, confirming the robustness of the baseline Tobit estimates. Second, the sub-sample analyses (Models 4-7) reveal heterogeneity in effects across gender and farming experience. While the effects of SLM training and farm size remain positive and statistically significant in all groups, the magnitude of these effects differs. In effect, SLM training has a stronger marginal effect among female and less-experienced farmers, stressing the importance of targeted capacity-building interventions for these groups [16, 25]. Third, the statistically significant Inverse Mills Ratio (IMR = –1.411, $p < 0.05$) in the Heckman model confirms the presence of selection bias in the Tobit estimates, thereby justifying the use of the Heckman method. Significantly, however, the sign and magnitude of the key variables remain stable across models, reinforcing the robustness and internal consistency of the baseline results. The next section presents the results of the baseline Tobit model (Model 1) in greater detail.

Main effects

This section examines the main effects of SLM training, farm size, and gender on farmers' willingness to pay (WTP) for Sustainable Land Management (SLM) practices. In model 1, SLM training emerges as a positive and statistically significant predictor of WTP ($\beta = 0.164$, $p < 0.01$), thereby confirming Hypothesis 1 (H1). This finding suggests that, holding farm size constant (i.e., lfsize = 0) and assuming female gender (i.e., male = 0), those who receive SLM training demonstrate a higher WTP for sustainable practices. This result is consistent with previous studies that highlight the role of training in empowering farmers and enhancing the adoption of SLM interventions [8, 15, 25].

Similarly, farm size is a positive and statistically significant predictor of WTP (β = 0.058, $p < 0.01$), confirming Hypothesis 2 (H2). In the absence of SLM training (SLMT=0) and among female farmers (Male=0), those with larger landholdings demonstrate a greater willingness to pay for SLM practices. This result supports the economies of scale argument [27, 28], whereby larger farms reduce per-unit investment costs, thus making adoption more feasible and attractive. It also aligns with previous studies that identity access to productive resources as a key enabler of sustainable practice adoption [11, 14].

Conversely, gender is a positive but statistically insignificant predictor of WTP for SLM practice (0.030, $p > 0.1$). This suggests that, in the absence of both SLM training (SMLT = 0) and land resources (lfsize = 0), there is no statistically significant difference in WTP between male and female farmers. However, gender may play a more substantial role when interacting with other variables such as training and land access, as explored in the next section.

Interaction effects

This section examines how combinations of SLM training, gender, and farm size interact to influence farmer ‘willingness to pay (WTP) for SLM practices. The inclusion of interaction terms helps identify conditional effects that are not captured by the main effects alone. The interaction between SLM training and gender *(SLMT x Male)* is positive and marginally significant (β = 0.0.315, $p < 0.1$), indicating that male farmers benefit more from SLM training than their female counterparts. This may reflect underlying gender disparities in access to productive assets, information, and decision-making authority [11, 12].

The interaction between SLM training and farm size *(SLMT x lfsize)* is positive and statistically significant (β = 0.414, $p < 0.05$), suggesting that the effect of training on willingness to pay is more pronounced among farmers with larger landholdings. This finding reinforces Hypothesis 2 (H2) and supports the economies of scale argument [27, 28], where larger farms

reduce per-unit investment costs. It also aligns with previous studies that identify access to resources and technical knowledge are key drivers of sustainable land management adoption [11, 14]. In contrast, the interaction between farm size and gender *(lfsize x male)* is negative and statistically insignificant ($\beta = –0.167$, $p < 0.1$), implying that gender does not significantly moderate the effect of farm size on WTP.

Notably, the three-way interaction between SLM training, gender, and farm size (SLM training × gender × farm size) is positive and statistically significant ($\beta = 0.375$, $p < 0.05$), confirming Hypothesis 3 (H3). This indicates that the effect of SLM training on willingness to pay (WTP) is jointly moderated by both gender and farm size. Specifically, the highest WTP is observed among male farmers with larger farms who have received SLM training. This finding underscores the relevance of adopting an intersectional approach when analysing agricultural decision-making and sustainability behaviours, as the interplay of socio-economic factors shapes farmers' adoption capacity and preferences.

Total marginal effects

This section presents the total marginal effects of SLM training, gender and farm size on willingness to pay (WTP) for Sustainable Land Management (SLM) practices, derived by combining both main effects and interaction effects. This comprehensive approach provides a clearer understanding of how these variables jointly influence WTP in real-world farming contexts. Table 5 summarises the total marginal effects across different combinations of gender, farm size, and training status. The results indicate that SLM training significantly increases WTP for all farmer groups; however, its impact is notably greater among farmers with larger landholdings, particularly male farmers. Among female farmers, the marginal effect of training increases from 0.164 to 0.578 as farm size increases. For male farmers, the effect

increases more sharply–from 0.479 to 1.268–suggesting that SLM training is most effective when combined with greater access to land and likely enhanced decision-making power [15].

Table 5: Total marginal effects of key predictors on WTP for SLM practices

A: Total Marginal effects of SLM training on WTP for SLM practices by farm size and gender

Farmer Type	B1	B4	B5	B7	**Total marginal effects**
**Male=0 (lfsize=1)**	0.164	0.414	0	0	0.578
**Male=1 (lfsize=1)**	0.164	0.414	0.315	0.375	1.268
**Male=0 (lfsize=0)**	0.164	0	0	0	0.164
**Male=1 (lfsize=0)**	0.164	0	0.315	0	0.479

B: Total Marginal effects of farm size on WTP for SLM practices by SLM training and gender

Farmer Type	**B2**	**B4**	**B6**	**B7**	**Total marginal effects**
**Male=0 (SLMT=1)**	0.058	0.414	0	0	0.472
**Male=1 (SLMT=1)**	0.058	0.414	–0.167	0.375	0.680
**Male=0 (SLMT=0)**	0.058	0	0	0	0.058
**Male=1 (SLMT=0)**	0.058	0	–0.167	0	–0.109

B: Total Marginal effects of gender on WTP for SLM practices by SLM training and farm size

Farmer Type	**B3**	**B5**	**B6**	**B7**	**Total marginal effects**
**SLMT=0 (lfsize=1)**	0.03	0	–0.167	0	–0.137
**SLMT=1 (lfsize=1)**	0.03	0.315	–0.167	0.375	0.553
**SLMT=0 (lfsize=0)**	0.03	0	0	0	0.03
**SLMT=1 (lfsize=0)**	0.03	0.315	0	0	0.345

NB: B1=main effect of SLM training; B2=main effect of farm size; B3=main effect of gender; B4= SLMT x lfsize; B5= SLMT x Male; 56= lfsize x Male; B7= SLMT x Male x lfsize

Farm size demonstrates a more substantial effect on willingness to pay (WTP) when combined with SLM training. Among farmers who received SLM training, the marginal effect of farm size on WTP is 0.680 for men and 0.472 for women, indicating that training enhances the value of landholding in motivating sustainable investment. However, among untrained male farmers, the total marginal effect of farm size is negative (-0.109), suggesting that landholding alone is insufficient to drive willingness to investment in sustainable practices without complementary training or behavioural incentives [9].

Although gender alone is not statistically significant, its conditional effect becomes evident when combined with SLM training and farm size. The total effect of being male rises from 0.030 (in the absence of both SLM training and farm size) to 0.553 (when both are present), confirming that gendered patterns of investment are highly context-dependent. These results support existing evidence that gender gaps in agricultural adoption are mediated by differential access to resources and training opportunities [11, 12].

To visually support these estimates, Figure 2 presents the predictive margins, illustrating how willingness to pay (WTP) for SLM practices varies significantly by both gender and farm size. Among female farmers who received SLM training, WTP increases consistently with farm size, indicating that the benefits of training are amplified by greater access to land resources. Conversely, trained male farmers exhibit a declining WTP trend as farm size increases, indicating diminishing marginal returns to training among those who

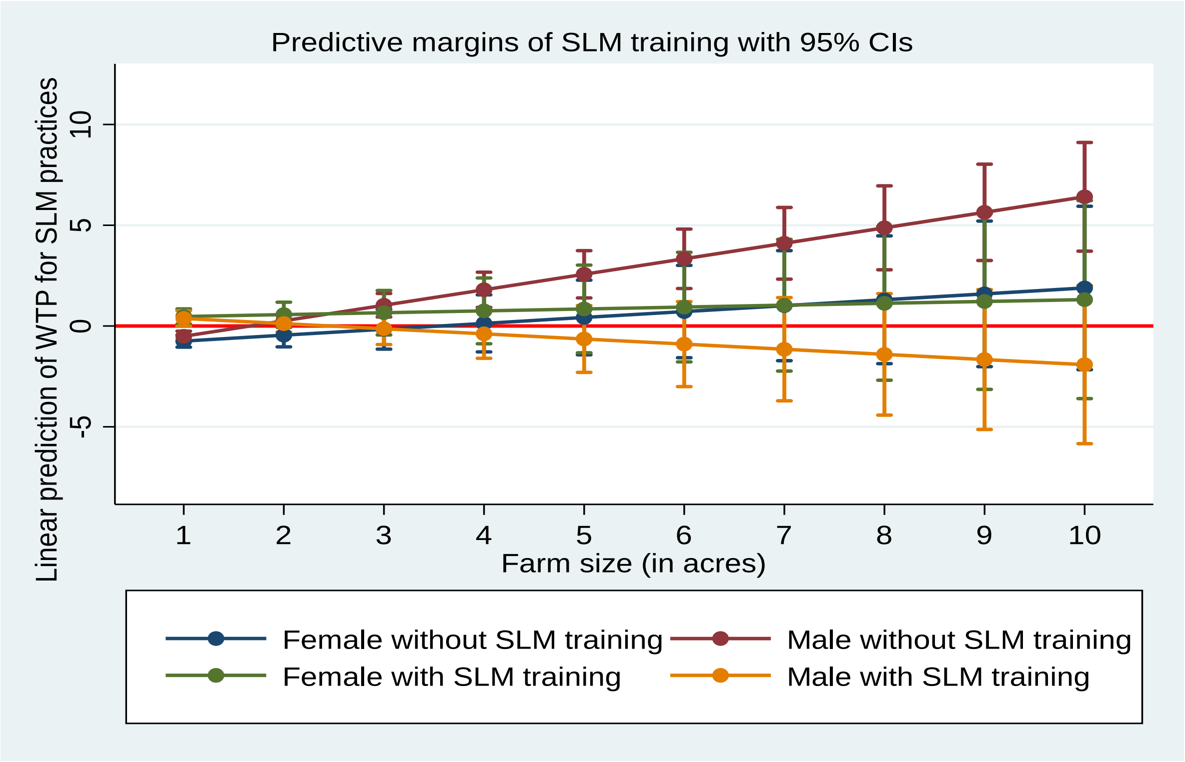

Fig 2: Predictive margins of SLM training by gender and farm size

already possess substantial holdings. This pattern may reflect a saturation effect, wherein extra training yields limited benefits for individuals with existing decision-making autonomy and productive capacity. Interestingly, untrained male farmers exhibit a rising WTP trend with increasing farm size, potentially due to unobserved exposure to informal knowledge networks or previous experience with sustainable practices not captured in the training variable. These dynamics are consistent with the findings of [15], who argue that the behavioural impacts of training interventions is most pronounced when foundational constraints, such as limited resources of knowledge gaps, are addressed.

Overall, while SLM training appears less effective—or potentially even counterproductive—among farmers with large landholdings and capital resources, its marginal effects are strongest among resource-constrained groups, especially women and smallholder farmers. This finding reinforces Hypothesis 3 (H3) and highlights the relevance of examining how interventions interact with socio-economic context, rather than relying solely on average treatment effects. Such an intersectional approach offers deeper insights into the conditions under which training interventions are most impactful.

Other covariates and WTP for SLM practices

Several covariates offer extra insights into the factors influencing farmers' willingness to pay (WTP) for Sustainable Land Management (SLM) practices. Income exerts a positive and statistically significant effect on WTP ($\beta = 0.046$, $p < 0.05$), indicating that as household income increases, farmers may become more risk-tolerant and financially capable of investing in environmental improvement. This finding is consistent with the "worth effect" argued in the Human Capital Theory [13] and is supported by research in environmental economics [18].

However, residing in the coastal zone has a negative and statistically significant association with WTP for SLM practice ($\beta = –0.169$, $p < 0.01$), suggesting that coastal farmers

are less willing to pay for SLM practices than their inland counterparts. These may be attributed to differences in livelihood strategies (i.e., greater reliance on fishing or trading), ecological conditions, or land tenure arrangements. In addition, because land degradation may be perceived as less severe in coastal areas, farmers may not prioritise land conservation.

Household dependency ratios also negatively affect WTP. The number of dependants in the households has a statistically significant negative effect (β = –0.073, $p < 0.05$), plausibly reflecting resource constraints or competing household needs. This is consistent with studies such as [10, 27], which show that households with more dependants often experience reduced capacity to invest in environmental public goods.

The effects of education on WTP are more complex. While primary education and other lower levels show no statistically significant effect, vocational education is negatively and statistically significantly associated with WTP (β = –0.193, $p < 0.05$). This may show the opportunity costs of environmental investment for individuals with alternative income-generating skills or less agricultural dependence. These findings resonate with [14], who argue that general education may not promote pro-environmental behaviours unless it explicitly incorporates environmental content.

Conclusions

This study investigated the gendered effects of Sustainable Land Management (SLM) training and farm size on farmers' willingness to pay (WTP) for SLM practices, using a Tobit regression analysis based on data from five municipalities in Ghana's Volta Region. The results show that both SLM training and farm size have positive and statistically significant effects on WTP, while gender moderates these effects in nuanced ways. Specifically, although male farmers benefit more from SLM training on average, female farmers—particularly those with larger landholdings—exhibit marginal increases in WTP as farm size increases. Additionally, several

contextual variables, including residence in coastal areas, higher household dependency ratios, lower income, and vocational education, are associated with WTP. These findings underscore the complex interplay between gender, access to productive resources, and knowledge acquisition in shaping environmental behaviour. They align with Human Capital Theory (HCT), which highlights the productivity-enhancing effects of training, and Behavioural Economics, which underscores the influence of social norms, financial constraints, and perceived risks on decision-making.

From a policy perspective, the findings highlight the significance of gender-responsive SLM programmes that promote equitable access to training, financing, and land ownership. Interventions such as microcredit schemes, inclusive extension services, and gender-based SLM training modules should prioritise female farmers with land access and male farmers with smaller landholdings, where the marginal benefits of training are greatest. The estimated WTP values also provide valuable inputs into participatory land valuation frameworks, as they reveal the perceived economic value of environmental services delivered through SLM practices.

The study has certain limitations. The cross-sectional design limits causal inference. While the results are robust across Tobit, Two-part, and Heckman models— indicating that the observed relationships are not model-specific—future research should consider instrumental variable Tobit models or panel data approaches to address unobserved heterogeneity and potential reverse causality. Such extensions would enhance the credibility and policy relevance of causal claims.

Acknowledgements

The authors are grateful to the editors and anonymous reviewers for their insightful comments and valuable time, which have significantly improved the quality of this paper.

References

1. Government of Ghana. Ministry of Local Government and Rural Development [Internet]. Ghana, 2016. Available from: http://www.ghanadistricts.com
2. Jinbaani AN. Sustainable land management in the Upper West region of Ghana: a forgotten solution to land degradation, communication failure or a non-priority option? [final project]. Reykjavik: United Nations University Land Restoration Training Programme; 2016 [cited 2022 Nov 4]. Available from: http://www.unulrt.is/static/fellows/document/jinbaani2016.pdf
3. Diao X, Sarpong DB. Poverty implications of agricultural land degradation in Ghana: an economy-wide, multimarket model assessment. African Development Review. 2011 Sep;23(3):263-75. https://doi.org/10.1111/j.1467-8268.2011.00285.x
4. McElwee P, Calvin K, Campbell D, Cherubini F, Grassi G, Korotkov V, Le Hoang A, Lwasa S, Nkem J, Nkonya E, Saigusa N. The impact of interventions in the global land and agri-food sectors on Nature's Contributions to People and the UN Sustainable Development Goals. Global Change Biology. 2020 Sep;26(9):4691-721. https://doi.org/10.1111/gcb.15219
5. Matlou R, Bahta YT, Owusu-Sekyere E, Jordaan H. Impact of agricultural drought resilience on the welfare of smallholder livestock farming households in the Northern Cape Province of South Africa. Land. 2021 May 27;10(6):562. https://doi.org/10.3390/land10060562
6. Ali EB, Agyekum EB, Adadi P. Agriculture for sustainable development: A SWOT-AHP assessment of Ghana's planting for food and jobs initiative. Sustainability. 2021 Jan 11;13(2):628. https://doi.org/10.3390/su13020628
7. Takele MT, Umer MI. Determinant Factors of Farmers' Willingness to Pay for Sustainable Land Management Practices: Evidence with Homosha Benishangul Gumuz Region:

Application with Multinomial Logit Model, Mixed Logit Model, and Conditional Logit Model. https://doi.org/10.21203/rs.3.rs-56133/v1

8. Alemu GT, Tsunekawa A, Haregeweyn N, Nigussie Z, Tsubo M, Elias A, Ayalew Z, Berihun D, Adgo E, Meshesha DT, Molla D. Smallholder farmers' willingness to pay for sustainable land management practices in the Upper Blue Nile basin, Ethiopia. Environment, Development and Sustainability. 2021 Apr;23(4):5640-65. https://doi.org/10.1007/s10668-020-00835-6
9. Amoah A, Jumah A, Korle K. Exploring willingness-to-pay for the restoration and maintenance of reserved forests in the Greater Accra Region of Ghana. Forest Ecosystems. 2022 Jan 1; 9:100041. https://doi.org/10.1016/j.fecs.2022.100041
10. Ndetewio PI, Mwakaje AG, Mujwahuzi M, Ngana J. Factors influencing willingness to pay for watershed services in lower Moshi, Pangani Basin, Tanzania. http://hdl.handle.net/123456789/1004
11. Quisumbing AR, Meinzen-Dick R, Raney TL, Croppenstedt A, Behrman JA, Peterman A. Closing the knowledge gap on gender in agriculture. In Gender in agriculture: Closing the knowledge gap 2014 Feb 19 (pp. 3-27). Dordrecht: Springer Netherlands. https://doi.org/10.1007/978-94-017-8616-4_1
12. Lee CJ. Gender-sensitivity in land management: trajectory of housing, agriculture and land ownership in South Korea. In Land governance and gender: The tenure-gender nexus in land management and land policy 2021 Dec 6 (pp. 91-99). Wallingford, UK: CABI. https://doi.org/10.1079/9781789247664.0008
13. Becker GS. Human capita. New York: National Bureau of Economic Research. 1964.
14. Burton RJ. The influence of farmer demographic characteristics on environmental behaviour: A review. Journal of Environmental Management. 2014 Mar 15; 135:19-26. https://doi.org/10.1016/j.jenvman.2013.12.005

15. Maharjan KL, Gonzalvo CM. Examining Farmers’ Willingness to Learn Environmental Conservation Agriculture: Implications for Women Farmer Empowerment in Bagmati Province, Nepal. Agriculture. 2025 Mar 28;15(7):726. https://doi.org/10.3390/agriculture15070726
16. Kragt ME, Sarmiento JM, Rola-Rubzen MF. Key behavioural science concepts applied to farmer decision-making and farm risk management. https://doi.org/10.22004/ag.econ.369057
17. Byerly H, Kross SM, Niles MT, Fisher B. Applications of behavioural science to biodiversity management in agricultural landscapes: Conceptual mapping and a California case study. Environmental Monitoring and Assessment. 2021 Apr;193(Suppl 1):270. https://doi.org/10.1007/s10661-020-08815-z
18. Amoah A, Moffatt PG. Willingness to pay for reliable piped water services: evidence from urban Ghana. Environmental Economics and Policy Studies. 2021 Oct;23(4):805-29. https://doi.org/10.1007/s10018-021-00303-z
19. Akaateba MA. The politics of customary land rights transformation in peri-urban Ghana: Powers of exclusion in the era of land commodification. Land Use Policy. 2019 Nov 1; 88:104197. https://doi.org/10.1016/j.landusepol.2019.104197
20. Bonye SZ, Yiridomoh GY, Bebelleh FD. Compliance with land use regulations in peri-urban areas in Ghana: a study of Bamahu and Danko residential areas in Wa municipality, Upper West Region. GeoJournal. 2021 Dec;86(6):2845-59. https://doi.org/10.1007/s10708-020-10229-w
21. Ayano MF. Understanding the Local Complexities in Land Law Reforms: The Case of Land Inalienability in Ethiopia, 1991–2018. Law & Social Inquiry. 2024 Nov;49(4):2398-438. https://doi.org/10.1017/lsi.2024.28

22. Asamoah M, Owusu-Ansah F. Report on land tenure and cocoa production in Ghana. Cocoa Research Institute of Ghana and World Cocoa Foundation: Accra, Ghana. 2017 Feb.

23. Lambongang M, Ansah IG, Donkoh SA. Participation and yield effect of Ghana's planting for food and jobs programme in Bunkpurugu-Yunyoo district. Ghana Journal of Agricultural Economics and Agribusiness Vol. 2019 Jun;2(1).

24. Kidido JK, Bugri JT. Youth access to agricultural land from a peri-urban and rural perspective: a study of Techiman traditional area in Ghana. Journal of Land and Rural Studies. 2020 Jan;8(1):37-56. https://doi.org/10.1177/2321024919883145

25. Legesse W, Haji J, Ketema M, Emana B. Determinants of adoption of sustainable land management practice choices among smallholder farmers in Abay Basin of Oromia, Ethiopia. Journal of Development and Agricultural Economics. 2021 Mar;13(1):1-9. https://doi.org/10.5897/JDAE2020.1183

26. Abdeta D. Households' willingness to pay for forest conservation in Ethiopia: A review. Journal of Forest Science. 2022;68(11):437. https://doi.org/10.17221/94/2022-JFS

27. Fakayode SB, Ogunlade I, Ayinde O, Olabode P. Factors affecting farmers' ability to pay for irrigation facilities in Nigeria: the case of Oshin Irrigation Scheme in Kwara State. Journal of Sustainable Development in Africa. 2010 Dec 1;12(1):334-50.

28. Shepler R, Suter JF, Manning DT, Goemans C. Private Actions and Preferences for Coordinated Groundwater Conservation in Colorado's Republican River Basin. JAWRA Journal of the American Water Resources Association. 2019 Jun;55(3):657-69. https://doi.org/10.1111/1752-1688.12741

29. Tang Z, Nan Z, Liu J. The willingness to pay for irrigation water: A case study in Northwest China. Global Nest Journal. 2013 Mar 1;15(1):76-84.

30. Furlong M. ‘Clear at a distance, jumbled up close’: observation, immersion and reflection in the process that is creative research.
31. Krejcie RV, Morgan DW. Sample size determination table. Educational and Psychological Measurement. 1970;30(3):607-10.
32. Cochran WG. Sampling techniques. Johan Wiley & Sons Inc. 1977.
33. Tobin J. Estimation of relationships for limited dependent variables. Econometrica: Journal of the Econometric Society. 1958 Jan 1:24-36. https://doi.org/10.2307/1907382
34. Loomis JB. Balancing public trust resources of Mono Lake and Los Angeles' water rights: An economic approach. Water Resources Research. 1987 Aug;23(8):1449-56. https://doi.org/10.1029/WR023i008p01449
35. Cameron AC, Trivedi PK. Microeconometrics using Stata, Volume I: Cross-sectional and panel regression models. Stata Press; 2022.
36. Long JS, Freese J. Predicted probabilities for count models. The Stata Journal. 2001 Nov;1(1):51-7. https://doi.org/10.1177/1536867X0100100103
37. Kramer M, Weldon PJ. Constructing composite scores for contemporaneous behaviours: A comparison of four approaches. Ethology. 2023 Sep;129(9):489-97. https://doi.org/10.1111/eth.13382
38. Fiagborlo JD. Essays on transport mode choice, fuel and ICT expenditures in Ghana (Doctoral dissertation, University of Cape Coast).
39. Hair JF, Black WC, Babin BJ, Anderson RE, Black WC, Anderson RE. Multivariate data analysis (Eighth Edi). Hampshire, United Kingdom: Cengage Learning EMEA. https://doi. org/10.1002/9781119409137. ch4. 2019.
40. Watkins MW. Exploratory factor analysis: A guide to best practice. Journal of black psychology. 2018 Apr;44(3):219-46. https://doi.org/10.1177/0095798418771807
41. Asante BK. The economy is not healthy: whose fault? Daily Graphic. 2013;(19214):7.

42. Whittington D. Ethical issues with contingent valuation surveys in developing countries: A note on informed consent and other concerns. Environmental and Resource Economics. 2004 Aug;28(4):507-15. https://doi.org/10.1023/B:EARE.0000036776.89379.4f

43. Hansen BB, Pedersen ÅØ, Peeters B, Le Moullec M, Albon SD, Herfindal I, Sæther BE, Grøtan V, Aanes R. Spatial heterogeneity in climate change effects decouples the long-term dynamics of wild reindeer populations in the high Arctic. Global Change Biology. 2019 Nov;25(11):3656-68. https://doi.org/10.1111/gcb.14761

44. Malone HE, Coyne I. Complementing the P-value from null-hypothesis significance testing with a Bayes factor from null-hypothesis Bayesian testing. Nurse Researcher. 2020 Dec 16;28(4). https://doi.org/10.7748/nr.2020.e1756

45. Sribney W. Chi-squared test for models estimated with robust standard errors. StataCorp, August. http://www. stata. com/support/faqs/stat/chi2. html. 1997.

Appendix

Table 6: Heckman selection model as a robustness check for baseline Tobit model

Variables	Tobit model	Heckman eqn. 1	Heckman eqn. 2
Training (SLMT)	0.164***		0.113***
	(0.029)		(0.0325)
Log of farm size (lfsize)	0.058***	0.0858***	–0.176
	(0.020)	(0.0213)	(0.111)
Gender (Male)	0.030	–0.0321	0.0917*
	(0.027)	(0.0314)	(0.0485)
SLMT x lfsize	0.414**	-	0.548***
	(0.142)	-	(0.029)
SLMT x Male	0.315*	-	0.512***
	(0.174)	-	(0.035)
Lfsize x Male	–0.167	0.329***	0.459***
	(0.110)	(0.020)	(0.021)
SLMT x Male x lfsize	0.375**	-	0.522***
	(0.196)	-	(0.039)
Coastal	–0.169***	–0.142***	0.184
	(0.028)	(0.0292)	(0.177)
Log of income	0.046**	0.0372*	–0.0652
	(0.019)	(0.0225)	(0.0508)
Log of experience	–0.001	0.0123	-
	(0.016)	(0.0180)	-
Household dependant	–0.066**	0.00865	–0.108***
	(0.026)	(0.0273)	(0.0295)
Primary education	0.043	0.0347	–0.0884
	(0.049)	(0.0569)	(0.0688)
Middle sch. education	0.049	0.0409	–0.0694
	(0.043)	(0.0511)	(0.0685)
Secondary education	–0.008	0.151**	–0.498***
	(0.064)	(0.0646)	(0.185)
Vocational education	–0.193**	0.110	–0.520***
	(0.083)	(0.0783)	(0.154)
Tertiary education	–0.050	0.190**	–0.536**
	(0.076)	(0.0895)	(0.227)
Inverse mills ratio (IMR)			–1.411**
Observations	1,036	1,036	1,036

Standard errors in parentheses *** p<0.01, ** p<0.05, * p<0.1

Click here to access/download
Supporting Information
PlosOne.xls

Gendered dimensions of sustainable land management: evidence of farm size and training effects on willingness to pay in Ghana's Volta Region

Authors List

James Dickson Fiagborlo[1*], Mexoese Nyatuame[2] and Stella Ahone Ntoko[3]

[1] Department of Multidisciplinary Studies, Faculty of Applied Social Sciences, Ho Technical University, Ho, Ghana (jfiagborlo@htu.edu.gh (J.D. Fiagborlo)); ORCID: 0000-0002-3915-2939

[2] Department of Agric Engineering, Faculty of Engineering, Ho Technical University, Ho, Ghana (mnyatuame@htu.edu.gh (M. Nyatuame)); ORCID: 0000-0001-7652-3308

[3] Centre for Research in Efficient Agricultural Technologies (CREAT), Ho Technical University, Ho, Ghana (nstella15@gmail.com (S.A. Ntoko))

[*] Corresponding Author: jfiagborlo@htu.edu.gh (JDF)

Author Contribution

All authors contributed to the conception and design of the study. Material preparation, data collection, and analysis were conducted by **James Dickson Fiagborlo**, **Mexoese Nyatuame**, and **Stella Ahone Ntoko**. The initial draft of the manuscript was prepared by **James Dickson Fiagborlo**, and all authors read and approved the first and final versions of the manuscript.

Abstract

Land degradation continues to pose a significant threat to agricultural productivity in Ghana, eroding soil fertility and exacerbating rural poverty. Although efforts have been made to promote Sustainable Land Management (SLM), adoption remains limited among smallholder farmers—particularly women—due to persistent disparities in land ownership, training access, and decision-making autonomy. Drawing on survey data from 1,036 farm households across five municipalities in Ghana's Volta Region, this study investigates the gendered effects of SLM training and farm size on farmers' willingness to pay (WTP) for SLM practices. A Tobit regression model is employed to estimate both main and interaction effects. The results suggest that both SLM training ($\beta = 0.164$, $p < 0.01$) and farm size ($\beta = 0.058$, $p < 0.01$) significantly increase WTP, highlighting the roles of knowledge acquisition and resource endowment in promoting environmental investment. Significantly, gender moderated these effects: while male farmers initially benefited more from training, female farmers with larger landholdings exhibited higher marginal increases in WTP, suggesting that access to land enhances women's capacity to invest in SLM. Additionally, WTP is negatively associated with coastal residence, larger household dependency ratios, lower income, and vocational education—factors that reflect broader livelihood and financial constraints. These findings highlight the significance of gender-sensitive SLM interventions that combine equitable access to training with improved access to productive resources. Such targeted strategies aim to increase smallholder participation in sustainable land management and address the socio-economic drivers of land degradation in Ghana.

Keywords: Sustainable land management. Willingness to pay. Gender. Farm size. Training. Ghana.

Introduction

The agricultural landscape in Ghana is facing severe threats from deforestation, overgrazing, and uncontrolled burning, resulting in large-scale land degradation and loss of productivity. An estimated 69% of the agricultural land in Ghana is already eroded, costing the economy an estimated US$4.2 billion annually—equivalent to 5% of agricultural GDP [1, 2]. Land degradation is deepening poverty by reducing soil fertility, lowering yields, and limiting the use of basic inputs such as fertiliser, irrigation, and mechanised tools by farmers [3]. In response to these challenges, there has been a renewed emphasis on Sustainable Land Management (SLM) and Agricultural Sustainable Land Management (ASLM) as key vehicles for restoring soil health and enhancing agricultural resilience [4]. According to TerrAfrica, SLM is "adopting land-use systems that enhance ecological support functions through appropriate management techniques." [5] broaden this definition to cover the role of ASLM in improving the yields of crops, livestock, watersheds, and forests. In this study, SLM and ASLM are used interchangeably.

Notwithstanding policy interest, the adoption of Sustainable Land Management (SLM) practices remains limited in Ghana. Poor financial conditions, inadequate extension support, and erratic access to the requisite technologies have impeded uptake, particularly among smallholder farmers in the Volta Region [6, 7]. Beyond these fundamental barriers, social and behavioural dimensions—especially gender differences in access to training, land, and decision-making power—play a crucial role in shaping adoption behaviour. For instance, studies [8–10] have shown that farmers' willingness to pay (WTP) for environmental and sustainable practices is influenced by socioeconomic factors such as gender, age, education, income, and farm size. While considerable evidence from other developing regions shows that women face disproportionate barriers to land ownership, credit, and training [11, 12], the gendered subtleties of SLM adoption and WTP remain understudied in the context of Ghana.

In theoretical terms, the differences between men and women in land and resource management can be understood through Human Capital Theory [13] and the Gender and Agricultural Decision-Making Framework [11]. These frameworks demonstrate that unequal access to knowledge, skills, and productive assets leads to unequal economic returns. This is indeed the case: land tenure for women is limited, and their access to training and inputs is restricted, ultimately constraining their adoption of, or investment in, Sustainable Land Management (SLM) [14, 15]. This suggests that women's willingness to pay (WTP) is likely to be less influenced by resource endowment and more by social norms, perceived risk, and intra-household decision-making power. Evidence from behavioural studies supports this view and suggests that agricultural decisions are shaped not only by resource availability but also by perceived efficacy and social learning [16, 17].

Empirical studies from Africa [8, 10] and Asia [12, 15] further confirm the importance of gender in sustainable agricultural practices. In Ethiopia, [8] reported that male-headed households were more likely to invest in Sustainable Land Management (SLM), due to greater land ownership and better access to extension services. In Tanzania, for example, [10] shows that women's willingness to pay for watershed services was significantly lower, reflecting gender-based financial barriers. By contrast, [15] reported that female farmers' willingness to participate in environmental conservation programmes increased when training was explicitly designed to empower female participants. Similarly, [12] states that gender-sensitive land governance frameworks in South Korea have empowered women to gain tenure rights and participate fully in land-use decisions. Such findings collectively point to the need for a study into how training, access to land, and gender roles interact to influence SLM-related decisions.

The interaction of Sustainable Land Management (SLM) training with farm size and gender is poorly understood in Ghana. While the aim of such training is to enhance farmers' technical and management knowledge [18], its impacts vary by gender due to fundamental

inequalities in land access and social capital. For example, male farmers may leverage larger farm sizes and greater financial independence to apply knowledge gained through training, whereas females may be constrained, even when exposed to similar training opportunities. Understanding the gendered effects of training and farm size on willingness to pay (WTP) for SLM practices is therefore key to designing equitable agricultural interventions.

The study addresses gaps in the existing literature by examining the gendered effects of Sustainable Land Management (SLM) training and farm size on farmers' willingness to pay (WTP) for SLM practices in Ghana's Volta Region. Specifically, it aims to: (1) identify the effect of SLM training on WTP for SLM practices among male and female farmers; (2) explore the impact of farm size on WTP for SLM practices across genders; and (3) assess the gendered dimensions of overall WTP for SLM practices.

This study contributes to the growing literature on gender and sustainable agriculture in three key ways. First, it provides empirical evidence on how training and resource endowments (farm size) interact with gender to shape sustainability-oriented behaviour. Second, through the application of a gender-sensitive analytical framework, it enriches the literature on environmental economics in the context of valuing Sustainable Land Management (SLM) practices. Third, it generates policy-relevant insights to support gender-equitable land management and climate adaptation strategies.

The remainder of the paper is structured as follows: Section 2 presents a review of the literature on Sustainable Land Management and land policy in Ghana; Section 3 outlines the methodological framework; Section 4 presents and discusses the empirical results; and Section 5 concludes with policy implications, limitations, and recommendations for future research.

Literature review

This section reviews land management policies in Ghana and synthesises empirical evidence on Sustainable Land Management practices, training, farm size, gender, and farmers' willingness to pay. It draws on both African and global contexts to provide a comprehensive foundation for the development of the hypotheses of this study.

Land management techniques and policies in Ghana

Ghana's National Land Policy, enacted in 1999, seeks to establish an efficient legal and administrative framework for the management of land rights, to recognise traditional authorities, and to ensure the sustainable use of land in the context of a growing population and increasing land-use pressures [19, 20]. The policy addresses challenges such as unregulated land markets, multiple sales, encroachment, and unplanned development. Its major goals are to ensure tenure security, enhance productivity, and promote sustainable land use [1, 19].

Implementation strategies include the preparation of national-and district-level land-use plans, the formation of inter-ministerial technical groups for dispute resolution, and, importantly, inclusive decision-making involving all relevant stakeholders [21]. The policy also prohibits forest clearing for mining purposes, based on the premise that sound land-use planning can reduce conflicts and improve ecological outcomes.

Other complementary frameworks include the Forest and Wildlife Policy, Fire Policy, and the Sustainable Land Management Policy. These were also introduced with a view to enhancing integration of natural resource management in agriculture [5]. However, their implementation faces challenges characterised by weak institutional capacity, limited enforcement, and poor coordination among stakeholders [22]. Farmers continue to engage in

unsustainable practices such as slash-and-burn cultivation, driven by economic necessity and/or a lack of awareness about the long-term effects of land degradation [23].

The implementation of the Food and Agriculture Sector Development Policy (FASDEP), the Northern Rural Growth Programme, and the Ghana Strategic Investment Fund (GSIF) has advanced Sustainable Land Management initiatives. However, inadequate community participation and gender-insensitive approaches have generally constrained the impact achieved thus far by these programmes [24]. A growing body of research calls for multi-stakeholder, gender-inclusive land governance models that integrate the voices of farmers, extension officers, and local institutions in ways that promote long-term sustainability [6].

Empirical literature on Sustainable Land Management and gender

Empirical research across the African continent confirms that Sustainable Land Management practices, including conservation agriculture, agroforestry, and integrated soil fertility management, improve yields, enhance soil health, and strengthen climate resilience [4, 25]. However, adoption rates remain low, particularly among smallholder farmers, due to limited access to finance, inadequate extension services, and weak policy support [7]. Economies of scale, access to credit, and lower transaction costs typically lead larger farms to exhibit higher adoption rates [8]. Nevertheless, smallholder farmers who receive training also demonstrate greater uptake of conservation practices [25].

Training is generally regarded as a key determinant of Sustainable Land Management (SLM) adoption and willingness to pay (WTP), as it improves farmers' understanding of sustainable practices and enhances their perception of long-term benefits associated with these interventions [10, 26]. However, training opportunities often remain gendered. In many rural African contexts, men are more likely to attend agricultural training sessions or receive extension services due to social norms, greater time flexibility, and fewer restrictions on

mobility [11]. For example, studies from Ethiopia [8] and Tanzania [10] report that female farmers exhibit lower WTP for SLM-related services, not due to disinterest, but rather as a result of structural inequalities in access to resources.

Recent studies further underscore the intersectional relationship between gender, access to resources, and environmental decision-making across Sub-Saharan Africa and Asia. For instance, [15] observed that in Nepal, women's willingness to participate in conservation agriculture significantly increased when training was tailored to their empowerment. Similarly, [12] noted that a gender-sensitive land governance framework in South Korea improved women's tenure security and participation in sustainable land decisions. However, evidence from Ethiopia and Kenya indicates that male-headed households remain more likely to adopt capital-intensive Sustainable Land Management practices, largely due to their greater access to credit and mechanisation facilities [14, 25].

Gender differences in willingness to pay and adoption behaviour can also be analysed through the lens of behavioural science. According to [16] and [17], risk perceptions, trust, and social learning are major drivers of farmers' environmental decisions. In particular, women's incentives for participating in SLM initiatives are often shaped by community networks and collective learning prospects, rather than by individual resource endowments. This highlights the need for both material and social responses to foster truly gender-equitable participation—addressing disparities in access to resources as well as differences in behavioural motivations.

Farm size, SLM training, gender, and willingness to pay

Farm size remains a critical factor affecting willingness to pay (WTP). Larger farmers can spread the fixed costs of adoption over greater acreage, thereby realising scale efficiencies [25]. Empirical evidence from Nigeria [27], Egypt [28], and China [29] indicates that farm size and income are significant predictors of higher WTP for irrigation and soil management practices.

However, studies also reveal that beyond a certain threshold, larger farms may experience diminishing marginal returns to investment in Sustainable Land Management practices [8].

Training interacts with farm size in shaping adoption outcomes, as larger farmers are typically better positioned to apply knowledge gained from Sustainable Land Management training, while smaller farmers may struggle to translate knowledge into practice due to financial constraints [25]. Gender further moderates this interaction; female farmers, even when trained, may not fully benefit from training if they do not own land or lack access to complementary inputs such as credit or tools [11, 15].

Summary of literature review and hypotheses development

Although previous studies provide valuable insights into Sustainable Land Management (SLM) adoption and willingness to pay (WTP), several critical gaps persist. First, there is a limited understanding of how SLM training, farm size, and gender interact to shape farmers' WTP for SLM practices. Second, the gendered dimensions of WTP—including differences in decision-making power, access to credit, and exposure to training —remain underexplored in Ghana. Third, literature rarely integrates behavioural perspectives that explain how risk perception and social norms influence WTP differently for male and female farmers. To address these gaps, this study investigates how gender moderates the effects of SLM training and farm size on farmers' WTP for SLM practices in Ghana's Volta Region. Guided by the theoretical and empirical literature, the following hypotheses are proposed:

H1: Farmers who received SML training will demonstrate higher WTP for SLM practices than those who have not.

H2: Larger farm sizes are associated with higher WTP for SLM practices.

H3: Female farmers with equitable access to training and resources will exhibit higher WTP for SLM practices than male farmers.

This gender-focused framework recognises that SLM decisions are shaped not only by economic capacity but also by institutional, behavioural, and social dynamics.

Materials and methods

Study design and sampling procedure

This study investigates gendered dimensions of Sustainable Land Management (SLM) training, farm size, and willingness to pay (WTP) for SLM practices among farming households in Ghana's Volta Region. A multistage sampling technique was employed to ensure balanced representation across ecological and administrative clusters. In the first stage, five municipalities were purposively selected to capture the region's major ecological zones. Within each municipality, five communities were randomly selected, yielding a total of 25 study sites. In the second stage, 50 farm households were randomly selected from each community using a simple random sampling technique, resulting in a total sample of 1,250 households. This stratified design accounts for potential heterogeneity across localities—such as variation in rainfall, soil fertility, and access to agricultural extension services—thereby enhancing representativeness and guaranteeing statistical reliability.

Due to missing information on some variables, the final sample was reduced to 1036 households. Following [30], this sample size was deemed adequate to achieve the study's objectives. Moreover, it exceeds the minimum thresholds recommended by [31] and [32] for a 95% confidence level, thereby ensuring statistical adequacy and analytical robustness. This research involved structured interviews with adult members of farming households. Prior to participation, all prospective respondents were provided with a detailed explanation of the study's purpose, its voluntary nature, and their right to decline participation or withdraw at any

time without any negative consequences. Informed verbal consent was obtained from each participant before the interview commenced.

Given the literacy levels in some rural areas and the cultural context of the fieldwork, verbal consent was deemed appropriate and ethically justified. Trained enumerators communicated in either English or Ewe using a standardised script to ensure full understanding. Each instance of verbal consent was recorded in the survey logbook maintained by field team supervisors. Only adult members of the household (aged 18 years and above) were interviewed; minors were excluded from the study. Although formal institutional ethical clearance was not obtained prior to the 2016 fieldwork, all data were fully anonymised, and strict confidentiality procedures were followed. No personally identifying material was collected, and all responses were securely stored and used solely for research purposes. The research procedure adhered to standard ethical practices for social science fieldwork in Ghana at the time of data collection.

Data, description, and measurement of variables

The questionnaire collected detailed socio-demographic and farm-level information necessary to analyse the factors impacting farmers' willingness to pay for Sustainable Land Management (SLM) practices. Respondents provided data on age, gender, education, income, farming experience, number of household dependants, farm size, and participation in SLM training programmes. A community location variable (coastal vs. inland) was also included to control for geographic and environmental heterogeneity.

The dependent variable, willingness to pay (WTP), was elicited through a contingent valuation (CV) question, framed as follows: *"SLM practices serve multiple functions: they prevent soil erosion, enhance water retention, support groundwater recharge, and mitigate flooding. In view of these benefits, what is the maximum amount in Ghanaian Cedis (GHS) you are willing to pay to sustain the environmental benefits provided by SLM practices?"*

Respondents who reported zero WTP were classified as non-payers, while positive responses were recorded as the maximum amount they were willing to pay.

Table 1 summarises the measurement of all variables used in the analysis. The key explanatory variables include Sustainable Land Management (SLM) training, a binary variable indicating whether the respondent has received any form of SLM-related training, and farm size, measured as the total land area (in acres) cultivated by the farmer. Gender is coded as a binary variable, where 1 represents male respondents and 0 represents female respondents. To isolate the impacts of socio-economic characteristics on willingness to pay (WTP), several control variables are included: household income, years of farming experience, number of household dependants, and education. Household income is measured as a continuous variable

Table 1. Measurement of variables

Variable	Measurement	Min	Max
**WTP**	Continuous: non-negative censored variable (GHS)	0	500
**SLM training**	Binary: 1 = farmer received SLM training; 0 = otherwise	0	1
**Farm size**	Continuous: farm size in acres	1	45
**Male**	Binary: 1= male farmer; 0 = otherwise	0	1
**Coastal**	Binary: 1 = coastal resident; 0 = otherwise	0	1
**Income**	Continuous: monthly income (GHS)	30	1000
**Household dependant**	Continuous: number of household dependants	1	18
**Farmer experience**	Continuous: years of farming experience	1	60
**No education**	Binary: 1 = no formal education; 0 = otherwise	0	1
**Primary education**	Binary: 1 = primary education; 0 = otherwise	0	1
**Middle sch. education**	Binary: 1 = middle sch. education; 0 = otherwise	0	1
**Secondary education**	Binary: 1 = secondary education; 0 = otherwise	0	1
**Vocational education**	Binary: 1 = vocational training; 0 = otherwise	0	1
**Tertiary education**	Binary: 1 = tertiary education; 0 = otherwise	0	1

NB: Max= Maximum; Min= Minimum

capturing monthly earnings. Farming experience, measured in years, reflects accumulated agricultural knowledge, which may influence risk perception and investment behaviour.

Education is represented by categorical (dummy) variables indicating different levels of attainment, ranging from no formal education to tertiary-level qualifications. The minimum and maximum values presented in Table 1 provide a comprehensive overview of variation within the sample and support the replicability of the empirical analysis.

Econometric model

Given that willingness to pay (WTP) is a non-negative continuous variable censored at zero, a Tobit regression model estimated via Maximum Likelihood Estimation was employed [33]. This modelling approach is appropriate when the dependent variable is observed only above or at a certain threshold, in this case, zero, and it accommodates both the probability of being above the threshold and the conditional mean of the uncensored observations. Previous studies have employed similar approaches to estimate WTP and other censored outcomes, noting the Tobit model's ability to account for the limited range of values while preserving estimation efficiency [34,35]. The empirical model is specified as follows:

$$
\begin{aligned}
WTP^*_i = {} & \beta_0 + \beta_1 SLMT_i + \beta_2 FAMS_i + \beta_3 GM_i + \beta_4(\mathrm{SLMT_i \ x \ FAMS_i}) + \beta_5(\mathrm{SLMT_i \ x \ GM_i}) \\
& + \beta_6(\mathrm{FAMS_i \ x \ GM_i}) + \beta_7(\mathrm{SLMT_i \ x \ GM_i \ x \ FAMS_i}) + \beta_8 COC_i + \beta_9 INC_i + \beta_{10} HD_i \\
& + \beta_{11} FE_i + \beta_{12} ED_i + \varepsilon_i
\end{aligned}
$$

where WTPi∗ represents the latent (unobserved) willingness to pay. The observed WTP is defined as $\mathrm{WTP_i} = \mathrm{WTP^*_i}$, if $WTP^*_i > 0$ and $WTP_i = 0$, if $WTP^*_i \leq 0$. β s are the coefficients associated with the predictors, and ε_i denotes the stochastic term, which is considered to be independent and normally distributed with a zero mean and constant variance. The set of independent variables includes Sustainable Land Management training (SLMT), farm size (FAMS), gender (GM), coastal location (COC), income (INC), number of household dependants (HD), farming experience (FE), and education level (ED). To examine moderating

effects, the model incorporates interaction terms: SLMT x FAMS, SLMT x GM, GM x FAMS, and SLMT x GM x FAMS. These interactions capture the combined influence of training, farm size, and gender on willingness to pay (WTP) for Sustainable Land Management practices.

As part of the analytical strategy, descriptive statistics were computed to summarise household and farm-level characteristics (Table 3). Following [36], average marginal effects (AMEs) were derived from the Tobit model to provide insight into both the direction and magnitude of relationships among variables. All analyses were conducted using Stata version 15, with robust standard errors applied to address potential heteroskedasticity.

Diagnostic tests

Table 2 presents an assessment of multicollinearity among independent variables using Variance Inflation Factors (VIF) and condition number. The highest VIF—3.03 for the middle school education category—falls well below conventional multicollinearity thresholds of

Table 2: Estimated VIF for the variables used in the analysis

Variable	VIF	SqrtVIF	Toleran	R-squared
**SLM training**	1.08	1.04	0.929	0.071
**Farm size**	1.23	1.11	0.815	0.185
**Gender (Male)**	1.14	1.07	0.879	0.121
**Coastal**	1.17	1.08	0.855	0.145
**Income**	1.18	1.09	0.848	0.152
**Farmer experience**	1.08	1.04	0.927	0.073
**Household dependant**	1.06	1.03	0.948	0.052
**Primary education**	2.32	1.52	0.431	0.569
**Middle school education**	3.03	1.74	0.330	0.670
**Secondary education**	1.92	1.39	0.520	0.480
**Vocational education**	1.44	1.20	0.696	0.304
**Tertiary education**	1.45	1.20	0.691	0.309
**Mean VIF**			1.51	
**Breusch-Pagan/Cook-Weisberg test for heteroskedasticity**				
**Chi^2**			28.36	
**Prob> Chi^2**			0.000	

greater than 5 or 10 [35], indicating no serious collinearity issues. Additionally, the condition number (12.5) and the overall mean VIF (1.51) are below the generally accepted threshold of 15, further suggesting that multicollinearity is not a concern [37–40]. The sample size of 1036 exceeds the minimum threshold that [31] recommended, ensuring statistical adequacy and model robustness. To test for heteroskedasticity, the Breusch-Pagan or Cook-Weisberg test was conducted (Table 2). The test results indicated statistically significant heteroskedasticity, suggesting that the variance of the error terms is not constant across observations. Accordingly, robust standard errors were applied in all the regression analyses to correct for this violation and to ensure valid statistical inference. Overall, the suitability of the Tobit model is supported by these diagnostic checks and confirmed by a combination of robustness checks (Table 5), which provide confidence in the model estimates and justify the appropriateness of using the Tobit specification. The next section presents the summary statistics and empirical results.

Results and discussions

Table 3 presents the descriptive statistics of the variables used in the analysis. The average stated willingness to pay (WTP) for Sustainable Land Management (SLM) practices across all respondents is GHS 76.59 (US$19.15)[1]. While this mean value covers all survey participants, it is important to indicate that the median WTP is GHS 0. This implies that more than 50% of the farmers reported no WTP, indicating that a relatively small subgroup of farmers is driving the mean up through high stated willingness to pay values. To estimate aggregate WTP, the mean WTP was multiplied by the estimated number of agricultural households. According to the 2010 Population and Housing Census, 888,271 individuals in the Volta Region were engaged in agriculture. Using the national average household size of 4.4, the number of regional agricultural households was estimated to be about 201,879.77. Nationally, with 10.48 million individuals engaged in agriculture, the number of agrarian households was estimated

Table 3. Summary statistics

Variables	Observation	Mean	SD
**Willingness to pay (WTP)**	1036	76.59	118.24
**SLM training**	1036	0.35	0.48
**Farm size**	1036	4.00	3.57
**male**	1036	0.60	0.49
**coastal**	1036	0.54	0.50
**Income**	1036	346.62	207.62
**Household dependant**	1036	4.96	2.40
**Farmer experience**	1036	11.47	10.05
**No education**	1036	0.10	0.30
**Primary education**	1036	0.19	0.39
**Middle school education**	1036	0.52	0.50
**Secondary education**	1036	0.10	0.30
**Vocational education**	1036	0.05	0.21
**Tertiary education**	1036	0.04	0.19

NB: SD means standard deviation

[1]*Exchange rate is US$1 = GHS4—the average daily exchange at the time of data collection (October 2016 to November 2016). This was retrieved from the Bank of Ghana at https:// www. bog. gov.gh/ treasury- and- the- markets/ historical-interbank- fx- rates/*

to be around 2.4 million. Applying the mean WTP to these populations yields an estimated total WTP of GHS15.46 million (US$3.87 million) for the Volta Region and GHS182.41 million (US$45.60 million) for Ghana. These figures offer an indication of the total economic value that farming households place on the environmental benefits of SLM practices.

The average willingness to pay of GHS 76.59 represents approximately 19% of the average monthly farm income, suggesting that farmers who are willing to pay perceive the benefits of Sustainable Land Management as economically meaningful. Conversely, these estimates should be interpreted with caution, as they echo stated preferences under hypothetical conditions. Actual payments in a realistic context may not be the same due to budget constraints, behavioural influences or contextual difficulties.

Descriptive statistics reveal that 35% of farmers had received Sustainable Land Management training, while the majority (54%) lived in coastal areas. The average monthly income was GHS346.62 (US$86.66). Male farmers constituted 60% of the sample (Table 3). On average, farmers had five dependants and 12 years of farming experience. Educational attainment was quite high; only 10% of the farmers had no formal education, while 90% had received some level of education up to the tertiary level. The average farm size was four acres. Farmers' reluctance to pay for Sustainable Land Management (SLM) practices can be attributed to several factors. Approximately 177 farmers (31%) indicated that they believed funding for SLM initiatives should be the responsibility of the government (Fig 1). Another 89 farmers (16%) reported that high farm-related expenditures were already overwhelming them. Moreover, 84 farmers (15%) stated that they would be willing to pay only if they observed

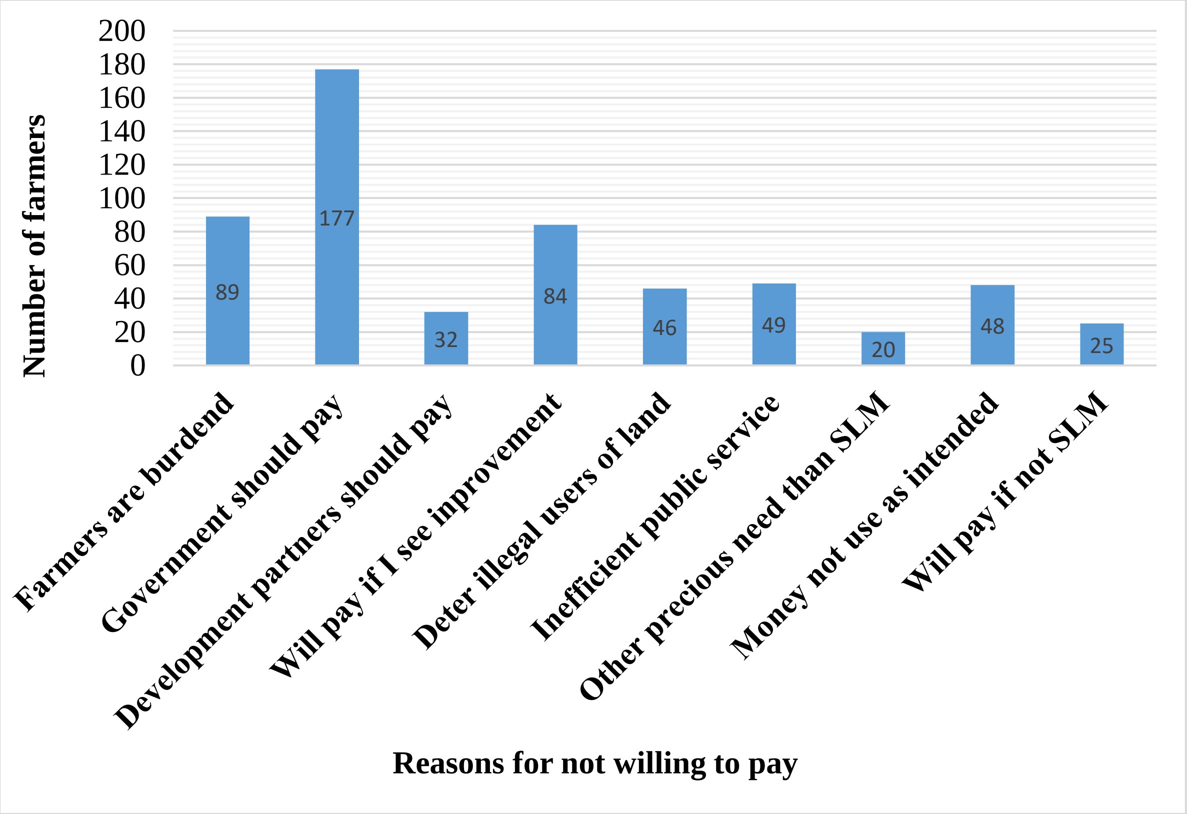

Fig 1: Reasons for not being willing to pay for SLM service

tangible improvements in SLM implementation. A further 49 farmers (9%) conveyed distrust in public institutions, stating that government employees were ineffective and that contributions made by farmers might be pocketed. Additionally, 25 farmers (4%) reported that they would only be willing to pay if SLM activities were not implemented at all, perhaps showing an inherent misunderstanding of SLM goals. These findings suggest the need for policymakers to understand farmers' genuine fears, mainly regarding trust in public officials and the perceived effectiveness of SLM interventions.

Public trust is a critical determinant of willingness to pay, as suggested by empirical data [41]. Over 15% of farmers said that they would consider paying only if SLM services were better. According to the Centre for Environment and Development (CED), unreliable services reduce public confidence and, as a result, willingness to contribute financially. As observed in past studies, confidence in the institutions responsible for managing public funds significantly influences willingness to pay for public goods and services [42]. When individuals believe their donations will be used effectively, resistance to paying declines. On the other hand, unresolved concerns about governance and accountability may suppress WTP and also hinder future efforts to introduce cost-sharing models in environmental management [41].

Empirical results

Table 4 presents the average marginal effects (AMEs) and summary statistics of key predictors on willingness to pay (WTP) for Sustainable Land Management (SLM) practices. A total of eight models were estimated in all. Model 1 reports the baseline Tobit specification. Models 2 and 3 adopt a two-part model technique for robustness: Model 2 represents a logit regression estimating the probability of being a payer (i.e., WTP > 0), while Model 3 presents the ordinary least squares (OLS) regression estimating the level of WTP among those who reported positive values. Models 4 through 7 investigated heterogeneous effects by estimating separate Tobit

models for different sub-samples. In particular, Models 4 and 5 examine gender differences by estimating Tobit regression separately for male and female farmers, while Models 6 and 7 do the same for farmers with low and high levels of farming experience. Additionally, a Heckman selection model was estimated (Appendix Table 6) as an additional robustness check to correct for potential selection bias arising from a non-random decision to state a WTP amount. Robust standard errors are reported in parentheses for all model estimates.

All the models are statistically significant at conventional levels, with the F-statistics ranging from 3.81 to 8.52 and associated p-values of 0.000 [43, 44]. This provides strong evidence against the joint null hypothesis that all coefficients are equal to zero, confirming the overall statistical significance and model stability within the context of the data. The pseudo-R^2 values, which range between 0.05 and 0.10, indicate that the models explain approximately 5% to 10% of the variations in the dependent variable. While these values appear low, they are not uncommon in models based on limited dependent variables, such as Tobit or logit models, where pseudo-R^2 does not carry the same interpretation as R^2 in linear regression. As noted in the literature, pseudo-R^2 values can be sensitive to model specification and may fall outside the 0–1 range in certain likelihood-based models [45]. Nevertheless, they provide a useful, albeit approximate, measure of model fit.

Table 4: Average marginal effects of predictor variables on WTP for SLM practices

Variables	Model 1	Model 2	Model 3	Model 4	Model 5	Model 6	Model 7
**Training (SLMT)**	0.164***	0.116***	0.537***	0.107***	0.282***	0.145***	0.170***
	(0.029)	(0.033)	(0.118)	(0.038)	(0.044)	(0.042)	(0.041)
**Log of farm size(lfsize)**	0.058***	0.074***	-0.011	0.054**	0.067**	0.111***	0.021
	(0.020)	(0.022)	(0.089)	(0.024)	(0.033)	(0.027)	(0.027)
**Gender (Male)**	0.030	0.007	0.220*	-	-	0.015	0.036
	(0.027)	(0.032)	(0.114)	-	-	(0.037)	(0.040)
**SLMT x lfsize**	0.414**	0.548***	2.010***	0.310	0.685***	0.378**	0. 468**
	(0.142)	(0.029)	(0.112)	(0.207)	(0.176)	(0.197)	(0.203)

**SLMT x Male**	0.315*	0.512***	2.062***	-	-	0.300	0.291
	(0.174)	(0.035)	(0.133)	-	-	(0.280)	(0.220)
**lfsize x Male**	–0.167	0.459***	1.749***	-	-	–0.117	–0.222
	(0.110)	(0.021)	(0.089)	-	-	(0.156)	(0.156)
**SLMT x Male x lfsize**	0.375**	0.522***	2.089***	-	-	0.290	0. 438*
	(0.196)	(0.038)	(0.157)	-	-	(0.299)	(0.258)
**Coastal**	–0.169***	–0.214***	–0.038	–0.222***	–0.070	–0.208***	–0.123***
	(0.028)	(0.029)	(0.128)	(0.035)	(0.044)	(0.038)	(0.040)
**Log of income**	0.046**	0.039*	0.121	0.065**	0.004	0.035	0.065**
	(0.019)	(0.023)	(0.077)	(0.026)	(0.028)	(0.025)	(0.030)
**Log of experience**	–0.001	–0.009	0.036	0.007	–0.016	-	-
	(0.016)	(0.018)	(0.067)	(0.020)	(0.025)	-	-
**Household dependant**	–0.066**	–0.080***	0.000	–0.042	–0.085**	–0.082**	–0.033
	(0.026)	(0.028)	(0.115)	(0.034)	(0.038)	(0.036)	(0.036)
**Primary education**	0.043	0.000	0.377*	0.086	0.016	–0.005	0.068
	(0.049)	(0.056)	(0.229)	(0.076)	(0.065)	(0.071)	(0.065)
**Middle sch. education**	0.049	0.034	0.234	0.107	0.009	–0.017	0.090
	(0.043)	(0.049)	(0.201)	(0.067)	(0.059)	(0.063)	(0.055)
**Secondary education**	–0.008	–0.108	0.765***	0.129	–0.354***	–0.113	0.078
	(0.064)	(0.068)	(0.292)	(0.084)	(0.105)	(0.089)	(0.085)
**Vocational education**	–0.193**	–0.244***	0.098	–0.220*	–0.103	–0.143	–0.188*
	(0.083)	(0.090)	(0.322)	(0.127)	(0.113)	(0.123)	(0.107)
**Tertiary education**	–0.050	–0.061	0.060	0.020	–0.172	–0.122	–0.013
	(0.076)	(0.094)	(0.315)	(0.092)	(0.189)	(0.105)	(0.100)
**Observations**	1,036	1,036	472	622	414	504	532
**Log/pseudolikelihood**	–1337.92	–649.052	-	–815.154	–507.002	–651.142	–677.88
**LR /Wald chi^2**	-	114.22	-	-	-	-	-
**F(x, n)**	8.51		3.81	8.59	4.86	5.62	4.60
**Pseudo R^2/R^2**	0.050	0.091	0.10	0.06	0.06	0.06	0.05
**Prob> F**	0.000	0.000	0.000	0.000	0.000	0.000	0.000

Robust standard errors in parentheses * *p < 0.01*, ** *p < 0.05*, * *p < 0.1***

NB: Model 1=baseline model (Tobit); Models 2&3= Two-part model (Logit &OLS) for robustness check; Models 4 &5=gendered subsample model (Tobit); Models 6&7=Farmer experience subsample model (Tobit)

Robustness, heterogeneity, and endogeneity considerations

To strengthen confidence in the baseline results, several robustness and validity checks were conducted. As shown in Table 4, the direction and statistical significance of key predictor variables—particularly SLM training and farm size—remain consistent across alternative model specifications, confirming the robustness of the baseline Tobit estimates. Second, the sub-sample analyses (Models 4-7) reveal heterogeneity in effects across gender and farming experience. While the effects of SLM training and farm size remain positive and statistically significant in all groups, the magnitude of these effects differs. In effect, SLM training has a stronger marginal effect among female and less-experienced farmers, stressing the importance of targeted capacity-building interventions for these groups [16, 25]. Third, the statistically significant Inverse Mills Ratio (IMR = –1.411, $p < 0.05$) in the Heckman model confirms the presence of selection bias in the Tobit estimates, thereby justifying the use of the Heckman method. Significantly, however, the sign and magnitude of the key variables remain stable across models, reinforcing the robustness and internal consistency of the baseline results. The next section presents the results of the baseline Tobit model (Model 1) in greater detail.

Main effects

This section examines the main effects of SLM training, farm size, and gender on farmers' willingness to pay (WTP) for Sustainable Land Management (SLM) practices. In model 1, SLM training emerges as a positive and statistically significant predictor of WTP (β = 0.164, $p < 0.01$), thereby confirming Hypothesis 1 (H1). This finding suggests that, holding farm size constant (i.e., lfsize = 0) and assuming female gender (i.e., male = 0), those who receive SLM training demonstrate a higher WTP for sustainable practices. This result is consistent with previous studies that highlight the role of training in empowering farmers and enhancing the adoption of SLM interventions [8, 15, 25].

Similarly, farm size is a positive and statistically significant predictor of WTP ($\beta = 0.058$, $p < 0.01$), confirming Hypothesis 2 (H2). In the absence of SLM training (SLMT=0) and among female farmers (Male=0), those with larger landholdings demonstrate a greater willingness to pay for SLM practices. This result supports the economies of scale argument [27, 28], whereby larger farms reduce per-unit investment costs, thus making adoption more feasible and attractive. It also aligns with previous studies that identity access to productive resources as a key enabler of sustainable practice adoption [11, 14].

Conversely, gender is a positive but statistically insignificant predictor of WTP for SLM practice (0.030, $p > 0.1$). This suggests that, in the absence of both SLM training (SMLT = 0) and land resources (lfsize = 0), there is no statistically significant difference in WTP between male and female farmers. However, gender may play a more substantial role when interacting with other variables such as training and land access, as explored in the next section.

Interaction effects

This section examines how combinations of SLM training, gender, and farm size interact to influence farmer 'willingness to pay (WTP) for SLM practices. The inclusion of interaction terms helps identify conditional effects that are not captured by the main effects alone. The interaction between SLM training and gender *(SLMT x Male)* is positive and marginally significant (β = 0.0.315, $p < 0.1$), indicating that male farmers benefit more from SLM training than their female counterparts. This may reflect underlying gender disparities in access to productive assets, information, and decision-making authority [11, 12].

The interaction between SLM training and farm size *(SLMT x lfsize)* is positive and statistically significant ($\beta = 0.414$, $p < 0.05$), suggesting that the effect of training on willingness to pay is more pronounced among farmers with larger landholdings. This finding reinforces Hypothesis 2 (H2) and supports the economies of scale argument [27, 28], where larger farms

reduce per-unit investment costs. It also aligns with previous studies that identify access to resources and technical knowledge are key drivers of sustainable land management adoption [11, 14]. In contrast, the interaction between farm size and gender *(lfsize x male)* is negative and statistically insignificant ($\beta = -0.167$, $p < 0.1$), implying that gender does not significantly moderate the effect of farm size on WTP.

Notably, the three-way interaction between SLM training, gender, and farm size (SLM training × gender × farm size) is positive and statistically significant ($\beta = 0.375$, $p < 0.05$), confirming Hypothesis 3 (H3). This indicates that the effect of SLM training on willingness to pay (WTP) is jointly moderated by both gender and farm size. Specifically, the highest WTP is observed among male farmers with larger farms who have received SLM training. This finding underscores the relevance of adopting an intersectional approach when analysing agricultural decision-making and sustainability behaviours, as the interplay of socio-economic factors shapes farmers' adoption capacity and preferences.

Total marginal effects

This section presents the total marginal effects of SLM training, gender and farm size on willingness to pay (WTP) for Sustainable Land Management (SLM) practices, derived by combining both main effects and interaction effects. This comprehensive approach provides a clearer understanding of how these variables jointly influence WTP in real-world farming contexts. Table 5 summarises the total marginal effects across different combinations of gender, farm size, and training status. The results indicate that SLM training significantly increases WTP for all farmer groups; however, its impact is notably greater among farmers with larger landholdings, particularly male farmers. Among female farmers, the marginal effect of training increases from 0.164 to 0.578 as farm size increases. For male farmers, the effect

increases more sharply–from 0.479 to 1.268–suggesting that SLM training is most effective when combined with greater access to land and likely enhanced decision-making power [15].

Table 5: Total marginal effects of key predictors on WTP for SLM practices

A: Total Marginal effects of SLM training on WTP for SLM practices by farm size and gender					
Farmer Type	B1	B4	B5	B7	**Total marginal effects**
**Male=0 (lfsize=1)**	0.164	0.414	0	0	0.578
**Male=1 (lfsize=1)**	0.164	0.414	0.315	0.375	1.268
**Male=0 (lfsize=0)**	0.164	0	0	0	0.164
**Male=1 (lfsize=0)**	0.164	0	0.315	0	0.479
B: Total Marginal effects of farm size on WTP for SLM practices by SLM training and gender					
Farmer Type	**B2**	**B4**	**B6**	**B7**	**Total marginal effects**
**Male=0 (SLMT=1)**	0.058	0.414	0	0	0.472
**Male=1 (SLMT=1)**	0.058	0.414	–0.167	0.375	0.680
**Male=0 (SLMT=0)**	0.058	0	0	0	0.058
**Male=1 (SLMT=0)**	0.058	0	–0.167	0	–0.109
**B: Total Marginal effects of gender on WTP for SLM practices by SLM training and farm size**					
Farmer Type	**B3**	**B5**	**B6**	**B7**	**Total marginal effects**
**SLMT=0 (lfsize=1)**	0.03	0	–0.167	0	–0.137
**SLMT=1 (lfsize=1)**	0.03	0.315	–0.167	0.375	0.553
**SLMT=0 (lfsize=0)**	0.03	0	0	0	0.03
**SLMT=1 (lfsize=0)**	0.03	0.315	0	0	0.345

NB: B1=main effect of SLM training; B2=main effect of farm size; B3=main effect of gender; B4= SLMT x lfsize; B5= SLMT x Male; 56= lfsize x Male; B7= SLMT x Male x lfsize

Farm size demonstrates a more substantial effect on willingness to pay (WTP) when combined with SLM training. Among farmers who received SLM training, the marginal effect of farm size on WTP is 0.680 for men and 0.472 for women, indicating that training enhances the value of landholding in motivating sustainable investment. However, among untrained male farmers, the total marginal effect of farm size is negative (-0.109), suggesting that landholding alone is insufficient to drive willingness to investment in sustainable practices without complementary training or behavioural incentives [9].

Although gender alone is not statistically significant, its conditional effect becomes evident when combined with SLM training and farm size. The total effect of being male rises from 0.030 (in the absence of both SLM training and farm size) to 0.553 (when both are present), confirming that gendered patterns of investment are highly context-dependent. These results support existing evidence that gender gaps in agricultural adoption are mediated by differential access to resources and training opportunities [11, 12].

To visually support these estimates, Figure 2 presents the predictive margins, illustrating how willingness to pay (WTP) for SLM practices varies significantly by both gender and farm size. Among female farmers who received SLM training, WTP increases consistently with farm size, indicating that the benefits of training are amplified by greater access to land resources. Conversely, trained male farmers exhibit a declining WTP trend as farm size increases, indicating diminishing marginal returns to training among those who

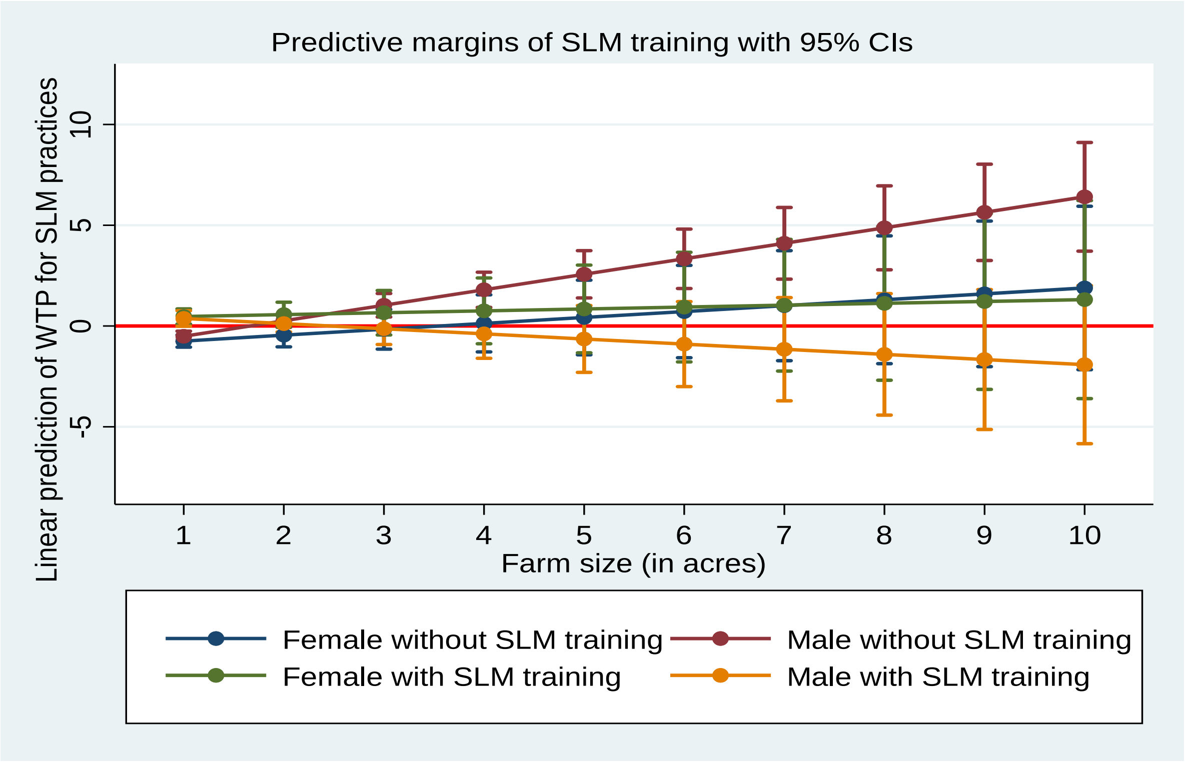

Fig 2: Predictive margins of SLM training by gender and farm size

already possess substantial holdings. This pattern may reflect a saturation effect, wherein extra training yields limited benefits for individuals with existing decision-making autonomy and productive capacity. Interestingly, untrained male farmers exhibit a rising WTP trend with increasing farm size, potentially due to unobserved exposure to informal knowledge networks or previous experience with sustainable practices not captured in the training variable. These dynamics are consistent with the findings of [15], who argue that the behavioural impacts of training interventions is most pronounced when foundational constraints, such as limited resources of knowledge gaps, are addressed.

Overall, while SLM training appears less effective—or potentially even counterproductive—among farmers with large landholdings and capital resources, its marginal effects are strongest among resource-constrained groups, especially women and smallholder farmers. This finding reinforces Hypothesis 3 (H3) and highlights the relevance of examining how interventions interact with socio-economic context, rather than relying solely on average treatment effects. Such an intersectional approach offers deeper insights into the conditions under which training interventions are most impactful.

Other covariates and WTP for SLM practices

Several covariates offer extra insights into the factors influencing farmers' willingness to pay (WTP) for Sustainable Land Management (SLM) practices. Income exerts a positive and statistically significant effect on WTP ($\beta = 0.046$, $p < 0.05$), indicating that as household income increases, farmers may become more risk-tolerant and financially capable of investing in environmental improvement. This finding is consistent with the "worth effect" argued in the Human Capital Theory [13] and is supported by research in environmental economics [18].

However, residing in the coastal zone has a negative and statistically significant association with WTP for SLM practice ($\beta = -0.169$, $p < 0.01$), suggesting that coastal farmers

are less willing to pay for SLM practices than their inland counterparts. These may be attributed to differences in livelihood strategies (i.e., greater reliance on fishing or trading), ecological conditions, or land tenure arrangements. In addition, because land degradation may be perceived as less severe in coastal areas, farmers may not prioritise land conservation.

Household dependency ratios also negatively affect WTP. The number of dependants in the households has a statistically significant negative effect ($\beta = -0.073$, $p < 0.05$), plausibly reflecting resource constraints or competing household needs. This is consistent with studies such as [10, 27], which show that households with more dependants often experience reduced capacity to invest in environmental public goods.

The effects of education on WTP are more complex. While primary education and other lower levels show no statistically significant effect, vocational education is negatively and statistically significantly associated with WTP ($\beta = -0.193$, $p < 0.05$). This may show the opportunity costs of environmental investment for individuals with alternative income-generating skills or less agricultural dependence. These findings resonate with [14], who argue that general education may not promote pro-environmental behaviours unless it explicitly incorporates environmental content.

Conclusions

This study investigated the gendered effects of Sustainable Land Management (SLM) training and farm size on farmers' willingness to pay (WTP) for SLM practices, using a Tobit regression analysis based on data from five municipalities in Ghana's Volta Region. The results show that both SLM training and farm size have positive and statistically significant effects on WTP, while gender moderates these effects in nuanced ways. Specifically, although male farmers benefit more from SLM training on average, female farmers—particularly those with larger landholdings—exhibit marginal increases in WTP as farm size increases. Additionally, several

contextual variables, including residence in coastal areas, higher household dependency ratios, lower income, and vocational education, are associated with WTP. These findings underscore the complex interplay between gender, access to productive resources, and knowledge acquisition in shaping environmental behaviour. They align with Human Capital Theory (HCT), which highlights the productivity-enhancing effects of training, and Behavioural Economics, which underscores the influence of social norms, financial constraints, and perceived risks on decision-making.

From a policy perspective, the findings highlight the significance of gender-responsive SLM programmes that promote equitable access to training, financing, and land ownership. Interventions such as microcredit schemes, inclusive extension services, and gender-based SLM training modules should prioritise female farmers with land access and male farmers with smaller landholdings, where the marginal benefits of training are greatest. The estimated WTP values also provide valuable inputs into participatory land valuation frameworks, as they reveal the perceived economic value of environmental services delivered through SLM practices.

The study has certain limitations. The cross-sectional design limits causal inference. While the results are robust across Tobit, Two-part, and Heckman models— indicating that the observed relationships are not model-specific—future research should consider instrumental variable Tobit models or panel data approaches to address unobserved heterogeneity and potential reverse causality. Such extensions would enhance the credibility and policy relevance of causal claims.

Acknowledgements

The authors are grateful to the editors and anonymous reviewers for their insightful comments and valuable time, which have significantly improved the quality of this paper.

References

1. Government of Ghana. Ministry of Local Government and Rural Development [Internet]. Ghana, 2016. Available from: http://www.ghanadistricts.com
2. Jinbaani AN. Sustainable land management in the Upper West region of Ghana: a forgotten solution to land degradation, communication failure or a non-priority option? [final project]. Reykjavik: United Nations University Land Restoration Training Programme; 2016 [cited 2022 Nov 4]. Available from: http://www.unulrt.is/static/fellows/document/jinbaani2016.pdf
3. Diao X, Sarpong DB. Poverty implications of agricultural land degradation in Ghana: an economy-wide, multimarket model assessment. African Development Review. 2011 Sep;23(3):263-75. https://doi.org/10.1111/j.1467-8268.2011.00285.x
4. McElwee P, Calvin K, Campbell D, Cherubini F, Grassi G, Korotkov V, Le Hoang A, Lwasa S, Nkem J, Nkonya E, Saigusa N. The impact of interventions in the global land and agri-food sectors on Nature's Contributions to People and the UN Sustainable Development Goals. Global Change Biology. 2020 Sep;26(9):4691-721. https://doi.org/10.1111/gcb.15219
5. Matlou R, Bahta YT, Owusu-Sekyere E, Jordaan H. Impact of agricultural drought resilience on the welfare of smallholder livestock farming households in the Northern Cape Province of South Africa. Land. 2021 May 27;10(6):562. https://doi.org/10.3390/land10060562
6. Ali EB, Agyekum EB, Adadi P. Agriculture for sustainable development: A SWOT-AHP assessment of Ghana's planting for food and jobs initiative. Sustainability. 2021 Jan 11;13(2):628. https://doi.org/10.3390/su13020628
7. Takele MT, Umer MI. Determinant Factors of Farmers' Willingness to Pay for Sustainable Land Management Practices: Evidence with Homosha Benishangul Gumuz Region:

Application with Multinomial Logit Model, Mixed Logit Model, and Conditional Logit Model. https://doi.org/10.21203/rs.3.rs-56133/v1

8. Alemu GT, Tsunekawa A, Haregeweyn N, Nigussie Z, Tsubo M, Elias A, Ayalew Z, Berihun D, Adgo E, Meshesha DT, Molla D. Smallholder farmers' willingness to pay for sustainable land management practices in the Upper Blue Nile basin, Ethiopia. Environment, Development and Sustainability. 2021 Apr;23(4):5640-65. https://doi.org/10.1007/s10668-020-00835-6
9. Amoah A, Jumah A, Korle K. Exploring willingness-to-pay for the restoration and maintenance of reserved forests in the Greater Accra Region of Ghana. Forest Ecosystems. 2022 Jan 1; 9:100041. https://doi.org/10.1016/j.fecs.2022.100041
10. Ndetewio PI, Mwakaje AG, Mujwahuzi M, Ngana J. Factors influencing willingness to pay for watershed services in lower Moshi, Pangani Basin, Tanzania. http://hdl.handle.net/123456789/1004
11. Quisumbing AR, Meinzen-Dick R, Raney TL, Croppenstedt A, Behrman JA, Peterman A. Closing the knowledge gap on gender in agriculture. In Gender in agriculture: Closing the knowledge gap 2014 Feb 19 (pp. 3-27). Dordrecht: Springer Netherlands. https://doi.org/10.1007/978-94-017-8616-4_1
12. Lee CJ. Gender-sensitivity in land management: trajectory of housing, agriculture and land ownership in South Korea. In Land governance and gender: The tenure-gender nexus in land management and land policy 2021 Dec 6 (pp. 91-99). Wallingford, UK: CABI. https://doi.org/10.1079/9781789247664.0008
13. Becker GS. Human capita. New York: National Bureau of Economic Research. 1964.
14. Burton RJ. The influence of farmer demographic characteristics on environmental behaviour: A review. Journal of Environmental Management. 2014 Mar 15; 135:19-26. https://doi.org/10.1016/j.jenvman.2013.12.005

15. Maharjan KL, Gonzalvo CM. Examining Farmers' Willingness to Learn Environmental Conservation Agriculture: Implications for Women Farmer Empowerment in Bagmati Province, Nepal. Agriculture. 2025 Mar 28;15(7):726. https://doi.org/10.3390/agriculture15070726
16. Kragt ME, Sarmiento JM, Rola-Rubzen MF. Key behavioural science concepts applied to farmer decision-making and farm risk management. https://doi.org/10.22004/ag.econ.369057
17. Byerly H, Kross SM, Niles MT, Fisher B. Applications of behavioural science to biodiversity management in agricultural landscapes: Conceptual mapping and a California case study. Environmental Monitoring and Assessment. 2021 Apr;193(Suppl 1):270. https://doi.org/10.1007/s10661-020-08815-z
18. Amoah A, Moffatt PG. Willingness to pay for reliable piped water services: evidence from urban Ghana. Environmental Economics and Policy Studies. 2021 Oct;23(4):805-29. https://doi.org/10.1007/s10018-021-00303-z
19. Akaateba MA. The politics of customary land rights transformation in peri-urban Ghana: Powers of exclusion in the era of land commodification. Land Use Policy. 2019 Nov 1; 88:104197. https://doi.org/10.1016/j.landusepol.2019.104197
20. Bonye SZ, Yiridomoh GY, Bebelleh FD. Compliance with land use regulations in peri-urban areas in Ghana: a study of Bamahu and Danko residential areas in Wa municipality, Upper West Region. GeoJournal. 2021 Dec;86(6):2845-59. https://doi.org/10.1007/s10708-020-10229-w
21. Ayano MF. Understanding the Local Complexities in Land Law Reforms: The Case of Land Inalienability in Ethiopia, 1991–2018. Law & Social Inquiry. 2024 Nov;49(4):2398-438. https://doi.org/10.1017/lsi.2024.28

22. Asamoah M, Owusu-Ansah F. Report on land tenure and cocoa production in Ghana. Cocoa Research Institute of Ghana and World Cocoa Foundation: Accra, Ghana. 2017 Feb.

23. Lambongang M, Ansah IG, Donkoh SA. Participation and yield effect of Ghana's planting for food and jobs programme in Bunkpurugu-Yunyoo district. Ghana Journal of Agricultural Economics and Agribusiness Vol. 2019 Jun;2(1).

24. Kidido JK, Bugri JT. Youth access to agricultural land from a peri-urban and rural perspective: a study of Techiman traditional area in Ghana. Journal of Land and Rural Studies. 2020 Jan;8(1):37-56. https://doi.org/10.1177/2321024919883145

25. Legesse W, Haji J, Ketema M, Emana B. Determinants of adoption of sustainable land management practice choices among smallholder farmers in Abay Basin of Oromia, Ethiopia. Journal of Development and Agricultural Economics. 2021 Mar;13(1):1-9. https://doi.org/10.5897/JDAE2020.1183

26. Abdeta D. Households' willingness to pay for forest conservation in Ethiopia: A review. Journal of Forest Science. 2022;68(11):437. https://doi.org/10.17221/94/2022-JFS

27. Fakayode SB, Ogunlade I, Ayinde O, Olabode P. Factors affecting farmers' ability to pay for irrigation facilities in Nigeria: the case of Oshin Irrigation Scheme in Kwara State. Journal of Sustainable Development in Africa. 2010 Dec 1;12(1):334-50.

28. Shepler R, Suter JF, Manning DT, Goemans C. Private Actions and Preferences for Coordinated Groundwater Conservation in Colorado's Republican River Basin. JAWRA Journal of the American Water Resources Association. 2019 Jun;55(3):657-69. https://doi.org/10.1111/1752-1688.12741

29. Tang Z, Nan Z, Liu J. The willingness to pay for irrigation water: A case study in Northwest China. Global Nest Journal. 2013 Mar 1;15(1):76-84.

30. Furlong M. ‘Clear at a distance, jumbled up close’: observation, immersion and reflection in the process that is creative research.

31. Krejcie RV, Morgan DW. Sample size determination table. Educational and Psychological Measurement. 1970;30(3):607-10.

32. Cochran WG. Sampling techniques. Johan Wiley & Sons Inc. 1977.

33. Tobin J. Estimation of relationships for limited dependent variables. Econometrica: Journal of the Econometric Society. 1958 Jan 1:24-36. https://doi.org/10.2307/1907382

34. Loomis JB. Balancing public trust resources of Mono Lake and Los Angeles' water rights: An economic approach. Water Resources Research. 1987 Aug;23(8):1449-56. https://doi.org/10.1029/WR023i008p01449

35. Cameron AC, Trivedi PK. Microeconometrics using Stata, Volume I: Cross-sectional and panel regression models. Stata Press; 2022.

36. Long JS, Freese J. Predicted probabilities for count models. The Stata Journal. 2001 Nov;1(1):51-7. https://doi.org/10.1177/1536867X0100100103

37. Kramer M, Weldon PJ. Constructing composite scores for contemporaneous behaviours: A comparison of four approaches. Ethology. 2023 Sep;129(9):489-97. https://doi.org/10.1111/eth.13382

38. Fiagborlo JD. Essays on transport mode choice, fuel and ICT expenditures in Ghana (Doctoral dissertation, University of Cape Coast).

39. Hair JF, Black WC, Babin BJ, Anderson RE, Black WC, Anderson RE. Multivariate data analysis (Eighth Edi). Hampshire, United Kingdom: Cengage Learning EMEA. https://doi. org/10.1002/9781119409137. ch4. 2019.

40. Watkins MW. Exploratory factor analysis: A guide to best practice. Journal of black psychology. 2018 Apr;44(3):219-46. https://doi.org/10.1177/0095798418771807

41. Asante BK. The economy is not healthy: whose fault? Daily Graphic. 2013;(19214):7.

42. Whittington D. Ethical issues with contingent valuation surveys in developing countries: A note on informed consent and other concerns. Environmental and Resource Economics. 2004 Aug;28(4):507-15. https://doi.org/10.1023/B:EARE.0000036776.89379.4f

43. Hansen BB, Pedersen ÅØ, Peeters B, Le Moullec M, Albon SD, Herfindal I, Sæther BE, Grøtan V, Aanes R. Spatial heterogeneity in climate change effects decouples the long-term dynamics of wild reindeer populations in the high Arctic. Global Change Biology. 2019 Nov;25(11):3656-68. https://doi.org/10.1111/gcb.14761

44. Malone HE, Coyne I. Complementing the P-value from null-hypothesis significance testing with a Bayes factor from null-hypothesis Bayesian testing. Nurse Researcher. 2020 Dec 16;28(4). https://doi.org/10.7748/nr.2020.e1756

45. Sribney W. Chi-squared test for models estimated with robust standard errors. StataCorp, August. http://www. stata. com/support/faqs/stat/chi2. html. 1997.

Appendix

Table 6: Heckman selection model as a robustness check for baseline Tobit model

Variables	Tobit model	Heckman eqn. 1	Heckman eqn. 2
Training (SLMT)	0.164***		0.113***
	(0.029)		(0.0325)
Log of farm size (lfsize)	0.058***	0.0858***	–0.176
	(0.020)	(0.0213)	(0.111)
Gender (Male)	0.030	–0.0321	0.0917*
	(0.027)	(0.0314)	(0.0485)
SLMT x lfsize	0.414**	-	0.548***
	(0.142)	-	(0.029)
SLMT x Male	0.315*	-	0.512***
	(0.174)	-	(0.035)
Lfsize x Male	–0.167	0.329***	0.459***
	(0.110)	(0.020)	(0.021)
SLMT x Male x lfsize	0.375**	-	0.522***
	(0.196)	-	(0.039)
Coastal	–0.169***	–0.142***	0.184
	(0.028)	(0.0292)	(0.177)
Log of income	0.046**	0.0372*	–0.0652
	(0.019)	(0.0225)	(0.0508)
Log of experience	–0.001	0.0123	-
	(0.016)	(0.0180)	-
Household dependant	–0.066**	0.00865	–0.108***
	(0.026)	(0.0273)	(0.0295)
Primary education	0.043	0.0347	–0.0884
	(0.049)	(0.0569)	(0.0688)
Middle sch. education	0.049	0.0409	–0.0694
	(0.043)	(0.0511)	(0.0685)
Secondary education	–0.008	0.151**	–0.498***
	(0.064)	(0.0646)	(0.185)
Vocational education	–0.193**	0.110	–0.520***
	(0.083)	(0.0783)	(0.154)
Tertiary education	–0.050	0.190**	–0.536**
	(0.076)	(0.0895)	(0.227)
Inverse mills ratio (IMR)			–1.411**
Observations	1,036	1,036	1,036

Standard errors in parentheses *** p<0.01, ** p<0.05, * p<0.1

8th January, 2026

Dear Editor-in-Chief

Subject: Revision and Resubmission of Manuscript Number: PONE-D-25-40765R1

We are grateful for the opportunity to address several minor issues related to clarity, language, and presentation, and resubmit our manuscript titled "***Gendered dimensions of sustainable land management: evidence of farm size and training effects on willingness to pay in Ghana's Volta Region***" for consideration in PLOS ONE.

The responses to each comment in the revised manuscript are summarised in the table below, providing a point-by-point explanation of how the concerns were resolved. All co-authors have reviewed and approved the revised manuscript, and I remain the corresponding author for this submission.

Thank you once again for your time and consideration.

Sincerely,

James Dickson Fiagborlo (Ph.D.),
Faculty of Applied Social Sciences,
Department of Multidisciplinary Studies,
Ho Technical University,
Ho, Ghana.

Corresponding Author

SN	1st Reviewer's comment	Author's response to reviewer 1	SN	2nd Reviewer's comment	Author's response to reviewer 2	SN	3rd Reviewer's comment	Author's response to reviewer 3
1.	The title is not supported by the background literature. Nevertheless, and according to your literature findings, you mentioned two issues (farm size and SLM training and their effects on WTP) related to your hypotheses, the gender was not mentioned. The title must reflect the main hypothesis (the research main question).	Thank you for your thoughtful comment regarding the alignment between the manuscript's title and its background literature. In response, we revised the background section of the manuscript to explicitly incorporate gender as a core analytical dimension. The revised section now clearly articulates the role of gender in shaping farmers' willingness to pay for Sustainable Land Management practices, alongside farm size and training–thereby providing stronger justification for the inclusion of gender in the study's hypotheses and title.<br><br>In particular, we have added relevant literature that discusses gender differences in agricultural decision-making, access to productive resources,	1	The manuscript (i.e., the written document or paper) has fully and thoroughly responded to or incorporated the corrections or feedback that were previously given.<br>However, including the DOIs of the cited articles would enhance the rigor of the manuscript and make it more appealing to readers. Therefore, I kindly ask that the DOI for each referenced article be included in the manuscript.	Thank you for your positive feedback and for acknowledging the revisions made to address the earlier comments. In response to your suggestions, the revised manuscript now include DOIs for the majority of the cited papers in the reference list. Where a DOI was not available, full citation details have been provided to maintain traceability. For details, please refer to the reference list. We hope this addresses your concern. Thanks	1	I have no further suggestions, The author should further emphasize the significance of the research and highlight the core value of this study.	Thanks for your thoughtful feedback. The revised manuscript already emphasised the significance and core contribution of the study in the introduction section. In particular, the study highlights three core areas: (1) It provides empirical evidence on how gender dynamics, training, and farm size interact to influence sustainability-oriented behaviour among farming households in Ghana; (2) It applies a gender-sensitive analytical framework to assess willingness to pay for Sustainable Land Management practices, contributing to the broader literature on environmental and development economics; (3) It offers policy-relevant insights to inform gender-equitable

		and adoption of sustainable practices. These additions provide a clearer conceptual foundation for the gender-related hypothesis and reinforce the relevance of gender in the analysis.<br><br>Please refer to page 4, lines 76-86 for the detailed revisions<br><br>We hope this revision addresses your concern. Thank you again for valuable feedback.						agricultural extension, land management, and climate adaptation strategies, supporting more inclusive and sustainable rural development. For more details, please refer to page 5, lines 112 -118.<br><br>I hope this satisfactorily addresses your concerns.
2.	You result in the abstract are not matching with what you have investigated. Focus the abstract results on findings that directly align with the stated hypothesis and objectives, with the impact of gender differences presented as the primary result. The conclusion is not related very much to the study results or to the hypotheses..	Thank you very much for this insightful comment. In response, we have revised the abstract to explicitly link the study's key findings to its stated objectives and hypotheses. The clarification strengthens the internal coherence of the paper<br><br>The revisions can be found on page 2, lines 34–46.<br>We hope that our response addresses	2					

		your concerns satisfactorily.						
3.	The introduction should concisely review both theoretical and empirical literature on gender relevant to the research questions. Strengthen the literature review with additional gender studies from diverse African or international contexts. The hypothesis should be grounded in strong evidence from this literature review. The section needs a language revision.	We are grateful for this insightful comment. In response, we have revised the manuscript to incorporate relevant theoretical and empirical literature on gender as it pertains to the research. Specifically, we strengthened the literature review by including additional global studies that highlight gender and sustainable land management.<br><br>Furthermore, in the revised manuscript, the hypotheses are now directly derived from the identified gaps following the literature review. The entire manuscript has been edited accordingly.<br><br>Please refer to pages 4–10, lines 76–97 for the theoretical discussion, and lines 219–225 for the hypotheses. We hope these revisions	3					

		satisfactorily addresses your concerns. We appreciate your valuable feedback						
4.	Review the methodology to determine if it is appropriate for the study population, considering cluster or stratified methods. Remove the extraneous information about measured variables or otherwise move them to the relevant section. Focus the study model description on the specific methods used, removing well-known equations. Reorganize the section for clarity and use scientific language.	Thank you for your kind comments and helpful suggestions. In response, we have revised the entire methodology section to address the concerns raised.<br><br>In particular, the revised section now clearly outlines the study design and sampling procedures. We have also refined the description of the models, focusing on the specific methods used in the study and removing standard or widely known equations to enhance clarity.<br><br>Additionally, we have edited the entire methodology section for consistency in scientific language and overall clarity.<br><br>Please refer to pages 10–14, lines 228–239 for details on the study design and	4				.	

		sampling procedure, and lines 260–314 for the information on the measured variables and model specification.<br><br>We hope this revisions satisfactorily address your concerns and improve the quality of the manuscript.						
5.	Clearly present the results, addressing each research question. Name the tables and refer to them in the appropriate position. Avoid explaining methodology; use direct, simple scientific language to clarify findings.	We are grateful for your insightful feedback. In response to your comments, we have revised the entire Results and Discussion section of the manuscript. Specifically, the empirical findings are now structured to distinguish between the main effects and conditional effects of the predictor variables. We have improved the scientific clarity of the language used to ensure that each finding is explicitly linked to the study's hypotheses.<br><br>While we were unable to adopt the reference style you suggested,	5					

		we have maintained consistency and clarity in citations throughout the section.<br><br>Please refer to pages 18–28, lines 392–553 for the detailed revisions. We hope these revisions meet your expectations and satisfactorily address your concerns.						
6.	Acknowledgments usually thank professional, academic, and personal support, progressing from formal (supervisor, funding bodies) to informal (peers, family, friends). Accordingly, directly acknowledge the persons who help you.	We sincerely thank you for your helpful suggestions and fully agree with your insightful observations. In response, we have revised the acknowledgements section to reflect your feedback.<br><br>Please refer to page 28, lines 584–586 for the updated text. We hope this revision satisfactorily addresses your concerns.						

RESPONSE TO EDITOR'S REQUEST

Thank you for your valuable comment regarding repetition in the manuscript. In response, we have revised the sections on Main effects, Interaction effects, and Total marginal effects to reduce redundancy while maintaining analytical clarity. Overlapping explanations and repetitive interpretations have been streamlined, and transitions between sections have been improved to ensure coherence and logical flow.

The revised version now presents each analytical component distinctly and succinctly, in line with the Journal's editorial standards and to enhance overall readability.

For further details, please refer to pages 21-26, lines 441-527 of the revised manuscript.
