# Supplementary material for: Gendered dimensions of sustainable land management: Evidences from farm size and training effects on willingness to pay in Ghana’s Volta Region
Source: PLoS One. 2026 Jun 11;21(6):e0351424. doi: 10.1371/journal.pone.0351424 (PMC13257986; doi:10.1371/journal.pone.0351424)
Supplement: Attachment — Submitted filename: Gendered dimensions report 1.docx [file pone.0351424.s005.docx]

Gendered dimensions of sustainable land management: evidence of farm size and training effects on willingness to pay in Ghana’s Volta Region

PONE-D-25-40765

The reviewer report

**Abstract**

Provide a concise background statement to contextualize the study and highlight its significance. Expand the methodology section with additional details. Focus the abstract results on findings that directly align with the stated hypothesis and objectives, with the impact of gender differences presented as the primary result. Emphasize the study's main results in relation to the problem statement.

**Introduction**

In a single section, the introduction should concisely review both theoretical and empirical literature on gender relevant to the research questions. Bolster the literature review with additional gender studies from diverse African or international contexts. The hypothesis should be grounded in strong evidence from this literature review. The section needs an editing and language revision.

**Methodology**

Review the methodology to determine if it is appropriate for the study population, considering cluster or stratified methods. Move extraneous information about measured variables to the introduction or other relevant sections. Focus the study model description on the specific methods used, removing well-known equations. Reorganize the section for clarity and use scientific language.

**Results**

Clearly present the results, addressing each research question. Reference relevant tables and figures at the end of the statement or paragraph. Avoid explaining methodology; use direct, simple scientific language to clarify findings.

**Acknowledgement**

Acknowledgments usually thank professional, academic, and personal support, progressing from formal (supervisor, funding bodies) to informal (peers, family, friends).

Accordingly directly acknowledge the persons who help you.
