# Supplementary material for: Gendered dimensions of sustainable land management: Evidences from farm size and training effects on willingness to pay in Ghana’s Volta Region
Source: PLoS One. 2026 Jun 11;21(6):e0351424. doi: 10.1371/journal.pone.0351424 (PMC13257986; doi:10.1371/journal.pone.0351424)
Supplement: Attachment — Submitted filename: PONE-D-25-40765-reviewer comments and suggestions.docx [file pone.0351424.s006.docx]

**Reviewer Comments and Suggestions**

**Review Report**

General observation
This manuscript presents a timely and policy-relevant analysis of the factors influencing the **Gendered dimensions of sustainable land management: evidence of farm size and training effects on willingness to pay in Ghana’s Volta Region.** Its strengths lie in the robust methodological approach, particularly the use of the Tobit model, which effectively captures the interdependence among conservation practices—a key advancement over more simplistic models. The data is well-presented, and the policy implications are grounded in empirical evidence. However, the manuscript would benefit from improved clarity in variable testing for multicollinearity, deeper engagement with recent comparative literature from other sub-Saharan African contexts, and a more thematically structured discussion section.

The study addresses a significant topic with clear policy implications and contributes to the discourse on sustainable land management. This research study involves the Tobit model assessing the factors associated with the willingness to pay to SLM. Further, the data are well-presented, and the policy implications are grounded in empirical evidence. Nonetheless, the manuscript would benefit from greater clarity and rigor in the following areas:

- Selection and justification of variables
- Analytical strategy
- Engagement with recent comparative literature, especially from other continents
- A thematically organized discussion section

**Minor Revisions Required**

1. Clarify the Focus on only one dependent variable (willingness to pay)
   - Why does the study focus specifically on MIPs? What are the implications of this focus?
   - Clearly define the variables and explain the relevance of selecting them as focal areas.
2. Clarification of Variables
   - A clear and detailed explanation of the variables used is essential.
   - Consider including a table or appendix that defines and explains all key variables.

**Title**

Assessment & Suggestion

- Assessment: The title is comprehensive and accurately reflects the study’s focus on the variables influencing foreign agricultural land holdings, particularly those that lie outside agricultural market interests and intersect with military interest points.
- Suggestion: Consider adding the phrase *“using a Tobit model”* or *“an econometric approach”* in the subtitle to highlight the methodological focus.

Suggested titles

1. **Exploring Gendered Dimensions of Sustainable Land Management: The Impact of Farm Size and Training on Willingness to Pay in Ghana’s Volta Region**
2. **Gender, Farm Size, and Training: Determinants of Willingness to Pay for Sustainable Land Management in Ghana’s Volta Region**

**Abstract**

- The abstract is well-structured, clearly outlining the study’s background, objectives, key findings, and policy implications.
- Suggestions:
  - Include the sample size of the study
  - Include the econometric model(s) used.
  - State the main significant predictors and their effects.
  - Include key figures or statistics to enhance clarity.

**Introduction**

- The introduction successfully contextualizes the issue of sustainable land management and outlines the policy background.
- However, it would benefit from a more detailed theoretical foundation to clearly identify the research gap

Suggestions:

- Develop a conceptual framework informed by relevant literature.
- Include a recent comparative study from another continent or region to situate the analysis globally.

**Methodology**

In this study, I found an excellent data collection technique. However, it is critical to consider the suggestions mentioned below.

- Clarify whether the variables were statistically tested and whether the model passed standard diagnostic checks (e.g., goodness-of-fit).

Assumptions of the Tobit Model

1. Censoring mechanism is known and correctly specified: Data is censored from above, below, or both, and this is properly defined.
2. Latent variable is linearly related to predictors: The underlying latent variable (unobserved variable) follows a linear model.
3. Normality of errors: The error term follows a normal distribution (Gaussian).
4. Homoscedasticity: Constant variance of error terms across values of independent variables.
5. Independence: Observations are independent of one another.
6. No perfect multicollinearity: Predictors are not perfectly correlated.

I suggest including evidence of a multicollinearity test for your variables and further analytic explanations regarding the employed model. Additionally, you should verify the assumptions and analytical framework for this model.

**Results and Discussion**

- The discussion section would be stronger with more references to comparative and recent literature.
- Organize the section using clear thematic sub-headings (e.g., Gender Differences in SLM Training and Farm Size, SLM Training and Farm Size Impact on WTP, Gender-Specific Effects on Willingness to Pay)

**Conclusion**

- More clearly tie the empirical findings to the broader context of gender perspectives on sustainable land management and the variable you considered.
- Reiterate the importance and relevance of your variables.
- Suggest actionable policy recommendations or scaling strategies for land value assessment frameworks.
