# Supplementary material for: Gendered dimensions of sustainable land management: Evidences from farm size and training effects on willingness to pay in Ghana’s Volta Region
Source: PLoS One. 2026 Jun 11;21(6):e0351424. doi: 10.1371/journal.pone.0351424 (PMC13257986; doi:10.1371/journal.pone.0351424)
Supplement: Attachment — Submitted filename: Comments.docx [file pone.0351424.s007.docx]

**Comments**

this paper addresses a topic of clear academic and policy relevance. The manuscript demonstrates the author’s solid effort in data collection and preliminary modeling. However, several important issues need to be addressed before the paper can reach publishable quality. The comments below aim to provide constructive feedback to help strengthen the theoretical framing, empirical rigor, and interpretative coherence of the study.

**1. Issues with model specification and interpretation.**

There are significant problems in the specification and interpretation of the regression model. The author includes both the main effects and the interaction terms in the same model, yet interprets the insignificance of a main effect as evidence of “no impact” on the dependent variable. This interpretation is econometrically invalid. When an interaction term is included, the coefficient of the main effect represents only the marginal effect when the moderator takes the value of zero (or its reference category). The total effect is the combination of the main and interaction effects. Consequently, a previously significant main effect may become statistically insignificant once its interaction with the moderator is introduced—without implying the absence of influence. The current interpretation therefore risks misrepresenting variable importance and overlooking the mechanisms implied by the interaction term, suggesting that the model specification itself is conceptually flawed.

**2. Weak linkage between the central argument and empirical analysis.**

There is a clear disconnection between the paper’s stated research focus and the empirical implementation. Although the title highlights a *gender dimension*, this theme is neither theoretically developed nor consistently integrated throughout the manuscript. The theoretical framework does not adequately explain why or how gender functions as a key analytical dimension. In the empirical section, gender appears only as a control variable or as part of an interaction term, without substantive discussion of gender-specific mechanisms. Moreover, the conclusion fails to return to the gender perspective to extract policy or theoretical implications. This disjunction weakens the thematic coherence of the study and diminishes its originality and theoretical contribution.

**3. Inconsistencies between empirical findings and conclusions.**

There appear to be discrepancies between the reported empirical results and the conclusions drawn from them. These inconsistencies should be carefully checked and clarified to ensure internal coherence and the credibility of the findings.

**4. Insufficient empirical depth and robustness.**

The empirical analysis lacks sufficient depth and rigor. The current presentation of baseline regressions represents only an initial step. To establish the robustness of the findings, the author should conduct additional robustness checks—such as alternative variable measurements, modified model specifications, or sub-sample analyses. Furthermore, heterogeneity analyses could yield richer insights by examining whether the key relationships differ across groups (beyond gender, e.g., by region, age, or other socio-economic characteristics). Most importantly, potential endogeneity issues—such as omitted variables or reverse causality—must be acknowledged and addressed, for instance through instrumental variable approaches. Without these standard robustness and validity tests, the credibility and scholarly value of the results remain limited.
