# Supplementary material for: Gendered dimensions of sustainable land management: Evidences from farm size and training effects on willingness to pay in Ghana’s Volta Region
Source: PLoS One. 2026 Jun 11;21(6):e0351424. doi: 10.1371/journal.pone.0351424 (PMC13257986; doi:10.1371/journal.pone.0351424)
Supplement: Attachment — Submitted filename: RESPONSE TO REVIEWERS COMMENTS.docx [file pone.0351424.s008.docx]

21^st^ November, 2025

Dear Editor-in-Chief

**Subject: Revision and Resubmission of Manuscript Number:  PONE-D-25-40765**

We appreciate the opportunity to revise and resubmit our manuscript titled "***Gendered dimensions of sustainable land management: evidence of farm size and training effects on willingness to pay in Ghana’s Volta Region***" for consideration in PLOS ONE.

We are grateful for the reviewers’ constructive comments, which have significantly improved the clarity and rigour of our paper, particularly with respect with background and theoretical issues, literature review, methodological issues, and other key components.

In response, we have carefully addressed each comment and summarised our revision in the table below, providing a point-by-point explanation of how the concerns were resolved. All co-authors have reviewed and approved the revised manuscript, and I remain the corresponding author for this submission.

Thanks you once again for your time and consideration.

Sincerely,

James Dickson Fiagborlo (Ph.D.),

Faculty of Applied Social Sciences,

Department of Multidisciplinary Studies,

Ho Technical University,

Ho, Ghana.

**Corresponding Author**

| **SN** | **1^st^ Reviewer’s comment** | **Author’s response to reviewer 1** | SN | **2^nd^ Reviewer’s comment** | **Author’s response to reviewer 2** | **SN** | **3^rd^ Reviewer’s comment** | **Author’s response to reviewer 3** |
| --- | --- | --- | --- | --- | --- | --- | --- | --- |
| 1. | Provide a concise background statement to contextualize the study and highlight its significance. Expand the methodology section with additional details. Focus the abstract results on findings that directly align with the stated hypothesis and objectives, with the impact of gender differences presented as the primary result. Emphasize the study's main results in relation to the problem statement. | Thank you very much for this insightful comment. In response, we have added a background statement to better contextualise the study and emphasise its relevance. Additionally, the revised abstract now includes key methodological details, with findings clearly linked to the study’s objectives and hypotheses. These revisions can be found on page 1, lines 26–46. We are grateful for your thoughtful feedback, which prompted these important clarifications. We hope our response addresses your concerns satisfactorily. | 1 | General observation This manuscript presents a timely and policy-relevant analysis of the factors influencing the **Gendered dimensions of sustainable land management: evidence of farm size and training effects on willingness to pay in Ghana’s Volta Region.** Its strengths lie in the robust methodological approach, particularly the use of the Tobit model, which effectively captures the interdependence among conservation practices—a key advancement over more simplistic models. The data is well-presented, and the policy implications are grounded in empirical evidence. However, the manuscript would benefit from improved clarity in variable testing for multicollinearity, deeper engagement with recent comparative literature from other sub-Saharan African contexts, and a more thematically structured discussion section.  The study addresses a significant topic with clear policy implications and contributes to the discourse on sustainable land management. This research study involves the Tobit model assessing the factors associated with the willingness to pay to SLM. Further, the data are well-presented, and the policy implications are grounded in empirical evidence. Nonetheless, the manuscript would benefit from greater clarity and rigor in the following areas:   - Selection and justification of variables - Analytical strategy - Engagement with recent comparative literature, especially from other continents - A thematically organized discussion section | Thank you very much for this insightful comment. In response, we have conducted several diagnostic analyses to test for multicollinearity among the independent variables. Additionally, we have engaged with recent comparative literature from other sub-Saharan African contexts and restructured both the literature review and results sections thematically to improve clarity. Please refer to pages 3–9, lines 76–98 for the revised theoretical issues, and pages 14–28, lines 316–586 for the detailed diagnostic test and results analysis. We hope these revisions satisfactorily address your concerns. | 1 | This paper addresses a topic of clear academic and policy relevance. The manuscript demonstrates the author’s solid effort in data collection and preliminary modeling. However, several important issues need to be addressed before the paper can reach publishable quality. The comments below aim to provide constructive feedback to help strengthen the theoretical framing, empirical rigor, and interpretative coherence of the study. | We are grateful to you for your constructive feedback, which has helped strengthen the theoretical framing, empirical rigor, and interpretative coherence of the study. We sincerely appreciate your support. |
| 2. | In a single section, the introduction should concisely review both theoretical and empirical literature on gender relevant to the research questions. Bolster the literature review with additional gender studies from diverse African or international contexts. The hypothesis should be grounded in strong evidence from this literature review. The section needs an editing and language revision. | We are grateful for this insightful comment. In response, we have revised the entire introduction to incorporate relevant theoretical and empirical literature on gender as it pertains to the research. In addition, we strengthened the literature review by including more global studies on gender and sustainable land management. In the revised manuscript, the hypotheses are now directly derived from the identified gaps following the literature review. The entire manuscript has been edited accordingly.  Please see pages 3–9, lines 76–97 for the theoretical issues, and lines 219–225 for hypotheses. We hope this revision satisfactorily addresses your concerns. | 2 | **Minor revisions required**   1. Clarify the Focus on only one dependent variable (willingness to pay)    - Why does the study focus specifically on MIPs? What are the implications of this focus?    - Clearly define the variables and explain the relevance of selecting them as focal areas. 2. Clarification of Variables    - A clear and detailed explanation of the variables used is essential.    - Consider including a table or appendix that defines and explains all key variables. | Thank you for your kind comments and helpful suggestions. In response, we have revised the entire methodology section to address the concerns raised. In particular, the revised section now clearly defines all key variables and explains their relevance to the study. Additionally, we have included a table that presents the definitions and descriptions of the variables for greater clarity.  Please refer to pages 11–13, lines 260–288 for details. We hope this revisions satisfactorily address your concerns. | 2 | There are significant problems in the specification and interpretation of the regression model. The author includes both the main effects and the interaction terms in the same model, yet interprets the insignificance of a main effect as evidence of “no impact” on the dependent variable. This interpretation is econometrically invalid. When an interaction term is included, the coefficient of the main effect represents only the marginal effect when the moderator takes the value of zero (or its reference category). The total effect is the combination of the main and interaction effects. Consequently, a previously significant main effect may become statistically insignificant once its interaction with the moderator is introduced—without implying the absence of influence. The current interpretation therefore risks misrepresenting variable importance and overlooking the mechanisms implied by the interaction term, suggesting that the model specification itself is conceptually flawed. | We are grateful for your insightful feedback and fully agree with your observations. In response to your comments, we have revised the entire Results and Discussions section of the manuscript to address the concerns raised. Specifically, the empirical findings are now structured around the main and conditional effects of the predictor variables, and these findings are explicitly linked to the hypotheses of the study.  Please refer to pages 18–28, lines 392–586 for the detailed revisions.  We hope these revisions meet your expectations and satisfactorily address your concerns. |
| 3. | Review the methodology to determine if it is appropriate for the study population, considering cluster or stratified methods. Move extraneous information about measured variables to the introduction or other relevant sections. Focus the study model description on the specific methods used, removing well-known equations. Reorganize the section for clarity and use scientific language | Thank you for your kind comments and helpful suggestions. In response, we have revised the entire methodology section to address the concerns raised. In particular, the revised section now clearly outlines the study design and sampling procedures. Additionally, we have refined model description to focus on the specific analytical methods used, removing standard or well-known equations. The entire methodology section has been edited for clarity and consistency in the use of scientific language. Please refer to pages 10–14, lines 228–239 for details on the study design and sampling procedure, and lines 260–314 for information on the measured variables and model specification. We hope this revisions satisfactorily address your concerns. | 3 | - Assessment: The title is comprehensive and accurately reflects the study’s focus on the variables influencing foreign agricultural land holdings, particularly those that lie outside agricultural market interests and intersect with military interest points. - Suggestion: Consider adding the phrase *“using a Tobit model”* or *“an econometric approach”* in the subtitle to highlight the methodological focus. | We are grateful for this insightful comment and helpful suggestion. In response, while we appreciate the value of your recommendation, we have chosen to retain the original title for the sake of concision and clarity. Moreover, the methodological aspects of the study are thoroughly addressed in the main body of the paper. We hope this response satisfactorily addresses your concerns. | 3 | There is a clear disconnection between the paper’s stated research focus and the empirical implementation. Although the title highlights a *gender dimension*, this theme is neither theoretically developed nor consistently integrated throughout the manuscript. The theoretical framework does not adequately explain why or how gender functions as a key analytical dimension. In the empirical section, gender appears only as a control variable or as part of an interaction term, without substantive discussion of gender-specific mechanisms. Moreover, the conclusion fails to return to the gender perspective to extract policy or theoretical implications. This disjunction weakens the thematic coherence of the study and diminishes its originality and theoretical contribution. | We sincerely thank you for your insightful comments and suggestions aimed at improving the quality of the manuscript. In response, we have revised the manuscript extensively to clarify how gender functions as a central analytical dimension through the study. Specifically, we have integrated relevant theoretical and empirical literature on gender into the background section to strengthen the conceptual framing. In the empirical analysis, gender is now treated not only as control variable or within interaction terms, but also as a substantive factor, with detailed discussion of its role and implications within the context of the study.  Please refer to pages 3–9, lines 76–97 for the revised theoretical discussion, and lines 392–615 for revision related to the empirical results and conclusions on gender.  We hope these revisions satisfactorily address your concerns. |
| 4. | Clearly present the results, addressing each research question. Reference relevant tables and figures at the end of the statement or paragraph. Avoid explaining methodology; use direct, simple scientific language to clarify findings. | We are grateful for your insightful feedback. In response to your comments, we have revised the entire Results and Discussions section of the manuscript. Specifically, the empirical findings are now structured around the main and conditional effects of the predictor variables. While we were unable to adopt the reference style you suggested, we have used clear scientific language to improve the presentation of the findings. Furthermore, the results have also been explicitly linked to the hypotheses of the study. Please refer to pages 18–28, lines 392–586 for the detailed revisions. We hope these revisions meet your expectations and satisfactorily address your concerns. | 4 | 1. Exploring Gendered Dimensions of Sustainable Land Management: The Impact of Farm Size and Training on Willingness to Pay in Ghana’s Volta Region 2. Gender, Farm Size, and Training: Determinants of Willingness to Pay for Sustainable Land Management in Ghana’s Volta Region | We are grateful for this insightful comment and helpful suggestion. In response, while we appreciate the value of your recommendation, we have chosen to retain the original title for the sake of concision and clarity. Moreover, the methodological aspects of the study are thoroughly addressed in the main body of the paper. We hope this response satisfactorily addresses your concerns. | 4 | There appear to be discrepancies between the reported empirical results and the conclusions drawn from them. These inconsistencies should be carefully checked and clarified to ensure internal coherence and the credibility of the findings. | We are grateful for this insightful comment and helpful suggestion. In response, we have addressed all the concerns raised regarding the inconsistencies in order to enhance internal coherence and strengthen the credibility of the findings.  Please refer to the empirical and conclusion sections on pages 392–615 for details.  We hope this response satisfactorily addresses your concerns. |
| 5. | Acknowledgments usually thank professional, academic, and personal support, progressing from formal (supervisor, funding bodies) to informal (peers, family, friends).  Accordingly directly acknowledge the persons who help you. | We thank you very much for your helpful suggestions. We fully agree with your insightful observations. In response, we have revised the acknowledgements section accordingly. Please refer to page 29, lines 617–619 for the updated text. We hope this revision satisfactorily addresses your concerns. | 5 | - The abstract is well-structured, clearly outlining the study’s background, objectives, key findings, and policy implications. - Suggestions:   - Include the sample size of the study   - Include the econometric model(s) used.   - State the main significant predictors and their effects.   - Include key figures or statistics to enhance clarity. | Thank you very much for your insightful observations and helpful suggestions. In response, we revised the abstract to include key methodological details, with the main significant predictors and their effects clearly linked to the study’s objectives and hypotheses. Relevant figures and statistics have been incorporated to enhance clarity.  These revisions can be found on page 1, lines 26–46. We are grateful for your thoughtful feedback, which prompted these important improvements. We hope our response satisfactorily addresses your concerns. | 5 | The empirical analysis lacks sufficient depth and rigor. The current presentation of baseline regressions represents only an initial step. To establish the robustness of the findings, the author should conduct additional robustness checks—such as alternative variable measurements, modified model specifications, or sub-sample analyses. Furthermore, heterogeneity analyses could yield richer insights by examining whether the key relationships differ across groups (beyond gender, e.g., by region, age, or other socio-economic characteristics). Most importantly, potential endogeneity issues—such as omitted variables or reverse causality—must be acknowledged and addressed, for instance through instrumental variable approaches. Without these standard robustness and validity tests, the credibility and scholarly value of the results remain limited | Thank you very much for your insightful observations and helpful suggestions. We fully agree with you that the presentation of these baseline regressions represents only an initial step in the analysis. In response to your comments, we have conducted several robustness and validity checks to support the reliability of our findings. In addition, we performed heterogeneity analyses to provide further insights into the baseline estimates. The direction and statistical significance of key predictor variables—particularly SLM training and farm size—remain consistent across alternative model specifications, thereby confirming the robustness of the baseline estimates. Furthermore, the sub-sample analyses reveal heterogeneity in effects across gender and farming experience.  For further details, please refer to pages 20–21, lines 392–438.  We hope this revision satisfactorily addresses your concerns. |
| 6. |  |  | 6 | - The introduction successfully contextualizes the issue of sustainable land management and outlines the policy background. - However, it would benefit from a more detailed theoretical foundation to clearly identify the research gap | We are grateful for this insightful comment. In response, we have revised the entire introduction to incorporate relevant theoretical and empirical literature on gender as it pertains to the research. In addition, we strengthened the literature review by including more global studies on gender and sustainable land management. In the revised manuscript, the hypotheses are now directly derived from the identified gaps following the literature review. The entire manuscript has been edited accordingly.  Please see pages 3–9, lines 76–98 for the theoretical issues, and lines 219–223 for hypotheses. We hope this revision satisfactorily addresses your concerns. | 6 |  |  |
| 7. |  |  | 7 | - Develop a conceptual framework informed by relevant literature. - Include a recent comparative study from another continent or region to situate the analysis globally. | We are grateful for this insightful comment. In response, we have revised the entire introduction to incorporate relevant theoretical and empirical literature on gender as it pertains to the research. In addition, we strengthened the literature review by including more global studies on gender and sustainable land management. In the revised manuscript, the hypotheses are now directly derived from the identified gaps following the literature review. The entire manuscript has been edited accordingly.  Please see pages 3–9, lines 76–98 for the theoretical issues, and lines 219–223 for the hypotheses. We hope this revision satisfactorily addresses your concerns. | 7 |  |  |
| 8. |  |  | 8 | In this study, I found an excellent data collection technique. However, it is critical to consider the suggestions mentioned below.   - Clarify whether the variables were statistically tested and whether the model passed standard diagnostic checks (e.g., goodness-of-fit). | We thank you for the positive comment on the quality of our data collection process, and for the valuable suggestion to clarify the statistical testing and diagnostic checks related to the model. In response, we confirm that the Tobit model was subjected to standard diagnostic procedures, which are now detailed in the revised manuscript.  Please refer to page 19, lines 406–416 for information on the model fit and diagnostic assessments. We hope this revision satisfactorily addresses your concerns. | 8 |  |  |
| 9. |  |  | 9 | Assumptions of the Tobit Model   1. Censoring mechanism is known and correctly specified: Data is censored from above, below, or both, and this is properly defined. 2. Latent variable is linearly related to predictors: The underlying latent variable (unobserved variable) follows a linear model. 3. Normality of errors: The error term follows a normal distribution (Gaussian). 4. Homoscedasticity: Constant variance of error terms across values of independent variables. 5. Independence: Observations are independent of one another. 6. No perfect multicollinearity: Predictors are not perfectly correlated.   I suggest including evidence of a multicollinearity test for your variables and further analytic explanations regarding the employed model. Additionally, you should verify the assumptions and analytical framework for this model. | We thank you very much for the suggestions regarding the assumptions of the Tobit model. In response, we documented the nature and direction of censoring in our data in the revised manuscript. In addition, while the Tobit model assumes a linear relationship between the latent variable and the predictors, as well as normally distributed errors, prior literature indicates that the model is relatively robust to moderate deviations from normality and linearity — particularly when the primary goal is inference rather than prediction (Wooldridge, 2010).  To assess the assumption of constant error variance, we examined residual plots and conducted a Breusch-Pagan test. While the test results indicate significant heteroskedasticity, this was addressed using robust standard errors to ensure valid inference. Moreover, there are no repeated measurements or clustering present in the dataset. Finally, the choice of the model was guided by the distributional properties of the dependent variable and consistency with previous studies employing similar censored data structures.  Please refer to pages 13–21, lines 290–332 & 392–438 for details on censoring mechanism, multicollinearity diagnostics, model assumptions, and analytical framework.  We hope this revision satisfactorily addresses your concerns. | 9 |  |  |
| 10. |  |  | 10 | - The discussion section would be stronger with more references to comparative and recent literature. - Organize the section using clear thematic sub-headings (e.g., Gender Differences in SLM Training and Farm Size, SLM Training and Farm Size Impact on WTP, Gender-Specific Effects on Willingness to Pay) | We sincerely thank you for your insightful suggestions aimed at improving the quality of the discussion. In response, we fully agree that integrating comparative and recent literature is essential for situating our findings within a broader scholarly context. Accordingly, our interpretations now draw on current evidence related to the influence of SLM training, farm size, household income, livelihood diversification, and gender-specific access to resources (e.g., [8], [10], [11], [12], [15], [25], [27], [28]).  Regarding the organisation of the results and discussions, we have structured the analysis around both main and conditional effects, linking the findings explicitly to the hypotheses of the study (H1-H3) This approach creates a more coherent connection between our theoretical propositions and the empirical results, while also integrating our key thematic concerns—such as gender effects and the interaction between training and farm size—within the interpretation of each hypothesis.  Please refer to the Results and discussion section on pages 18–27, lines 392–560, for further details. We hope this revision satisfactorily addresses your concerns. | 10 |  |  |
| 11 |  |  | 11 | Conclusion   - More clearly tie the empirical findings to the broader context of gender perspectives on sustainable land management and the variable you considered. - Reiterate the importance and relevance of your variables. - Suggest actionable policy recommendations or scaling strategies for land value assessment frameworks. | We thank you for the thoughtful comments on the conclusion. In response, we have revised the entire conclusion to address the concerns raised. The revised conclusion now emphasises clearly the empirical findings, tying it to the broader context of gender perspectives on sustainable land management and the main variables we considered. In addition, we reiterate the significance of these variables in the conclusion, and made actionable policy recommendations.  Please find details on pages 28–29, lines 588–615.  We hope this revision satisfactorily addresses your concerns. | 11 |  |  |
| 12 |  |  | 12 |  |  | 12 |  |  |
| 13 |  |  | 13 |  |  | 13 |  |  |
| 14 |  |  | 14 |  |  | 14 |  |  |
| 15 |  |  | 15 |  |  | 15 |  |  |
| 16 |  |  | 16 |  |  | 16 |  |  |
| 17 |  |  | 17 |  |  | 17 |  |  |
| 18 |  |  | 18 |  |  | 18 |  |  |
| 19 |  |  | 19 |  |  | 19 |  |  |
| 20 |  |  | 20 |  |  | 20 |  |  |
| 21 |  |  | 21 |  |  | 21 |  |  |
| 22 |  |  | 22 |  |  | 22 |  |  |
| 23 |  |  | 23 |  |  | 23 |  |  |

**RESPONSE TO EDITOR’S REQUEST**

Thank you for your helpful comment. We have revised the Ethics Statement in the Methods section and the online submission information to include the following:

i. Informed verbal consent was obtained from all participants.
ii. Consent procedures were standardised, explained in local languages, and documented in field logbooks.
iii. Only adults aged 18 and above participated.
iv. No formal ethical approval was obtained at the time of data collection, but anonymity and confidentiality were strictly maintained.
v. No identifying data were collected, and all responses were anonymized.

Please find details on pages 10-11, lines 253–258.

We hope these revisions satisfactorily meet your request and align with the journal's ethical requirements.
