# Supplementary material for: Gendered dimensions of sustainable land management: Evidences from farm size and training effects on willingness to pay in Ghana’s Volta Region
Source: PLoS One. 2026 Jun 11;21(6):e0351424. doi: 10.1371/journal.pone.0351424 (PMC13257986; doi:10.1371/journal.pone.0351424)
Supplement: Attachment — Submitted filename: Gendered dimensions report 2.docx [file pone.0351424.s009.docx]

Gendered dimensions of sustainable land management: evidence of farm size and training effects on willingness to pay in Ghana’s Volta Region

PONE-D-25-40765

The reviewer report 2

**Title**

The title is not supported by the background literature. Nevertheless and according to your literature findings, you mentioned two issues (farm size and SLM training and their effects on WTP) related to your hypotheses, the gender was not mentioned. The title must reflect the main hypothesis (the research main question).

**Abstract**

You results in the abstract are not matching with what you have investigated. Focus the abstract results on findings that directly align with the stated hypothesis and objectives, with the impact of gender differences presented as the primary result. The conclusion is not related very much to the study results or to the hypotheses..

**Introduction**

The introduction should concisely review both theoretical and empirical literature on gender relevant to the research questions. Strengthen the literature review with additional gender studies from diverse African or international contexts. The hypothesis should be grounded in strong evidence from this literature review. The section needs a language revision.

**Methodology**

Review the methodology to determine if it is appropriate for the study population, considering cluster or stratified methods. Remove the extraneous information about measured variables or other wise move them to the relevant section. Focus the study model description on the specific methods used, removing well-known equations. Reorganize the section for clarity and use scientific language.

**Results**

Clearly present the results, addressing each research question. Name the tables and refer to them in the appropriate position. Avoid explaining methodology; use direct, simple scientific language to clarify findings.

**Acknowledgement**

Acknowledgments usually thank professional, academic, and personal support, progressing from formal (supervisor, funding bodies) to informal (peers, family, friends).

Accordingly directly acknowledge the persons who help you.
