# Supplementary material for: Gendered dimensions of sustainable land management: Evidences from farm size and training effects on willingness to pay in Ghana’s Volta Region
Source: PLoS One. 2026 Jun 11;21(6):e0351424. doi: 10.1371/journal.pone.0351424 (PMC13257986; doi:10.1371/journal.pone.0351424)
Supplement: Attachment — Submitted filename: Reviewer Feedback Round 2.docx [file pone.0351424.s011.docx]

**Feedback from the Reviewer**

Minor Revision

The manuscript comprehensively addresses the corrections.

The manuscript (i.e., the written document or paper) has **fully and thoroughly responded to or incorporated** the corrections or feedback that were previously given.

However, including the DOIs of the cited articles would enhance the rigor of the manuscript and make it more appealing to readers. Therefore, I kindly ask that the DOI for each referenced article be included in the manuscript.
