# Supplementary material for: Gendered dimensions of sustainable land management: Evidences from farm size and training effects on willingness to pay in Ghana’s Volta Region
Source: PLoS One. 2026 Jun 11;21(6):e0351424. doi: 10.1371/journal.pone.0351424 (PMC13257986; doi:10.1371/journal.pone.0351424)
Supplement: Attachment — Submitted filename: RESPONSE_TO_REVIEWERS_COMMENTS_auresp_2.docx [file pone.0351424.s012.docx]

8^th^ January, 2026

Dear Editor-in-Chief

**Subject: Revision and Resubmission of Manuscript Number:  PONE-D-25-40765R1**

We are grateful for the opportunity to address several minor issues related to clarity, language, and presentation, and resubmit our manuscript titled "***Gendered dimensions of sustainable land management: evidence of farm size and training effects on willingness to pay in Ghana’s Volta Region***" for consideration in PLOS ONE.

The responses to each comment in the revised manuscript are summarised in the table below, providing a point-by-point explanation of how the concerns were resolved. All co-authors have reviewed and approved the revised manuscript, and I remain the corresponding author for this submission.

Thank you once again for your time and consideration.

Sincerely,

James Dickson Fiagborlo (Ph.D.),

Faculty of Applied Social Sciences,

Department of Multidisciplinary Studies,

Ho Technical University,

Ho, Ghana.

**Corresponding Author**

| **SN** | **1^st^ Reviewer’s comment** | **Author’s response to reviewer 1** | **SN** | **2^nd^ Reviewer’s comment** | **Author’s response to reviewer 2** | **SN** | **3^rd^ Reviewer’s comment** | **Author’s response to reviewer 3** |
| --- | --- | --- | --- | --- | --- | --- | --- | --- |
| 1. | The title is not supported by the background literature. Nevertheless, and according to your literature findings, you mentioned two issues (farm size and SLM training and their effects on WTP) related to your hypotheses, the gender was not mentioned. The title must reflect the main hypothesis (the research main question). | Thank you for your thoughtful comment regarding the alignment between the manuscript’s title and its background literature. In response, we revised the background section of the manuscript to explicitly incorporate gender as a core analytical dimension. The revised section now clearly articulates the role of gender in shaping farmers’ willingness to pay for Sustainable Land Management practices, alongside farm size and training–thereby providing stronger justification for the inclusion of gender in the study’s hypotheses and title.  In particular, we have added relevant literature that discusses gender differences in agricultural decision-making, access to productive resources, and adoption of sustainable practices. These additions provide a clearer conceptual foundation for the gender-related hypothesis and reinforce the relevance of gender in the analysis.  Please refer to page 4, lines 76-86 for the detailed revisions  We hope this revision addresses your concern. Thank you again for valuable feedback. | 1 | The manuscript (i.e., the written document or paper) has fully and thoroughly responded to or incorporated the corrections or feedback that were previously given. However, including the DOIs of the cited articles would enhance the rigor of the manuscript and make it more appealing to readers. Therefore, I kindly ask that the DOI for each referenced article be included in the manuscript. | Thank you for your positive feedback and for acknowledging the revisions made to address the earlier comments. In response to your suggestions, the revised manuscript now include DOIs for the majority of the cited papers in the reference list. Where a DOI was not available, full citation details have been provided to maintain traceability. For details, please refer to the reference list. We hope this addresses your concern. Thanks | 1 | I have no further suggestions, The author should further emphasize the significance of the research and highlight the core value of this study. | Thanks for your thoughtful feedback. The revised manuscript already emphasised the significance and core contribution of the study in the introduction section. In particular, the study highlights three core areas: (1) It provides empirical evidence on how gender dynamics, training, and farm size interact to influence sustainability-oriented behaviour among farming households in Ghana; (2) It applies a gender-sensitive analytical framework to assess willingness to pay for Sustainable Land Management practices, contributing to the broader literature on environmental and development economics; (3) It offers policy-relevant insights to inform gender-equitable agricultural extension, land management, and climate adaptation strategies, supporting more inclusive and sustainable rural development. For more details, please refer to page 5, lines 112 -118.  I hope this satisfactorily addresses your concerns. |
| 2. | You result in the abstract are not matching with what you have investigated. Focus the abstract results on findings that directly align with the stated hypothesis and objectives, with the impact of gender differences presented as the primary result. The conclusion is not related very much to the study results or to the hypotheses.. | Thank you very much for this insightful comment. In response, we have revised the abstract to explicitly link the study’s key findings to its stated objectives and hypotheses. The clarification strengthens the internal coherence of the paper  The revisions can be found on page 2, lines 34–46.  We hope that our response addresses your concerns satisfactorily. | 2 |  |  |  |  |  |
| 3. | The introduction should concisely review both theoretical and empirical literature on gender relevant to the research questions. Strengthen the literature review with additional gender studies from diverse African or international contexts. The hypothesis should be grounded in strong evidence from this literature review. The section needs a language revision. | We are grateful for this insightful comment. In response, we have revised the manuscript to incorporate relevant theoretical and empirical literature on gender as it pertains to the research. Specifically, we strengthened the literature review by including additional global studies that highlight gender and sustainable land management.  Furthermore, in the revised manuscript, the hypotheses are now directly derived from the identified gaps following the literature review. The entire manuscript has been edited accordingly.  Please refer to pages 4–10, lines 76–97 for the theoretical discussion, and lines 219–225 for the hypotheses. We hope these revisions satisfactorily addresses your concerns. We appreciate your valuable feedback | 3 |  |  |  |  |  |
| 4. | Review the methodology to determine if it is appropriate for the study population, considering cluster or stratified methods. Remove the extraneous information about measured variables or otherwise move them to the relevant section. Focus the study model description on the specific methods used, removing well-known equations. Reorganize the section for clarity and use scientific language. | Thank you for your kind comments and helpful suggestions. In response, we have revised the entire methodology section to address the concerns raised.  In particular, the revised section now clearly outlines the study design and sampling procedures. We have also refined the description of the models, focusing on the specific methods used in the study and removing standard or widely known equations to enhance clarity.  Additionally, we have edited the entire methodology section for consistency in scientific language and overall clarity.  Please refer to pages 10–14, lines 228–239 for details on the study design and sampling procedure, and lines 260–314 for the information on the measured variables and model specification.  We hope this revisions satisfactorily address your concerns and improve the quality of the manuscript. | 4 |  |  |  | . |  |
| 5. | Clearly present the results, addressing each research question. Name the tables and refer to them in the appropriate position. Avoid explaining methodology; use direct, simple scientific language to clarify findings. | We are grateful for your insightful feedback. In response to your comments, we have revised the entire Results and Discussion section of the manuscript. Specifically, the empirical findings are now structured to distinguish between the main effects and conditional effects of the predictor variables. We have improved the scientific clarity of the language used to ensure that each finding is explicitly linked to the study’s hypotheses.  While we were unable to adopt the reference style you suggested, we have maintained consistency and clarity in citations throughout the section.  Please refer to pages 18–28, lines 392–553 for the detailed revisions. We hope these revisions meet your expectations and satisfactorily address your concerns. | 5 |  |  |  |  |  |
| 6. | Acknowledgments usually thank professional, academic, and personal support, progressing from formal (supervisor, funding bodies) to informal (peers, family, friends). Accordingly, directly acknowledge the persons who help you. | We sincerely thank you for your helpful suggestions and fully agree with your insightful observations. In response, we have revised the acknowledgements section to reflect your feedback.  Please refer to page 28, lines 584–586 for the updated text. We hope this revision satisfactorily addresses your concerns. |  |  |  |  |  |  |

**RESPONSE TO EDITOR’S REQUEST**

Thank you for your valuable comment regarding repetition in the manuscript. In response, we have revised the sections on Main effects, Interaction effects, and Total marginal effects to reduce redundancy while maintaining analytical clarity. Overlapping explanations and repetitive interpretations have been streamlined, and transitions between sections have been improved to ensure coherence and logical flow.

The revised version now presents each analytical component distinctly and succinctly, in line with the Journal’s editorial standards and to enhance overall readability.

For further details, please refer to pages 21-26, lines 441-527 of the revised manuscript.
