# Supplementary material for: Gendered dimensions of sustainable land management: Evidences from farm size and training effects on willingness to pay in Ghana’s Volta Region
Source: PLoS One. 2026 Jun 11;21(6):e0351424. doi: 10.1371/journal.pone.0351424 (PMC13257986; doi:10.1371/journal.pone.0351424)
Supplement: Attachment — Submitted filename: Gendered dimensions report 3.docx [file pone.0351424.s014.docx]

Gendered dimensions of sustainable land management: evidence of farm size and training effects on willingness to pay in Ghana’s Volta Region

PONE-D-25-40765R2

The reviewer report 3

**Abstract**

The abstract's results should directly reflect the stated hypothesis and objectives, emphasizing the impact of gender differences as the primary finding. Revise the abstract conclusion to align more closely with the study's results and hypotheses.

**Literature**

The hypotheses do not align with the study title. The study investigates gender dimensions using farm size and training effects, but the hypotheses focus on how farm size and training affect willingness to pay (WTP).

**Methodology**

Review the methodology to determine if the methods are appropriate for the study population, considering cluster or stratified approaches. Focus the study model description on the specific methods used, moving extraneous information about measured variables to the relevant section.

**Results**

Clearly present the results, addressing each research question. Name and reference tables appropriately. State findings directly and concisely, avoiding methodological explanations.

**Conclusion**

The main concluding points must be focus on the key question of the study (Gender).

**Acknowledgement**

Acknowledgments typically express gratitude for professional, academic, and personal support received during the (original) work, progressing from formal acknowledgments (supervisors, funding) to informal ones (peers, family, friends). Directly acknowledge those who provided assistance.
