# Supplementary material for: Gendered dimensions of sustainable land management: Evidences from farm size and training effects on willingness to pay in Ghana’s Volta Region
Source: PLoS One. 2026 Jun 11;21(6):e0351424. doi: 10.1371/journal.pone.0351424 (PMC13257986; doi:10.1371/journal.pone.0351424)
Supplement: Attachment — Submitted filename: Authors response to reviewes.docx [file pone.0351424.s015.docx]

24^th^ April, 2026

Dear Editor-in-Chief

**Subject: Revision and Resubmission of Manuscript Number:  PONE-D-25-40765R2**

We are grateful for the opportunity to address several minor issues related to clarity, language, and presentation, and resubmit our manuscript titled "***Gendered dimensions of sustainable land management: evidences of farm size and training effects on willingness to pay in Ghana’s Volta Region***" for consideration in PLOS ONE.

The responses to each comment in the revised manuscript are summarised in the table below, providing a point-by-point explanation of how the concerns were resolved. All co-authors have reviewed and approved the revised manuscript, and I remain the corresponding author for this submission.

Thank you once again for your time and consideration.

Sincerely,

James Dickson Fiagborlo (Ph.D.),

Faculty of Applied Social Sciences,

Department of Multidisciplinary Studies,

Ho Technical University,

Ho, Ghana.

**Corresponding Author**

| **SN** | **1^st^ Reviewer’s comment** | **Authors’ response to reviewer 1** | **SN** | **3^rd^ Reviewer’s comment** | **Authors’ response to reviewer 3** |  | **Editor’s comment** | **Authors’ response to Editor** |
| --- | --- | --- | --- | --- | --- | --- | --- | --- |
| 1. | The abstract's results should directly reflect the stated hypothesis and objectives, emphasizing the impact of gender differences as the primary finding. Revise the abstract conclusion to align more closely with the study's results and hypotheses. | Thank you for your thoughtful comment, which has helped us improved the clarity and alignment of our abstract.  In response, we have reviewed the results part of the abstract to ensure that it directly reflects the study’s stated hypotheses and objectives. The revised abstract now foregrounds the association between gender and the key outcome, while secondary findings are limited to the results and discussion section of the manuscript.  Please refer to page 1, lines 34-45 for the detailed revisions.  We hope this revision addresses your concern. Thank you again for your valuable feedback. | 1 | I have no further suggestions. The author has already addressed all the issues. It is suggested that the author further revise the entire text and minimize the number of grammatical errors as much as possible. | We sincerely thank the reviewer for the positive evaluation of our manuscript and for recognising that the major concerns have been adequately addressed.  In response to your+ suggestions, we have carefully revised the entire manuscript to minimise grammatical errors and improve all clarity and readability. The text has been thoroughly edited for language consistency, sentence structure, and academic tone. Where necessary, sentences have been rephrased to enhance precision and flow.  Please refer to introduction to conclusion of the manuscript for details.  We hope this satisfactorily addresses your concerns. | 1 | The manuscript has improved after revision and is now much stronger. However, a few minor revisions are still needed. Please reduce repetition (particularly in the DiD explanation), simplify some of the longer sentences, and more clearly state the main contribution of the study in the introduction. In addition, tighten the discussion of results by focusing on key findings, include a brief limitations paragraph, and ensure consistent terminology throughout the paper. Please also note that one reviewer recommended rejection. We ask that you carefully address all reviewer comments and provide a clear, point-by-point response explaining how the manuscript has been revised in light of their concerns. | We sincerely thank the reviewer for the encouraging assessment of our revised manuscript and for the constructive suggestions to further strengthen the paper. We also appreciate the editorial guidance and have carefully revised the manuscript to address each of the remaining concerns.  In response to the specific comment, we have reduced repetition throughout the manuscript, particularly in the explanation of the DiD, to improve conciseness and readability. We have also simplified several long or complex sentences to enhance clarity. In the introduction, we now state the main contribution of the study more explicitly so that the novelty and significance of the work are clear from the outset. In addition, we have revised the Discussion section to focus more tightly on the key findings, added a brief paragraph describing the study limitations at the end of the conclusions, and reviewed the full manuscript to ensure consistent terminology across all sections.  We hope the revisions satisfactorily addresses your concerns.  Please refer to pages 5, 15-24, lines 108 -119, 322-500, and 494-502 for more details.  We are grateful for this thoughtful feedback. |
| 2. | The hypotheses do not align with the study title. The study investigates gender dimensions using farm size and training effects, but the hypotheses focus on how farm size and training affect willingness to pay (WTP). | Thank you very much for this insightful observation regarding the alignment between the study title and the stated hypotheses. We acknowledge that the original hypotheses were primarily framed to assess the effects of farm size and SLM training on willingness to pay, without explicitly adding the gender dimension highlighted in the study title.  In response to your comment, we have revised the hypotheses to explicitly reflect gender differences and interactions, thereby ensuring consistency with the study’s objectives and framing. We also thematically reviewed the entire literature section to ensure that the hypotheses derive and align properly with existing literature.  The revisions can be found on pages 6–9, lines 125–217.  We hope that our response addresses your concerns satisfactorily. |  |  |  |  |  |  |
| 3. | Review the methodology to determine if the methods are appropriate for the study population, considering cluster or stratified approaches. Focus the study model description on the specific methods used, moving extraneous information about measured variables to the relevant section.. | We thank the reviewer for this thoughtful and constructive comment. This has helped us improve both the methodological rigor and the clarity of our manuscript.  In response, we have carefully revised our methodology to ensure that it appropriately reflects the structure of the study population. Given the presence of potential clustering at the community/village/household level, we describe stratification procedures used during sampling and analysis. We further refined the model description to focus exclusively on the analytical methods used, removing descriptive details about measured variables and relocating them to a dedicated section. These revisions improve both the methodological transparency and structural clarity of the manuscript. Please refer to pages 9–13, lines 219–304 for the methodological and analytical model.  We hope these revisions satisfactorily addresses your concerns. We appreciate your valuable feedback |  |  |  |  |  |  |
| 4. | Clearly present the results, addressing each research question. Name and reference tables appropriately. State findings directly and concisely, avoiding methodological explanations. | We thank the reviewer for this insightful comment. It has helped us improve the clarity and organisation of the Results section.  In response, we have revised the Results section to explicitly address each research hypothesis or question in a structured manner. Findings are now presented sequentially, with each section corresponding to a specific research hypothesis or objective. We have also ensured that all tables are clearly titled, numbered in order, and appropriately referenced within the text. Additionally, we have refined the language throughout the Results section to state findings directly and concisely, removing methodological explanations that are more appropriately placed in the methods section. These revisions ensure that the Results section is clearly structured, directly addresses the research questions, and is presented in concise and reader-friendly manner.  Please refer to pages 19–24, lines 393–475 for details.  We appreciate your valuable feedback. |  |  |  |  |  |  |
| 5. | The main concluding points must be focus on the key question of the study (Gender). | We thank you for your kind comments and helpful suggestion. It has helped us strengthen the focus of our manuscript.  In response, we have revised the conclusion to ensure that it clearly emphasises the central role of gender in our study. In particular, the concluding statements now directly reflect the key findings related to gender differences and their implications, including alignment with the study’s objectives and results. These revisions ensure that the conclusion is clearly centered on the study’s primary focus (gender) and that the main findings are presented in direct relation to this key research question.  Please refer to pages 23–24, lines 477–502 for details.  We hope this revisions satisfactorily address your concerns and improve the quality of the manuscript. |  | . |  |  |  |  |
| 6. | Acknowledgments typically express gratitude for professional, academic, and personal support received during the (original) work, progressing from formal acknowledgments (supervisors, funding) to informal ones (peers, family, friends). Directly acknowledge those who provided assistance. | We thank you for this helpful suggestions, which has improved the completeness and professionalism of our manuscript.  In response, we have revised the Acknowledgements section to clearly and appropriately recognise all sources of support, progressing from formal academic and instructional contributions to personal acknowledgements. We have also ensured that all individuals and organisations who provided direct assistance are explicitly acknowledged.  These revisions ensure that Acknowledgements section aligns with standard scholarly conventions and appropriately recognises all contributions.  Please refer to page 24, lines 504–508 for the updated text. We hope this revision satisfactorily addresses your concerns. |  |  |  |  |  |  |
