# Supplementary material for: Gendered dimensions of sustainable land management: Evidences from farm size and training effects on willingness to pay in Ghana’s Volta Region
Source: PLoS One. 2026 Jun 11;21(6):e0351424. doi: 10.1371/journal.pone.0351424 (PMC13257986; doi:10.1371/journal.pone.0351424)
Supplement: Peer review history

# Peer review: Decision Letter 0

**DOI:** 10.1371/journal.pone.0351424.r001

**Authors:** Muhammad Umer Arshad

20 Oct 2025

PONE-D-25-40765

Gendered dimensions of sustainable land management: evidence of farm size and training effects on willingness to pay in Ghana’s Volta Region

PLOS ONE

Dear Dr. Fiagborlo,

Thank you for submitting your manuscript to PLOS ONE. After careful consideration, we feel that it has merit but does not fully meet PLOS ONE’s publication criteria as it currently stands. Therefore, we invite you to submit a revised version of the manuscript that addresses the points raised during the review process.

Please submit your revised manuscript by Dec 04 2025 11:59PM. If you will need more time than this to complete your revisions, please reply to this message or contact the journal office at plosone@plos.org. When you're ready to submit your revision, log on to https://www.editorialmanager.com/pone/ and select the 'Submissions Needing Revision' folder to locate your manuscript file.

Please include the following items when submitting your revised manuscript:

- A rebuttal letter that responds to each point raised by the academic editor and reviewer(s). You should upload this letter as a separate file labeled 'Response to Reviewers'.
- A marked-up copy of your manuscript that highlights changes made to the original version. You should upload this as a separate file labeled 'Revised Manuscript with Track Changes'.
- An unmarked version of your revised paper without tracked changes. You should upload this as a separate file labeled 'Manuscript'.

If you would like to make changes to your financial disclosure, please include your updated statement in your cover letter. Guidelines for resubmitting your figure files are available below the reviewer comments at the end of this letter.

If applicable, we recommend that you deposit your laboratory protocols in protocols.io to enhance the reproducibility of your results. Protocols.io assigns your protocol its own identifier (DOI) so that it can be cited independently in the future. For instructions see: https://journals.plos.org/plosone/s/submission-guidelines#loc-laboratory-protocols. Additionally, PLOS ONE offers an option for publishing peer-reviewed Lab Protocol articles, which describe protocols hosted on protocols.io. Read more information on sharing protocols at https://plos.org/protocols?utm_medium=editorial-email&utm_source=authorletters&utm_campaign=protocols.

We look forward to receiving your revised manuscript.

Kind regards,

Muhammad Umer Arshad

Academic Editor

PLOS ONE

**Journal Requirements:**

1. When submitting your revision, we need you to address these additional requirements.

Please ensure that your manuscript meets PLOS ONE's style requirements, including those for file naming. The PLOS ONE style templates can be found at

https://journals.plos.org/plosone/s/file?id=wjVg/PLOSOne_formatting_sample_main_body.pdf and

https://journals.plos.org/plosone/s/file?id=ba62/PLOSOne_formatting_sample_title_authors_affiliations.pdf

2. Please provide additional details regarding participant consent. In the ethics statement in the Methods and online submission information, please ensure that you have specified (a) whether consent was informed and (b) what type you obtained (for instance, written or verbal, and if verbal, how it was documented and witnessed). If your study included minors, state whether you obtained consent from parents or guardians. If the need for consent was waived by the ethics committee, please include this information.

If you are reporting a retrospective study of medical records or archived samples, please ensure that you have discussed whether all data were fully anonymized before you accessed them and/or whether the IRB or ethics committee waived the requirement for informed consent. If patients provided informed written consent to have data from their medical records used in research, please include this information.

3. Thank you for stating the following in the Acknowledgments Section of your manuscript:

The data for this paper came from a bigger study funded by USAID's Agric Sector Policy Support Project (APSP) (Grant APSP-STS-012). The project was concluded, and the data, which was fully anonymised with no identifiable human subjects, has been archived according to APSP regulations. We thank our most valued respondents for their insights and time.

We note that you have provided funding information that is not currently declared in your Funding Statement. However, funding information should not appear in the Acknowledgments section or other areas of your manuscript. We will only publish funding information present in the Funding Statement section of the online submission form.

Please remove any funding-related text from the manuscript and let us know how you would like to update your Funding Statement. Currently, your Funding Statement reads as follows:

The author(s) received no specific funding for this work.

Please include your amended statements within your cover letter; we will change the online submission form on your behalf.

4. We note that you have indicated that there are restrictions to data sharing for this study. For studies involving human research participant data or other sensitive data, we encourage authors to share de-identified or anonymized data. However, when data cannot be publicly shared for ethical reasons, we allow authors to make their data sets available upon request. For information on unacceptable data access restrictions, please see http://journals.plos.org/plosone/s/data-availability#loc-unacceptable-data-access-restrictions.

Before we proceed with your manuscript, please address the following prompts:

a) If there are ethical or legal restrictions on sharing a de-identified data set, please explain them in detail (e.g., data contain potentially identifying or sensitive patient information, data are owned by a third-party organization, etc.) and who has imposed them (e.g., a Research Ethics Committee or Institutional Review Board, etc.). Please also provide contact information for a data access committee, ethics committee, or other institutional body to which data requests may be sent.

b) If there are no restrictions, please upload the minimal anonymized data set necessary to replicate your study findings to a stable, public repository and provide us with the relevant URLs, DOIs, or accession numbers. Please see http://www.bmj.com/content/340/bmj.c181.long for guidelines on how to de-identify and prepare clinical data for publication. For a list of recommended repositories, please see https://journals.plos.org/plosone/s/recommended-repositories. You also have the option of uploading the data as Supporting Information files, but we would recommend depositing data directly to a data repository if possible.

Please update your Data Availability statement in the submission form accordingly.

5. We note that you have referenced (Edkins M.) which has currently not yet been accepted for publication. Please remove this from your References and amend this to state in the body of your manuscript: (Edkins M. [Unpublished]) as detailed online in our guide for authors http://journals.plos.org/plosone/s/submission-guidelines#loc-reference-style

6. If the reviewer comments include a recommendation to cite specific previously published works, please review and evaluate these publications to determine whether they are relevant and should be cited. There is no requirement to cite these works unless the editor has indicated otherwise.

[Note: HTML markup is below. Please do not edit.]

Reviewers' comments:

Reviewer's Responses to Questions

**Comments to the Author**

1. Is the manuscript technically sound, and do the data support the conclusions?

The manuscript must describe a technically sound piece of scientific research with data that supports the conclusions. Experiments must have been conducted rigorously, with appropriate controls, replication, and sample sizes. The conclusions must be drawn appropriately based on the data presented.

Reviewer #1: No

Reviewer #2: Partly

Reviewer #3: Partly

**********

2. Has the statistical analysis been performed appropriately and rigorously?

Reviewer #1: No

Reviewer #2: Yes

Reviewer #3: No

**********

3. Have the authors made all data underlying the findings in their manuscript fully available?

The PLOS Data policy requires authors to make all data underlying the findings described in their manuscript fully available without restriction, with rare exception (please refer to the Data Availability Statement in the manuscript PDF file). The data should be provided as part of the manuscript or its supporting information, or deposited to a public repository. For example, in addition to summary statistics, the data points behind means, medians and variance measures should be available. If there are restrictions on publicly sharing data—e.g. participant privacy or use of data from a third party—those must be specified.

Reviewer #1: No

Reviewer #2: Yes

Reviewer #3: Yes

**********

4. Is the manuscript presented in an intelligible fashion and written in standard English?

PLOS ONE does not copyedit accepted manuscripts, so the language in submitted articles must be clear, correct, and unambiguous. Any typographical or grammatical errors should be corrected at revision, so please note any specific errors here.

Reviewer #1: No

Reviewer #2: Yes

Reviewer #3: Yes

**********

5. Review Comments to the Author

Please use the space provided to explain your answers to the questions above. You may also include additional comments for the author, including concerns about dual publication, research ethics, or publication ethics. (Please upload your review as an attachment if it exceeds 20,000 characters)

**Reviewer #1:**Gendered dimensions of sustainable land management: evidence of farm size and training effects on willingness to pay in Ghana’s Volta Region

PONE-D-25-40765

The reviewer report

Abstract

Provide a concise background statement to contextualize the study and highlight its significance. Expand the methodology section with additional details. Focus the abstract results on findings that directly align with the stated hypothesis and objectives, with the impact of gender differences presented as the primary result. Emphasize the study's main results in relation to the problem statement.

Introduction

In a single section, the introduction should concisely review both theoretical and empirical literature on gender relevant to the research questions. Bolster the literature review with additional gender studies from diverse African or international contexts. The hypothesis should be grounded in strong evidence from this literature review. The section needs editing and language revision.

Methodology

Review the methodology to determine if it is appropriate for the study population, considering cluster or stratified methods. Move extraneous information about measured variables to the introduction or other relevant sections. Focus the study model description on the specific methods used, removing well-known equations. Reorganize the section for clarity and use scientific language.

Results

Clearly present the results, addressing each research question. Reference relevant tables and figures at the end of the statement or paragraph. Avoid explaining methodology; use direct, simple scientific language to clarify findings.

Acknowledgement

Acknowledgments usually thank professional, academic, and personal support, progressing from formal (supervisor, funding bodies) to informal (peers, family, friends).

Accordingly, directly acknowledge the persons who help you.

**Reviewer #2:** I have attached the document that consists of the comments and recommendations for the manuscript titled 'Gendered dimensions of sustainable land management: evidence of farm size and training effects on willingness to pay in Ghana’s Volta Region.'

**Reviewer #3:** Comments

this paper addresses a topic of clear academic and policy relevance. The manuscript demonstrates the author’s solid effort in data collection and preliminary modeling. However, several important issues need to be addressed before the paper can reach publishable quality. The comments below aim to provide constructive feedback to help strengthen the theoretical framing, empirical rigor, and interpretative coherence of the study.

1. Issues with model specification and interpretation.

There are significant problems in the specification and interpretation of the regression model. The author includes both the main effects and the interaction terms in the same model, yet interprets the insignificance of a main effect as evidence of “no impact” on the dependent variable. This interpretation is econometrically invalid. When an interaction term is included, the coefficient of the main effect represents only the marginal effect when the moderator takes the value of zero (or its reference category). The total effect is the combination of the main and interaction effects. Consequently, a previously significant main effect may become statistically insignificant once its interaction with the moderator is introduced—without implying the absence of influence. The current interpretation therefore risks misrepresenting variable importance and overlooking the mechanisms implied by the interaction term, suggesting that the model specification itself is conceptually flawed.

2. Weak linkage between the central argument and empirical analysis.

There is a clear disconnection between the paper’s stated research focus and the empirical implementation. Although the title highlights a gender dimension, this theme is neither theoretically developed nor consistently integrated throughout the manuscript. The theoretical framework does not adequately explain why or how gender functions as a key analytical dimension. In the empirical section, gender appears only as a control variable or as part of an interaction term, without substantive discussion of gender-specific mechanisms. Moreover, the conclusion fails to return to the gender perspective to extract policy or theoretical implications. This disjunction weakens the thematic coherence of the study and diminishes its originality and theoretical contribution.

3. Inconsistencies between empirical findings and conclusions.

There appear to be discrepancies between the reported empirical results and the conclusions drawn from them. These inconsistencies should be carefully checked and clarified to ensure internal coherence and the credibility of the findings.

4. Insufficient empirical depth and robustness.

The empirical analysis lacks sufficient depth and rigor. The current presentation of baseline regressions represents only an initial step. To establish the robustness of the findings, the author should conduct additional robustness checks—such as alternative variable measurements, modified model specifications, or sub-sample analyses. Furthermore, heterogeneity analyses could yield richer insights by examining whether the key relationships differ across groups (beyond gender, e.g., by region, age, or other socio-economic characteristics). Most importantly, potential endogeneity issues—such as omitted variables or reverse causality—must be acknowledged and addressed, for instance through instrumental variable approaches. Without these standard robustness and validity tests, the credibility and scholarly value of the results remain limited.

**********

6. PLOS authors have the option to publish the peer review history of their article (what does this mean?). If published, this will include your full peer review and any attached files.

If you choose “no”, your identity will remain anonymous but your review may still be made public.

**Do you want your identity to be public for this peer review?** For information about this choice, including consent withdrawal, please see our Privacy Policy.

Reviewer #1: **Yes:**Abdoelmoniem Awad Attaelmanan

Reviewer #2: No

Reviewer #3: No

**********

[NOTE: If reviewer comments were submitted as an attachment file, they will be attached to this email and accessible via the submission site. Please log into your account, locate the manuscript record, and check for the action link "View Attachments". If this link does not appear, there are no attachment files.]

To ensure your figures meet our technical requirements, please review our figure guidelines: https://journals.plos.org/plosone/s/figures

You may also use PLOS’s free figure tool, NAAS, to help you prepare publication quality figures: https://journals.plos.org/plosone/s/figures#loc-tools-for-figure-preparation.

NAAS will assess whether your figures meet our technical requirements by comparing each figure against our figure specifications.

Attachment Submitted filename: PONE-D-25-40765_reviewer comments edg.pdf

Attachment Submitted filename: Gendered dimensions report 1.docx

Attachment Submitted filename: PONE-D-25-40765-reviewer comments and suggestions.docx

Attachment Submitted filename: Comments.docx

# Author comment: Author response to Decision Letter 1

**DOI:** 10.1371/journal.pone.0351424.r002

3 Dec 2025

We are grateful for this insightful comment. In response, we have revised the entire introduction to incorporate relevant theoretical and empirical literature on gender as it pertains to the research. In addition, we strengthened the literature review by including more global studies on gender and sustainable land management. In the revised manuscript, the hypotheses are now directly derived from the identified gaps following the literature review. The entire manuscript has been edited accordingly.

Please see pages 3–9, lines 76–97 for the theoretical issues, and lines 219–225 for hypotheses. We hope this revision satisfactorily addresses your concerns.

Attachment Submitted filename: RESPONSE TO REVIEWERS COMMENTS.docx

# Peer review: Decision Letter 1

**DOI:** 10.1371/journal.pone.0351424.r003

**Authors:** Muhammad Umer Arshad

11 Jan 2026

PONE-D-25-40765R1

Gendered dimensions of sustainable land management: evidence of farm size and training effects on willingness to pay in Ghana’s Volta Region

PLOS One

Dear Dr. Fiagborlo,

Thank you for submitting your manuscript to PLOS ONE. After careful consideration, we feel that it has merit but does not fully meet PLOS ONE’s publication criteria as it currently stands. Therefore, we invite you to submit a revised version of the manuscript that addresses the points raised during the review process.

Please submit your revised manuscript by Feb 25 2026 11:59PM. If you will need more time than this to complete your revisions, please reply to this message or contact the journal office at plosone@plos.org. When you're ready to submit your revision, log on to https://www.editorialmanager.com/pone/ and select the 'Submissions Needing Revision' folder to locate your manuscript file.

Please include the following items when submitting your revised manuscript:

- A letter that responds to each point raised by the academic editor and reviewer(s). You should upload this letter as a separate file labeled 'Response to Reviewers'.
- A marked-up copy of your manuscript that highlights changes made to the original version. You should upload this as a separate file labeled 'Revised Manuscript with Track Changes'.
- An unmarked version of your revised paper without tracked changes. You should upload this as a separate file labeled 'Manuscript'.

-->If you would like to make changes to your financial disclosure, please include your updated statement in your cover letter. Guidelines for resubmitting your figure files are available below the reviewer comments at the end of this letter.

If applicable, we recommend that you deposit your laboratory protocols in protocols.io to enhance the reproducibility of your results. Protocols.io assigns your protocol its own identifier (DOI) so that it can be cited independently in the future. For instructions see: https://journals.plos.org/plosone/s/submission-guidelines#loc-laboratory-protocols. Additionally, PLOS ONE offers an option for publishing peer-reviewed Lab Protocol articles, which describe protocols hosted on protocols.io. Read more information on sharing protocols at https://plos.org/protocols?utm_medium=editorial-email&utm_source=authorletters&utm_campaign=protocols.

We look forward to receiving your revised manuscript.

Kind regards,

Muhammad Umer Arshad

Academic Editor

PLOS One

Journal Requirements:

If the reviewer comments include a recommendation to cite specific previously published works, please review and evaluate these publications to determine whether they are relevant and should be cited. There is no requirement to cite these works unless the editor has indicated otherwise.

Please review your reference list to ensure that it is complete and correct. If you have cited papers that have been retracted, please include the rationale for doing so in the manuscript text, or remove these references and replace them with relevant current references. Any changes to the reference list should be mentioned in the rebuttal letter that accompanies your revised manuscript. If you need to cite a retracted article, indicate the article’s retracted status in the References list and also include a citation and full reference for the retraction notice.

Additional Editor Comments:

The manuscript has been improved and the authors have addressed the reviewers’ comments. However, before the paper can be accepted, the authors should address several minor issues related to clarity, language, and presentation. In particular, the authors should reduce repetition, carefully polish the language, and ensure that all remaining reviewer comments are fully addressed.

[Note: HTML markup is below. Please do not edit.]

Reviewers' comments:

Reviewer's Responses to Questions

**Comments to the Author**

1. If the authors have adequately addressed your comments raised in a previous round of review and you feel that this manuscript is now acceptable for publication, you may indicate that here to bypass the “Comments to the Author” section, enter your conflict of interest statement in the “Confidential to Editor” section, and submit your "Accept" recommendation.

Reviewer #1: (No Response)

Reviewer #2: All comments have been addressed

Reviewer #3: All comments have been addressed

**********

2. Is the manuscript technically sound, and do the data support the conclusions?

The manuscript must describe a technically sound piece of scientific research with data that supports the conclusions. Experiments must have been conducted rigorously, with appropriate controls, replication, and sample sizes. The conclusions must be drawn appropriately based on the data presented.

Reviewer #1: No

Reviewer #2: Yes

Reviewer #3: Yes

**********

3. Has the statistical analysis been performed appropriately and rigorously?

Reviewer #1: No

Reviewer #2: Yes

Reviewer #3: Yes

**********

4. Have the authors made all data underlying the findings in their manuscript fully available?

The PLOS Data policy requires authors to make all data underlying the findings described in their manuscript fully available without restriction, with rare exception (please refer to the Data Availability Statement in the manuscript PDF file). The data should be provided as part of the manuscript or its supporting information, or deposited to a public repository. For example, in addition to summary statistics, the data points behind means, medians and variance measures should be available. If there are restrictions on publicly sharing data—e.g. participant privacy or use of data from a third party—those must be specified.

Reviewer #1: No

Reviewer #2: Yes

Reviewer #3: Yes

**********

5. Is the manuscript presented in an intelligible fashion and written in standard English?

PLOS ONE does not copyedit accepted manuscripts, so the language in submitted articles must be clear, correct, and unambiguous. Any typographical or grammatical errors should be corrected at revision, so please note any specific errors here.

Reviewer #1: No

Reviewer #2: Yes

Reviewer #3: Yes

**********

6. Review Comments to the Author

Please use the space provided to explain your answers to the questions above. You may also include additional comments for the author, including concerns about dual publication, research ethics, or publication ethics. (Please upload your review as an attachment if it exceeds 20,000 characters)

Reviewer #1: Gendered dimensions of sustainable land management: evidence of farm size and training effects on willingness to pay in Ghana’s Volta Region

PONE-D-25-40765

The reviewer report 2

Title

The title is not supported by the background literature. Nevertheless and according to your literature findings, you mentioned two issues (farm size and SLM training and their effects on WTP) related to your hypotheses, the gender was not mentioned. The title must reflect the main hypothesis (the research main question).

Abstract

You results in the abstract are not matching with what you have investigated. Focus the abstract results on findings that directly align with the stated hypothesis and objectives, with the impact of gender differences presented as the primary result. The conclusion is not related very much to the study results or to the hypotheses..

Introduction

The introduction should concisely review both theoretical and empirical literature on gender relevant to the research questions. Strengthen the literature review with additional gender studies from diverse African or international contexts. The hypothesis should be grounded in strong evidence from this literature review. The section needs a language revision.

Methodology

Review the methodology to determine if it is appropriate for the study population, considering cluster or stratified methods. Remove the extraneous information about measured variables or otherwise move them to the relevant section. Focus the study model description on the specific methods used, removing well-known equations. Reorganize the section for clarity and use scientific language.

Results

Clearly present the results, addressing each research question. Name the tables and refer to them in the appropriate position. Avoid explaining methodology; use direct, simple scientific language to clarify findings.

Acknowledgement

Acknowledgments usually thank professional, academic, and personal support, progressing from formal (supervisor, funding bodies) to informal (peers, family, friends).

Accordingly, directly acknowledge the persons who help you.

Reviewer #2: The manuscript comprehensively addresses the corrections.

The manuscript (i.e., the written document or paper) has fully and thoroughly responded to or incorporated the corrections or feedback that were previously given.

However, including the DOIs of the cited articles would enhance the rigor of the manuscript and make it more appealing to readers. Therefore, I kindly ask that the DOI for each referenced article be included in the manuscript.

Reviewer #3: I have no further suggestions, The author should further emphasize the significance of the research and highlight the core value of this study.

**********

7. PLOS authors have the option to publish the peer review history of their article (what does this mean?). If published, this will include your full peer review and any attached files.

If you choose “no”, your identity will remain anonymous but your review may still be made public.

**Do you want your identity to be public for this peer review?** For information about this choice, including consent withdrawal, please see our Privacy Policy.

Reviewer #1: **Yes:**Abdoelmoniem Awad Attaelmanan

Reviewer #2: No

Reviewer #3: No

**********

[NOTE: If reviewer comments were submitted as an attachment file, they will be attached to this email and accessible via the submission site. Please log into your account, locate the manuscript record, and check for the action link "View Attachments". If this link does not appear, there are no attachment files.]

To ensure your figures meet our technical requirements, please review our figure guidelines: https://journals.plos.org/plosone/s/figures

You may also use PLOS’s free figure tool, NAAS, to help you prepare publication quality figures: https://journals.plos.org/plosone/s/figures#loc-tools-for-figure-preparation.

NAAS will assess whether your figures meet our technical requirements by comparing each figure against our figure specifications.

Attachment Submitted filename: Gendered dimensions report 2.docx

Attachment Submitted filename: PONE-D-25-40765_reviewer (1) comments.pdf

Attachment Submitted filename: Reviewer Feedback Round 2.docx

# Author comment: Author response to Decision Letter 2

**DOI:** 10.1371/journal.pone.0351424.r004

10 Feb 2026

In response to reviewer suggestions, the revised manuscript now include DOIs for the majority of the cited papers in the reference list. Where a DOI was not available, full citation details have been provided to maintain traceability.

Attachment Submitted filename: RESPONSE_TO_REVIEWERS_COMMENTS_auresp_2.docx

# Peer review: Decision Letter 2

**DOI:** 10.1371/journal.pone.0351424.r005

**Authors:** Muhammad Umer Arshad

17 Mar 2026

PONE-D-25-40765R2

Gendered dimensions of sustainable land management: evidence of farm size and training effects on willingness to pay in Ghana’s Volta Region

PLOS One

Dear Dr. Fiagborlo,

Thank you for submitting your manuscript to PLOS ONE. After careful consideration, we feel that it has merit but does not fully meet PLOS ONE’s publication criteria as it currently stands. Therefore, we invite you to submit a revised version of the manuscript that addresses the points raised during the review process.

Please submit your revised manuscript by May 01 2026 11:59PM. If you will need more time than this to complete your revisions, please reply to this message or contact the journal office at plosone@plos.org. When you're ready to submit your revision, log on to https://www.editorialmanager.com/pone/ and select the 'Submissions Needing Revision' folder to locate your manuscript file.

Please include the following items when submitting your revised manuscript:

- A letter that responds to each point raised by the academic editor and reviewer(s). You should upload this letter as a separate file labeled 'Response to Reviewers'.
- A marked-up copy of your manuscript that highlights changes made to the original version. You should upload this as a separate file labeled 'Revised Manuscript with Track Changes'.
- An unmarked version of your revised paper without tracked changes. You should upload this as a separate file labeled 'Manuscript'.

-->If you would like to make changes to your financial disclosure, please include your updated statement in your cover letter. Guidelines for resubmitting your figure files are available below the reviewer comments at the end of this letter.

If applicable, we recommend that you deposit your laboratory protocols in protocols.io to enhance the reproducibility of your results. Protocols.io assigns your protocol its own identifier (DOI) so that it can be cited independently in the future. For instructions see: https://journals.plos.org/plosone/s/submission-guidelines#loc-laboratory-protocols. Additionally, PLOS ONE offers an option for publishing peer-reviewed Lab Protocol articles, which describe protocols hosted on protocols.io. Read more information on sharing protocols at https://plos.org/protocols?utm_medium=editorial-email&utm_source=authorletters&utm_campaign=protocols.

We look forward to receiving your revised manuscript.

Kind regards,

Muhammad Umer Arshad

Academic Editor

PLOS One

Journal Requirements:

If the reviewer comments include a recommendation to cite specific previously published works, please review and evaluate these publications to determine whether they are relevant and should be cited. There is no requirement to cite these works unless the editor has indicated otherwise.

Please review your reference list to ensure that it is complete and correct. If you have cited papers that have been retracted, please include the rationale for doing so in the manuscript text, or remove these references and replace them with relevant current references. Any changes to the reference list should be mentioned in the rebuttal letter that accompanies your revised manuscript. If you need to cite a retracted article, indicate the article’s retracted status in the References list and also include a citation and full reference for the retraction notice.

Additional Editor Comments:

The manuscript has improved after revision and is now much stronger. However, a few minor revisions are still needed. Please reduce repetition (particularly in the DiD explanation), simplify some of the longer sentences, and more clearly state the main contribution of the study in the introduction. In addition, tighten the discussion of results by focusing on key findings, include a brief limitations paragraph, and ensure consistent terminology throughout the paper. Please also note that one reviewer recommended rejection. We ask that you carefully address all reviewer comments and provide a clear, point-by-point response explaining how the manuscript has been revised in light of their concerns.

[Note: HTML markup is below. Please do not edit.]

Reviewers' comments:

Reviewer's Responses to Questions

**Comments to the Author**

1. If the authors have adequately addressed your comments raised in a previous round of review and you feel that this manuscript is now acceptable for publication, you may indicate that here to bypass the “Comments to the Author” section, enter your conflict of interest statement in the “Confidential to Editor” section, and submit your "Accept" recommendation.

Reviewer #1: (No Response)

Reviewer #3: All comments have been addressed

**********

2. Is the manuscript technically sound, and do the data support the conclusions?

The manuscript must describe a technically sound piece of scientific research with data that supports the conclusions. Experiments must have been conducted rigorously, with appropriate controls, replication, and sample sizes. The conclusions must be drawn appropriately based on the data presented.

Reviewer #1: No

Reviewer #3: Yes

**********

3. Has the statistical analysis been performed appropriately and rigorously?

Reviewer #1: No

Reviewer #3: Yes

**********

4. Have the authors made all data underlying the findings in their manuscript fully available?

The PLOS Data policy requires authors to make all data underlying the findings described in their manuscript fully available without restriction, with rare exception (please refer to the Data Availability Statement in the manuscript PDF file). The data should be provided as part of the manuscript or its supporting information, or deposited to a public repository. For example, in addition to summary statistics, the data points behind means, medians and variance measures should be available. If there are restrictions on publicly sharing data—e.g. participant privacy or use of data from a third party—those must be specified.

Reviewer #1: Yes

Reviewer #3: Yes

**********

5. Is the manuscript presented in an intelligible fashion and written in standard English?

PLOS ONE does not copyedit accepted manuscripts, so the language in submitted articles must be clear, correct, and unambiguous. Any typographical or grammatical errors should be corrected at revision, so please note any specific errors here.

Reviewer #1: No

Reviewer #3: Yes

**********

6. Review Comments to the Author

Please use the space provided to explain your answers to the questions above. You may also include additional comments for the author, including concerns about dual publication, research ethics, or publication ethics. (Please upload your review as an attachment if it exceeds 20,000 characters)

Reviewer #1: Gendered dimensions of sustainable land management: evidence of farm size and training effects on willingness to pay in Ghana’s Volta Region

PONE-D-25-40765R2

The reviewer reports 3.

Abstract

The abstract's results should directly reflect the stated hypothesis and objectives, emphasizing the impact of gender differences as the primary finding. Revise the abstract conclusion to align more closely with the study's results and hypotheses.

Literature

The hypotheses do not align with the study title. The study investigates gender dimensions using farm size and training effects, but the hypotheses focus on how farm size and training affect willingness to pay (WTP).

Methodology

Review the methodology to determine if the methods are appropriate for the study population, considering cluster or stratified approaches. Focus the study model description on the specific methods used, moving extraneous information about measured variables to the relevant section.

Results

Clearly present the results, addressing each research question. Name and reference tables appropriately. State findings directly and concisely, avoiding methodological explanations.

Conclusion

The main concluding points must be focus on the key question of the study (Gender).

Acknowledgement

Acknowledgments typically express gratitude for professional, academic, and personal support received during the (original) work, progressing from formal acknowledgments (supervisors, funding) to informal ones (peers, family, friends). Directly acknowledge those who provided assistance.

Reviewer #3: I have no further suggestions，The author has already addressed all the issues. It is suggested that the author further revise the entire text and minimize the number of grammatical errors as much as possible.

**********

7. PLOS authors have the option to publish the peer review history of their article (what does this mean?). If published, this will include your full peer review and any attached files.

If you choose “no”, your identity will remain anonymous but your review may still be made public.

**Do you want your identity to be public for this peer review?** For information about this choice, including consent withdrawal, please see our Privacy Policy.

Reviewer #1: **Yes:**Abdoelmoniem Awad Attaelmanan

Reviewer #3: No

**********

[NOTE: If reviewer comments were submitted as an attachment file, they will be attached to this email and accessible via the submission site. Please log into your account, locate the manuscript record, and check for the action link "View Attachments". If this link does not appear, there are no attachment files.]

To ensure your figures meet our technical requirements, please review our figure guidelines: https://journals.plos.org/plosone/s/figures

You may also use PLOS’s free figure tool, NAAS, to help you prepare publication quality figures: https://journals.plos.org/plosone/s/figures#loc-tools-for-figure-preparation.

NAAS will assess whether your figures meet our technical requirements by comparing each figure against our figure specifications.

Attachment Submitted filename: PONE-D-25-40765_R2 Gender dimention with comments.pdf

Attachment Submitted filename: Gendered dimensions report 3.docx

# Author comment: Author response to Decision Letter 3

**DOI:** 10.1371/journal.pone.0351424.r006

1 May 2026

In the introduction, we now state the main contribution of the study more explicitly so that the novelty and significance of the work are clear from the outset. In addition, we have revised the Discussion section to focus more tightly on the key findings, added a brief paragraph describing the study limitations at the end of the conclusions, and reviewed the full manuscript to ensure consistent terminology across all sections.

We hope the revisions satisfactorily addresses your concerns.

Attachment Submitted filename: Authors response to reviewes.docx

# Editor report: Decision Letter 3

**DOI:** 10.1371/journal.pone.0351424.r007

**Authors:** Muhammad Umer Arshad

28 May 2026

Gendered dimensions of sustainable land management: evidences from farm size and training effects on willingness to pay in Ghana’s Volta Region

PONE-D-25-40765R3

Dear Dr. Fiagborlo,

We’re pleased to inform you that your manuscript has been judged scientifically suitable for publication and will be formally accepted for publication once it meets all outstanding technical requirements.

Within one week, you’ll receive an e-mail detailing the required amendments. When these have been addressed, you’ll receive a formal acceptance letter and your manuscript will be scheduled for publication.

An invoice will be generated when your article is formally accepted. Please note, if your institution has a publishing partnership with PLOS and your article meets the relevant criteria, all or part of your publication costs will be covered. Please make sure your user information is up-to-date by logging into Editorial Manager at Editorial Manager® and clicking the ‘Update My Information' link at the top of the page. For questions related to billing, please contact billing support.

If your institution or institutions have a press office, please notify them about your upcoming paper to help maximize its impact. If they’ll be preparing press materials, please inform our press team as soon as possible -- no later than 48 hours after receiving the formal acceptance. Your manuscript will remain under strict press embargo until 2 pm Eastern Time on the date of publication. For more information, please contact onepress@plos.org.

Kind regards,

Muhammad Umer Arshad

Academic Editor

PLOS One

Additional Editor Comments (optional):

Reviewers' comments:

# Editor report: Acceptance letter

**DOI:** 10.1371/journal.pone.0351424.r008

**Authors:** Muhammad Umer Arshad

PONE-D-25-40765R3

PLOS One

Dear Dr. Fiagborlo,

I'm pleased to inform you that your manuscript has been deemed suitable for publication in PLOS One. Congratulations! Your manuscript is now being handed over to our production team.

At this stage, our production department will prepare your paper for publication. This includes ensuring the following:

* All references, tables, and figures are properly cited

* All relevant supporting information is included in the manuscript submission,

* There are no issues that prevent the paper from being properly typeset

You will receive further instructions from the production team, including instructions on how to review your proof when it is ready. Please keep in mind that we are working through a large volume of accepted articles, so please give us a few days to review your paper and let you know the next and final steps.

Lastly, if your institution or institutions have a press office, please let them know about your upcoming paper now to help maximize its impact. If they'll be preparing press materials, please inform our press team within the next 48 hours. Your manuscript will remain under strict press embargo until 2 pm Eastern Time on the date of publication. For more information, please contact onepress@plos.org.

You will receive an invoice from PLOS for your publication fee after your manuscript has reached the completed accept phase. If you receive an email requesting payment before acceptance or for any other service, this may be a phishing scheme. Learn how to identify phishing emails and protect your accounts at https://explore.plos.org/phishing.

If we can help with anything else, please email us at customercare@plos.org.

Thank you for submitting your work to PLOS ONE and supporting open access.

Kind regards,

PLOS ONE Editorial Office Staff

on behalf of

Dr. Muhammad Umer Arshad

Academic Editor

PLOS One
